# The *Zygosaccharomyces bailii* transcription factor Haa1 is required for acetic acid and copper stress responses suggesting subfunctionalization of the ancestral bifunctional protein Haa1/Cup2

**DOI:** 10.1186/s12864-016-3443-2

**Published:** 2017-01-13

**Authors:** Margarida Palma, Paulo Jorge Dias, Filipa de Canaveira Roque, Laura Luzia, Joana Fernandes Guerreiro, Isabel Sá-Correia

**Affiliations:** iBB-Institute for Bioengineering and Biosciences, Department of Bioengineering, Instituto Superior Técnico, Universidade de Lisboa, 1049-001 Lisbon, Portugal

**Keywords:** Yeast, Haa1, Cup2/Ace1, Acetic acid response and tolerance, Copper response and tolerance, Subfunctionalization, Transcription factors

## Abstract

**Background:**

The food spoilage yeast species *Zygosaccharomyces bailii* exhibits an extraordinary capacity to tolerate weak acids, in particular acetic acid. In *Saccharomyces cerevisiae*, the transcription factor Haa1 (ScHaa1) is considered the main player in genomic expression reprogramming in response to acetic acid stress, but the role of its homologue in *Z. bailii* (ZbHaa1) is unknown.

**Results:**

In this study it is demonstrated that ZbHaa1 is a ScHaa1 functional homologue by rescuing the acetic acid susceptibility phenotype of *S. cerevisiae haa1*Δ. The disruption of *ZbHAA1* in *Z. bailii* IST302 and the expression of an extra *ZbHAA1* copy confirmed *ZbHAA1* as a determinant of acetic acid tolerance. ZbHaa1 was found to be required for acetic acid stress-induced transcriptional activation of *Z. bailii* genes homologous to ScHaa1-target genes. An evolutionary analysis of the Haa1 homologues identified in 28 Saccharomycetaceae species genome sequences, including *Z bailii*, was carried out using phylogenetic and gene neighbourhood approaches. Consistent with previous studies, this analysis revealed a group containing pre-whole genome duplication species Haa1/Cup2 single orthologues, including ZbHaa1, and two groups containing either Haa1 or Cup2 orthologues from post-whole genome duplication species. *S. cerevisiae* Cup2 (alias Ace1) is a transcription factor involved in response and tolerance to copper stress. Taken together, these observations led us to hypothesize and demonstrate that ZbHaa1 is also involved in copper-induced transcriptional regulation and copper tolerance.

**Conclusions:**

The transcription factor ZbHaa1 is required for adaptive response and tolerance to both acetic acid and copper stresses. The subfunctionalization of the single ancestral Haa1/Cup2 orthologue that originated Haa1 and Cup2 paralogues after whole genome duplication is proposed.

**Electronic supplementary material:**

The online version of this article (doi:10.1186/s12864-016-3443-2) contains supplementary material, which is available to authorized users.

## Background

The *Saccharomyces cerevisiae* transcription factor Haa1 was first identified based on the DNA binding domain (DBD) homology with the copper-regulated transcription factor Cup2 (alias Ace1) DBD [1]. The paralogue pair Haa1 and Cup2 DBDs comprise 123 and 124 amino acid residues, respectively, at the N-terminal and include a conserved zinc module and a set of four cysteine-cysteine clusters organized in a consensus sequence that forms the copper regulatory domain (CuRD). Such conservation at the level of the DNA binding domains led to hypothesize that, like Cup2, Haa1 could play a role in copper homeostasis; however, metalloregulation and involvement of Haa1 in *S. cerevisiae* tolerance to copper could not be assigned to this transcription factor [1]. Indeed, no physiological function could be ascribed to Haa1 until the description, by our laboratory, of the essential role of Haa1 in *S. cerevisiae* adaptation and tolerance to weak acids, especially to the short-chain hydrophilic acetic and propionic acids [2].

*S. cerevisiae* Haa1 is considered the main player in yeast genomic expression reprogramming in response to acetic acid stress, being involved in the direct, or indirect, transcriptional activation of approximately 80% of the acetic acid-responsive genes, several of which required for maximum tolerance to this weak acid [3, 4]. The Haa1 target genes are involved in transcription, multidrug resistance, cell wall remodelling, metabolism of lipids, carbohydrates and amino acids, and nucleic acid processing [2, 4]. Haa1 binds, in vivo*,* to an acetic acid responsive element (ACRE) in the promoter of its target genes [5]. Among these genes are *TPO2* and *TPO3* that code for two plasma membrane transporters of the Major Facilitator Superfamily proposed to mediate the efflux of acetate from the cell interior in acetic acid challenged yeast cells [2, 4, 5]. Other genes of the Haa1 regulon that are required for tolerance to acetic acid [1, 4], include: *YGP1* (cell wall-related secretory glycoprotein, [6]), *YRO2* (plasma membrane protein with a putative role in acetic acid tolerance [7]), *HRK1* (a protein kinase of a family related with the phosphorylation of membrane proteins and implicated in activation of the activity of plasma membrane H^+^-ATPase Pma1 [8]) and *HSP30* (a plasma membrane heat shock protein proposed as a negative regulator of Pma1 [9]). The involvement of Haa1 in *S. cerevisiae* adaptation and tolerance to acetic acid stress has been demonstrated [2, 4, 5], but the function of *Zygosaccharomyces bailii* Haa1 homologue remains unknown. However, this yeast species is highly problematic in the spoilage of acidic food and beverages due to its remarkable capacity to tolerate acetic acid and other weak acid food preservatives [10]. Although the mechanisms underlying the response and extreme tolerance of *Z. bailii* to acetic acid are still poorly characterized, a number of relevant physiological strategies have been reported. These include the capacity of the yeast cells to tolerate short-term intracellular pH changes [11, 12], co-consume acetic acid and glucose [13–15] and exhibit high basal level of complex sphingolipids proposed to decrease plasma membrane permeability to this weak acid [16]. Also, a recent genome-wide study identified the *Z. bailii* transcription factor ZbMsn4 [17], homologous to the *S. cerevisiae* stress-responsive transcriptional activators Msn4 and Msn2 [18], as an acetic acid tolerance determinant. Other tolerance determinant genes, homologous to *S. cerevisiae GYP8* and *WSC4* (cellular transport and transport routes), *PMT1*, *KTR7* and *RKR1* (protein fate), *TIF3* (protein synthesis) and *ILV3* (amino acid metabolism) were also singled out in the same study [17]. In *S. cerevisiae*, *MSN2* was found to be an acetic acid tolerance determinant [3], and *MSN4* is among the genes activated by Haa1 in response to acetic acid stress [4].

Based on the amino acid sequence similarity of Haa1 and Cup2 DBDs, Keller et al. [1] proposed that *HAA1* and *CUP2* are paralogues. The concept that this paralogy relationship originated in the whole genome duplication (WGD) event was first proposed by Dietrich and co-authors [19], and independently confirmed upon the release of the Yeast Gene Order Browser (YGOB) [20, 21], a database dedicated to the assignment of an orthology/ohnology classification to WGD-originated genes in the Saccharomycetaceae family. Although the identification of the orthologue/ohnologue status is important to trace the evolutionary history of a particular gene family, there are other forces driving the evolution of genes and genomes, as for example local duplications, horizontal gene transfer, gene loss and gain and gene conversion. Understanding the origin of those variations requires the identification of the complete set of bona fide members of a gene family and the integration of different in silico approaches, such as phylogeny and gene neighbourhood analysis.

In the present study we investigate the function of *Z. bailii* gene *ZbHAA1* using functional and evolutionary approaches. Using a blastp network traversal approach to perform a global analysis of all the ORFs encoded in the publicly available genomes of 28 Saccharomycetaceae yeast species, a group of amino acid sequences was identified as showing strong sequence similarity to *S. cerevisiae* Haa1 amino acid sequence. The function of the sole *ZbHAA1* encoded in the genome sequences of *Z. bailii* strains CLIB 213^T^ (mentioned herein as ZYBA_3_9_I00670) [22] and IST302 (ORF ZBIST_2620) (Palma M et al.: Genome sequence of the highly weak-acid-tolerant Zygosaccharomyces bailii IST302, amenable to genetic and physiological manipulations, unpublished) was examined. *Z. bailii* strain IST302 has recently been used by our laboratory because, contrasting with the hybrid strain ISA1307 and *Z. bailii* CLIB 213^T^, it is more prone to genetic engineering and physiological studies ([17], Palma M et al.: Genome sequence of the highly weak-acid-tolerant Zygosaccharomyces bailii IST302, amenable to genetic and physiological manipulations, unpublished). Phylogenetic and gene neighbourhood analyses of the amino acid sequences of the Haa1 homologues identified in Saccharomycetaceae yeasts, led us to confirm that, upon the WGD event [23], the single transcription factor encoded in the protoploid ancestor yeast species has evolved into two distinct gene sub-lineages, each corresponding to the homologues of *S. cerevisiae HAA1* and *CUP2* genes in post-WGD species. In this work we experimentally tested the possibility that both *HAA1* and *CUP2* genes have arisen from the subfunctionalization of the ancestral *HAA1*/*CUP2* orthologue after the WGD event.

## Methods

### Strains and growth media

The prototrophic strains *Zygosaccharomyces bailii* IST302 [17] and the type strain *Z. bailii* ATCC58445^T^ (=CLIB 213^T^) [22] were used in this work. Auxotrophic strains *Saccharomyces cerevisiae* BY4741 (*MATa, his3*Δ*1, leu2*Δ*0, met15*Δ*0, ura3*Δ*0*) and derived deletion mutants *haa1*Δ and *cup2*Δ were obtained from the EUROSCARF collection. Prototrophic yeast strains were batch-cultured at 30 °C with orbital agitation (250 rpm) in liquid mineral medium (MM) that contains, per litre, 1.7 g yeast nitrogen base without amino acids or (NH_4_)_2_SO_4_ (Difco), 20 g D-glucose (Merck) and 2.65 g (NH_4_)_2_SO_4_ (Merck). *S. cerevisiae* auxotrophic strains were cultured in MM4 medium, prepared using MM medium supplemented with 20 mg methionine, 20 mg histidine, 60 mg leucine and 20 mg uracil (all from Sigma). Yeast cells used for yeast transformation experiments were cultured in YPD medium (1% yeast extract, Difco, 2% peptone, Difco, and 2% D-glucose, Merck). For *Z. bailii* transformants recovery, cells were grown in YPF medium (2% D-fructose, Amresco, 1% yeast extract, Difco, and 2% peptone, Difco). Transformant selection after disruption of *ZbHAA1* in the chromosome, or after transformation of *Z. bailii* IST302 with an extra copy of *ZbHAA1*, was performed in plates containing YPF supplemented with 200 μg/mL G418. Selection of transformants from the derived auxotrophic *S. cerevisiae* strains was based on growth in MM4 without uracil supplementation. *Escherichia coli* XL1-Blue was used for plasmid maintenance and general cloning procedures. *E. coli* cells were grown in Luria-Bertani medium (LB), supplemented with 150 mg/L ampicillin when required. Solid media were obtained by adding 20 g of agar to each litre of the corresponding liquid media. All strains were maintained at −80 °C in appropriate media supplemented with 15% glycerol (v/v).

### *ZbHAA1* and *ScHAA1* cloning into pGREG506

The nucleotide sequences of *ZbHAA1* from *Z. bailii* IST302 (ZBIST_2620) and CLIB 213^T^ (ZYBA_3_9_I00670) were obtained from the corresponding genome sequences (Palma M et al.: Genome sequence of the highly weak-acid-tolerant Zygosaccharomyces bailii IST302, amenable to genetic and physiological manipulations, unpublished, and Galeote et al. [22], respectively). The pGREG506 vector from the DRAG & DROP collection [24] was used to individually clone by homologous recombination and expression of the ORFs ZBIST_2620, ZYBA_3_9_I00670 and *ScHAA1*, all under the control of the same promoter region, the *ScHAA1* promoter region. The cloning vector pGREG506 was acquired from EUROSCARF and contains a *HIS3* gene under the control of a galactose inducible promoter (*GAL1*) and the yeast selectable marker *URA3*. The homologous recombination was performed in two steps comprising, first, the cloning of *ScHAA1* promoter into pGREG506 and, second, the cloning of *ScHAA1* or *ZbHAA1* into the recombinant vector obtained during the first step. *ScHAA1* promoter was amplified from strain BY4741 using the primers ScHAA1prom-F and ScHAA1prom-R (Additional file 1). The amplified *ScHAA1* promoter and pGREG506 digested with *SpeI* and *SacI* restriction enzymes, i.e. pGREG506 without the *GAL1* promoter, were co-transformed into *S. cerevisiae* BY4741 parental strain and a recombinant plasmid (pGREGpromScHAA1) was obtained through homologous recombination in yeast. Correct cloning of the promoter was confirmed by DNA sequencing. The ORFs ZBIST_2620, ZYBA_3_9_I00670 and *ScHAA1* were amplified from *Z. bailii* IST302, *Z. bailii* CLIB 213^T^ and *S. cerevisiae* BY4741 genomic DNA, respectively, using the corresponding pair of primers ZbHAA1rec-F/ZbHAA1rec-R and ScHAA1rec-F/ScHAA1rec-R (Additional file 1). Each of the amplified ORFs was co-transformed into *S. cerevisiae* BY4741 with the recombinant vector pGREGpromScHAA1 linearized with *SalI* (i.e. with the *HIS3* gene removed). Correct cloning of the genes into the recombinant plasmids was confirmed by DNA sequencing. These plasmids, named here as pG_ScHAA1, pG_ZbHAA1-IST and pG_ZbHAA1-CL, containing, respectively, *ScHAA1* or *ZbHAA1* homologue from strain IST302 or strain CLIB 213^T^, were then transformed into *S. cerevisiae haa1*Δ deletion mutant. pG_ScHAA1 and pG_ZbHAA1-IST were also transformed in *cup2*∆ deletion mutant. Additionally, *S. cerevisiae* BY4741 parental, *haa1*Δ and *cup2*∆ were transformed with the cloning vector pGREG506 with the *HIS3* gene deleted (pGREG506_noHIS3) that was previously constructed and reported as the correct empty vector to be used as control [17] since in the recombinant vectors the *HIS3* gene is substituted by the insert of interest.

### Disruption of *Z. bailii* IST302 *HAA1* homologue

- Construction of ZbHAA1 disruption cassette

The disruption of *Z. bailii* IST302 *ZbHAA1* (ORF ZBIST_2620) was performed by homologous recombination of a *ZbHAA1* disruption cassette into the chromosome. The disruption cassette was obtained by a three step-based fusion of DNA fragments generated by polymerase chain reaction (PCR) [25, 26]. All PCR reactions were performed with Taq Phusion High-Fidelity DNA Polymerase (Thermo Scientific) following the manufacturer’s instructions. In the first step three primary PCR reactions were carried out. The amplification of fragments *haa1*a and *haa1*b was obtained by using the pair of primers *haa1*a-1/*haa1*a-2 and *haa1*b-3/*haa1*b-4, respectively, with *Z. bailii* IST302 genomic DNA as a template. The *kan cassette* DNA fragment was amplified from the centromeric plasmid pZ_3_ *b*T [27] using the pair of primers *kan*-5/*kan*-6 (Additional file 1). These primers were carefully designed in order to have identical melting temperatures. Accordingly, the same annealing temperature (56 °C) was used in all PCR reactions. The amplified DNA fragments *haa1*a, *haa1*b and *kan cassette* were analysed in a 0.8% agarose (NZYTech) gel electrophoresis and its molecular weight confirmed by comparison with 1 kb Plus DNA Ladder (Invitrogen). The DNA fragments were then directly extracted and purified from the agarose gel using JETQUICK gel extraction spin kit (Genomed) according to manufacturer’s instructions. In the second and third steps we followed the methodology described by Shevchuk et al. [26] with minor modifications. Basically, a PCR reaction of 12 cycles was carried out without primers and containing approximately 100 ng of *haa1*a, 100 ng of *haa1*b and 30 ng of *kan cassette*. Subsequently, a total of 3 μL of this PCR product was used as a template for another PCR reaction (total volume of 20 μL) containing the primers *haa1*a-1 and *haa1*b-4 (Additional file 1). The resulting 2.7 kb PCR product was analysed by electrophoresis in 0.8% agarose, extracted and purified as mentioned above. The methodology was performed several times to obtain 0.5–1 μg of DNA that was subsequently used for transformation of *Z. bailii* IST302 cells.

b) Transformation of *Z. bailii* IST302 and homologous recombination of *ZbHAA1* disruption cassette in the chromosome

The transformation of *Z. bailii* IST302 was performed with Alkali-Cation^TM^ Yeast Transformation kit (MP Biomedicals) introducing minor modifications to the manufacturer’s protocol. Specifically, cells were cultivated overnight in YPD medium, at 30 °C and 250 rpm, and then re-inoculated at an absorbance of 0.1 at 600 nm in 50 ml of fresh medium. Cells were grown until mid-exponential phase (absorbance of 1.0 ± 0.1 at 600 nm) and a total of 1.5x10^8^ cells were collected by centrifugation. All centrifugation steps were carried out during 10 min at 4000 rpm (4 °C). A total of 0.5–1 μg of the *HAA1* disruption cassette was added to the transformation mixture. The transformation reaction was incubated during 30 min at 30 °C, followed by a heat-shock at 42 °C during 40 min. Transformed cells were then centrifuged, resuspended in YPF containing 1 M sorbitol (Sigma) and incubated overnight at 30 °C for recovery. Yeast recombinant clones were selected in YPF plates containing 200 μg/mL G418 (Sigma).

### Cloning and expression of an extra copy of *ZbHAA1* in *Z. bailii* IST302

The expression of an extra copy of *ZbHAA1* gene in the parental strain *Z. bailii* IST302 was performed by cloning this ORF by homologous recombination into the centromeric expression vector pZ_3_ *b*T [27] linearized with *XbaI*. The amplification of *ZbHAA1* and of its corresponding promoter (approximately 1000 bp upstream the start codon) from *Z. bailii* IST302 genomic DNA was performed using the primers ZbHAA1pZ-F, ZbHAA1pZ-R (Additional file 1). The amplified product was co-transformed with the linearized pZ_3_ *b*T into *S. cerevisiae* BY4741. Selection of the transformants harbouring the recombinant vector pZ_3_ *b*T_ ZbHAA1 was performed in YPD plates containing G418 (200 μg/mL). The correct recombination was confirmed by sequencing the obtained plasmids. The recombinant plasmids and the cloning plasmid were used to transform *Z. bailii* IST302, as previously described.

### Susceptibility assays in liquid media

Susceptibility assays in liquid media were assessed by comparing yeast growth curves obtained by periodic measurements of culture absorbance at 600 nm. Yeast cells were pre-cultured in the appropriate unsupplemented medium until mid-exponential phase (standard absorbance of 0.5 ± 0.05) and then reinoculated at an initial absorbance of 0.05, in 50 ml of the fresh medium, supplemented or not with acetic acid. All assays were carried at 30 °C with orbital agitation (250 rpm). Different growth media were used according to strains’ nutritional requirements. The susceptibility to acetic acid of *S. cerevisiae* parental strain BY4741 and the derived deletion mutant *haa1*Δ, harbouring each of the recombinant plasmids produced in this study was assessed in liquid MM4-U medium, pH 4.0, either or not supplemented with 60 mM or 75 mM acetic acid. Acetic acid susceptibility assays involving *Z. bailii* strains were performed in liquid MM medium, pH 4.0, either or not supplemented with 180 and 220 mM acetic acid.

### Susceptibility assays in solid media

Yeast cells were pre-cultured overnight in the appropriate unsupplemented liquid medium and then reinoculated at an absorbance at 600 nm of 0.05, in 50 ml of fresh medium. When the cultures reached an absorbance of 0.5 ± 0.05 at 600 nm cells were resuspended in sterile water to obtain suspensions with an absorbance of 0.05 ± 0.005. These cell suspensions and three subsequent dilutions (1:5; 1:10; 1:20) were applied as 4 μL spots onto the surface of agarized media, supplemented, or not, with increasing concentrations of the compound to be tested and incubated at 30 °C for 3 to 5 days. In the case of *Z. bailii* IST302 parental and derived *Zbhaa1∆* deletion mutant strains, as well as *Z. bailii* IST302 expressing an extra copy of *ZbHAA1* (pZ_3_ *b*T_ZbHAA1) or the cloning vector (pZ_3_ *b*T) susceptibility to acetic, benzoic and sorbic acids and copper was compared in MM medium, at pH 4.5. Regarding *S. cerevisiae* BY4741 parental and derived deletion mutants *haa1*∆ and *cup2*∆ or this *cup2*∆ mutant transformed with the recombinant plasmids produced herein, susceptibility assays were assessed in MM4 or MM4-U media, respectively, at pH 4.5.

### Comparison of mRNA relative levels by real time RT-PCR

The effect of the expression of *ZbHAA1* in the mRNA levels from *S. cerevisiae* Haa1- or Cup2- target genes under acetic acid or copper stresses, respectively, was assessed by expressing the recombinant plasmids pG_ZbHAA1-IST, pG_ScHAA1 and the cloning vector either in *S. cerevisiae haa1*∆ or *cup2*∆ deletion mutants. *S. cerevisiae* BY4741 parental strain transformed with the empty vector was used as a positive control. The effect of *ZbHAA1* in *Z. bailii* IST302 transcriptional response to acetic acid was also assessed by comparing the mRNA levels from *Z. bailii* genes homologous to *S. cerevisiae* Haa1-regulon genes in cells of *Z. bailii* IST302 parental and derived deletion mutant *Zbhaa1∆* strains, in the presence and absence of appropriate concentrations of acetic acid. In both assays, yeast cells were cultivated in the appropriate media until mid-exponential phase (standard absorbance at 600 nm of 0.6 ± 0.05) and then reinoculated (initial absorbance of 0.2 ± 0.01) in 500 mL unsupplemented media. After 1 h of growth 100 mL of cell culture were collected and set as the control condition (Time point 0); simultaneously, acetic acid or copper were added to the remaining 400 mL of cell culture. A total of 100 mL of acetic acid- or copper-stressed cells were harvested 1 and 2 h after the addition of acetic acid or copper (Time points 1 and 2). Cells were collected by centrifugation (8000 rpm, 10 min) at 4 °C, washed twice with cold water and the pellets were frozen in liquid nitrogen and kept at −80 °C until RNA extraction. Gene transcript levels were assessed by real time RT-PCR. Extraction of total RNA was performed using a modified hot phenol method [28]. Purification of RNA and DNA digestion with DNAse was performed using the commercial kit Nucleospin ® RNA (Macherey-Nagel, Germany). Synthesis of cDNA from total RNA samples was performed using the MultiscribeTM reverse transcriptase kit (Applied Biosystems) and the subsequent Real Time PCR step was carried out using SYBR_Green reagents and 7500 Real Time PCR System (Applied Biosystems). Specific primers were designed in Primer Express Software (Applied Biosystems) using gene sequences obtained from *Z. bailii* IST302 genome sequence (Palma M et al.: Genome sequence of the highly weak-acid-tolerant Zygosaccharomyces bailii IST302, amenable to genetic and physiological manipulations, unpublished) (Additional file 2) or *S. cerevisiae* genome database (http://www.yeastgenome.org/). The primer sequences are listed in Additional file 1. Relative values obtained for the expression of each gene either in *Z. bailii* IST302 parental strain or in *S. cerevisiae* BY4741 transformed with the cloning vector cultivated in control conditions (Time point 0) were set as 1 and the remaining values are presented relative to that sample. Relative gene expression between the conditions tested was calculated according to the 2^-∆∆Ct^method [29] using either *ACT1* or *ZbACT1* (in the case of *S. cerevisiae* or *Z. bailii* strains, respectively) as an internal control for input cDNA normalization. Experiments were performed, at least, three times. Results were analysed by two-way ANOVA, considering a *p*-value below 0.05, when comparing the relative mRNA levels in each strain at a given time point.

### Identification of the putative Haa1/Cup2 homologues encoded in the genomes of the Saccharomycetaceae yeast species

The translated ORFs of the 33 sequenced hemiascomycetous yeast strains analysed in this work (Additional file 3) were retrieved from their corresponding genome databases (Table 1) and were compiled in the in-house Genome DB. These 33 hemiascomycetous strains correspond to 28 different species, all taxonomically classified in the Saccharomycetaceae family, in particular the genome sequences of *Z. bailii* CLIB 213^T^ and *Z. bailii* IST302 (to be soon released) [30]. Henceforth, the four letters code shown in Table 1 for species abbreviation will be used to designate both yeast genes and species. The number displayed after the first four letters is used to abbreviate the strain name when the genome of more than one strain from a given species is available. To standardize the annotation used, translated ORFs are represented with small letters. The comparative genomics approach used in this study is based on the sequence clustering of all translated ORFs of the 33 sequenced yeast strains. This required the compilation and organization of a total of 199443 translated ORFs. These translated ORFs were organized into a blast database and compared all-against-all using blastp algorithm made available in blast2 package [31]. The blastp algorithm used a gapped alignment with the following parameters: open gap (−1), extend gap (−1), threshold for extending hits (11) and word size (3). This approach generated a total of 45.5 million pairwise alignments. In order to handle this amount of data, sequence clustering was formulated as a graph traversal problem, where the nodes are the translated ORFs and the edges indicate the existence of pairwise sequence similarity between amino acid sequences. Classification of the translated ORFs into clusters was achieved by breadth-first traversing this network at different e-value thresholds, ranging from E-60 to E-1. The identification of the putative Haa1/Cup2 homologues was achieved by traversing these blastp sub-networks using as starting node the *S. cerevisiae* Haa1 protein.

**Table 1:** Saccharomycetaceae yeast strains examined in this work

Position in respect to WGD	Taxonomic genus in the Saccharomycetaceae family	Species	Strain	Name acronym	Genome Database	Genome Annotation Tool/Source	Total number of Haa1/Cup2 homologues
Post-WGD	Saccharomyces	*Saccharomyces cerevisiae*	S288c	sace_1	SGD	SGD	2
			cen.pk113-7d	sace_3	Genbank	YGAP	2
		*Saccharomyces paradoxus*	Consensus of genome sequences	sapa_1	Sanger	YGAP	2
		*Saccharomyces mikatae*	IFO 1815	sami_1	YGOB	YGOB	2
		*Saccharomyces kudriavzevii*	IFO 1802	saku_1	YGOB	YGOB	2
		*Saccharomyces arboricola*	H-6	saar_1	Genbank	YGOB	2
		*Saccharomyces bayanus*	623-6C	saba_1	SGD	YGAP	2
			MCYC 623	saba_2	SGD	YGAP	2
		*Saccharomyces uvarum*	CBS 7001	sauv_1	YGOB	YGOB	2
	Kazachstania	*Kazachstania africana*	CBS 2517	kaaf_1	YGOB	YGOB	2
		*Kazachstania naganishii*	CBS 8797	kana_1	YGOB	YGOB	2
	Naumovozyma	*Naumovozyma castellii*	CBS 4309	naca_1	YGOB	YGOB	2
			NRRL Y-12630	naca_2	SGD	YGAP	2
		*Naumovozyma dairenensis*	CBS 421	nada_1	YGOB	YGOB	2
	Nakaseomyces	*Candida glabrata*	CBS138	cagl_1	YGOB	YGOB	2
			CCTCC M202019	cagl_2	Genbank	YGAP	2
	Tetrapisispora	*Tetrapisispora phaffii*	CBS 4417	teph_1	YGOB	YGOB	2
		*Tetrapisispora blattae*	CBS 6284	tebl_1	YGOB	YGOB	1
	Vanderwaltozyma	*Vanderwaltozyma polyspora*	DSM 70294	vapo_1	YGOB	YGOB	2
Pre-WGD	Zygosaccharomyces	*Zygosaccharomyces bailii*	IST302	zbist	UD	UD	1
			CLIB 213^T^	zyba_3	Genbank	YGAP	1
		*Zygosaccharomyces rouxii*	CBS 732	zyro_1	YGOB	YGOB	1
	Torulaspora	*Torulaspora delbrueckii*	CBS 1146	tode_1	YGOB	YGOB	1
	Lachancea	*Lachancea kluyvery*	CBS 3082	lakl_1	YGOB	YGOB	1
		*Lachancea thermotolerans*	CBS 6340	lath_1	YGOB	YGOB	1
		*Lachancea waltii*	NCYC 2644	lawa_1	YGOB	YGOB	1
	Kluyveromyces	*Kluyveromyces lactis*	CLIB210	klla_1	YGOB	YGOB	1
		*Kluyveromyces marxianus var. Marxianus*	KCTC 17555	klma_1	Genbank	YGAP	1
		*Kluyveromyces wickerhamii*	UCD 54-210	klwi_1	Genbank	YGAP	1
		*Kluyveromyces aestuarii*	ATCC 18862	klae_1	Genbank	YGAP	1
	Eremothecium	*Eremothecium gossypii*	ATCC 10895	ergo_1	YGOB	YGOB	1
		*Eremothecium cymbalariae*	DBVPG 7215	ercy_1	YGOB	YGOB	1
		*Ashbya aceri*	-	asac_1	Genbank	YGAP	1

*SGD* Saccharomyces Genome Database (http://www.yeastgenome.org/download-data/sequence), Genbank (http://www.ncbi.nlm.nih.gov/genome/browse/); Sanger (http://www.sanger.ac.uk/research/projects/genomeinformatics/sgrp.html), *YGOB* Yeast Gene Order Browser (http://ygob.ucd.ie/), *YGAP* Yeast Genome Annotation Pipeline (http://wolfe.ucd.ie/annotation/), *UD* unpublished data (Palma M. et al.: Genome sequence of the highly weak-acid-tolerant Zygosaccharomyces bailii IST302, amenable to genetic and physiological manipulations, unpublished)

### Phylogenetic analysis and tree construction methods

The amino acid sequences of the gathered translated ORFs were used to build a multiple alignment using MUSCLE software [32]. This multiple alignment was analysed using the Jalview software [33] and processed using the MrBayes [34, 35], a Bayesian Markov chain Monte Carlo (MCMC) package for phylogenetic analysis. The functions made available in the seqinr and ape R packages [36] were used to convert the multiple alignment in fasta format into a nexus file required as input by MrBayes. Metropolis coupling of the MCMC sampling of the target distribution made available by the MPI (Message Passing Interface) version of MrBayes was used to speed up phylogeny computation. MrBayes MPI was set to use one “cold” chain together with 9 heated chains, while the remaining MPI parameters were set to default values. The MCMC simulations used 1200000 generations and two independent runs (each started from two distinct random trees) to confirm parameter convergence of the posterior probability distribution and, hence, assuring convergence into similar phylogenetic trees. The option of estimating the fixed-rate amino acid model made available by MrBayes was used, allowing the MCMC sampler to explore all of the nine available models by regularly proposing new ones (upon parameter convergence, each model contributes to the results in proportion to its posterior probability) and rate variation over sites was assumed to follow a gamma distribution. The maximum likelihood PROTML algorithm made available by the PHYLIP package coupled with bootstrap sampling [37] was used to confirm the results obtained with the MrBayes software suite (Additional file 4). The Dendroscope software was used for tree visualization [38].

### Gene neighbourhood analysis

The package “sqldf” [39] and complementing scripting in R language was used to retrieve fifteen neighbour genes on each side of the query genes as well as the corresponding sequence clustering classification from Genome DB. The rationale of synteny analysis was described before [40, 41].

The existence of synteny between query genes was verified through the analysis of network topology (number of shared neighbour pairs) and the biological information associated with the corresponding edges. Three sources of biological information were used to assess the strength of each neighbour pair connection [40]: i) closeness of the connecting neighbours in relation to the query genes, ii) sequence similarity between connecting neighbours and iii) dimension of the sequence cluster to which the homologous neighbours belong; small dimension of the sequence cluster indicates that it is small the probability that two homologous neighbours are in the vicinity of two query genes by chance.

## Results

### Identification of *S. cerevisiae* Haa1 homologues in *Z. bailii* and in other Saccharomycetaceae yeast species

**Fig. 1:**
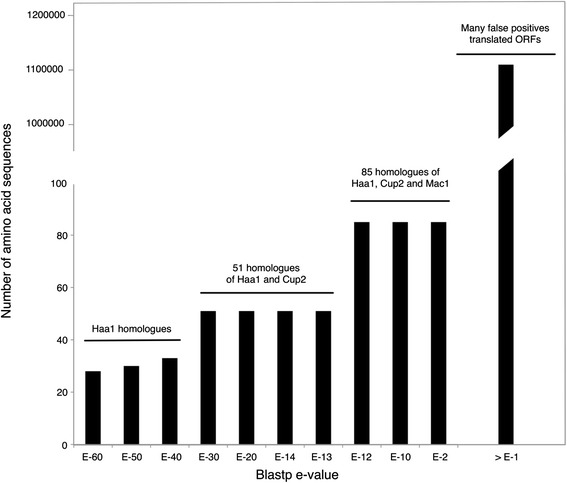
Number of sequences retrieved after constraining and traversing the pairwise similarity network at different e-values using the Haa1 sequence from *S. cerevisia*e S288c as ‘starting node’. This analysis retrieved three successive protein sequence plateaus, depending on the e-value threshold applied, corresponding to the homologues of the *S. cerevisiae* Haa1, Cup2 and Mac1 proteins. An e-value of E-13 was chosen to constrain the pairwise similarity network and 51 amino acid sequences of Haa1 and Cup2 homologues were retained for further evolutionary analyses

The Haa1 protein from *S. cerevisiae* S288c was selected as the starting node for the traversal of the blastp network. The iterative constraining and traversing of this pairwise similarity network at different e-values allowed the identification of the homologues of Haa1 transcription factor encoded in the genome sequences of *Z. bailii* CLIB 213^T^ and IST302 and in other 31 Saccharomycetaceae strains (corresponding to a total of 28 species) (Table 1). The plot representing the number of sequences retrieved at different e-values shows four distinct blastp clustering ranges (Fig. 1). The first range occurs between e-values E-60 to E-40, gathering amino acid sequences highly similar to the starting node Haa1. In the second blastp clustering range (e-values from E-30 to E-13), the amino acid sequences similar to *S. cerevisiae* Cup2 protein are merged with the Haa1 protein set into a single group comprising a total of 51 sequences. In the third blastp clustering range (e-values from E-12 to E-2), the homologues of the *S. cerevisiae* Mac1 transcription factor are merged with the Haa1/Cup2 protein set (a total of 87 amino acid sequences). In the fourth blastp clustering range (e-values bellow E-1) many false positive amino acid sequences were incorporated in the Haa1/Cup2/Mac1 protein set. The cause for the merge of the Mac1 protein set with the Haa1/Cup2 homologues at low blastp e-values is the sharing of copper fist domain in the N-terminal of all these *S. cerevisiae* transcription factors. Considering that Mac1 is remotely similar to the Haa1 and Cup2 proteins, an e-value of E-13 was selected to constrain the pairwise similarity network and the corresponding 51 amino acid sequences were retained for further analysis (Additional file 3). The joint retrieval from the blastp network of both *S. cerevisiae* Haa1 and Cup2 transcription factors support the previous suggestion that the encoding genes constitute a paralogue pair [1, 19, 20] and allowed to conclude that the genome sequences of the two *Z. bailii* strains scrutinized in this study encode a single Haa1 homologue (Table 1) that is also homologous to Cup2. Consistent with the joint retrieval of Haa1 and Cup2 homologues, that in *S. cerevisiae* correspond to two independent transcription factors with 694 and 225 amino acids, respectively, the amino acid sequences herein identified in 28 Saccharomycetaceae species comprise highly heterogeneous protein sizes. Similar to the *S. cerevisiae* Haa1 (ScHaa1), *Z. bailii* sole Haa1/Cup2 homologue (ZbHaa1) has a protein size of 694 amino acid residues.

### Expression of the *HAA1* homologue from *Z. bailii* leads to increased tolerance of *S. cerevisiae* to acetic acid

**Fig. 2:**
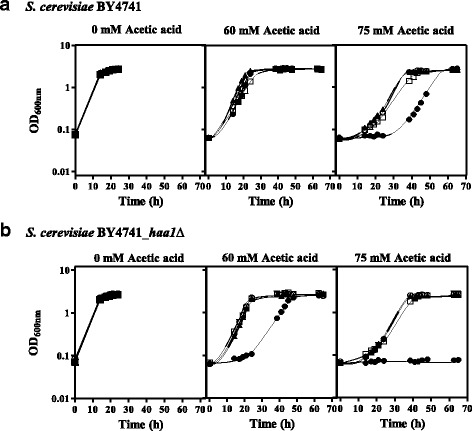
Expression of *HAA1* homologues of *Z. bailii* IST302 and CLIB 213^T^ or *S. cerevisiae* BY4741 from *ScHAA1* natural promoter increases acetic acid tolerance of *S. cerevisiae* BY4741 and derived deletion mutant *haa1*∆. Growth curves of **a** *S. cerevisiae* BY4741 and **b** *S. cerevisiae* BY4741 *haa1*∆ mutant strain expressing the cloning vector pGREG506_noHIS3 (●) or the recombinant plasmids pG_ZbHAA1-IST (▲), pG_ZbHAA1-CL (○) and pG_ScHAA1 (□), in MM4 medium (pH 4.0) without uracil (to keep selective pressure over the recombinant strains), supplemented with 0, 60 or 75 mM of acetic acid. The growth curves are representative of at least three independent growth assays that produced similar results

**Fig. 3:**
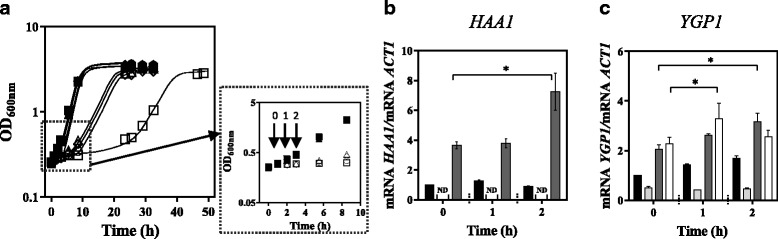
Like ScHaa1, ZbHaa1 is able to mediate the transcriptional activation of *YGP1* in *S. cerevisiae* under acetic acid stress. The effect that the expression of *ZbHAA1* or *ScHAA1* in *S. cerevisiae haa1∆* has in the transcript levels from the ScHaa1 target gene *YGP1* was compared*.* **a** Growth curves of *S. cerevisiae* BY4741 parental strain transformed with the cloning vector (●,○) and of its derived deletion mutant *haa1*∆ expressing the recombinant plasmids pG_ZbHAA1-IST (♦,◇), pG_ScHAA1 (▲,△) or the cloning vector (■, □) cultivated in MM4-U (filled symbols) or in the same medium supplemented with acetic acid (empty symbols). Yeast cells were harvested before acetic acid supplementation (Time point 0), or 1 or 2 h after acetic acid addition (Time points 1 and 2, respectively). **b** Transcript levels from *HAA1* in *S. cerevisiae* BY4741 transformed with the cloning vector (*black bar*), in *S. cerevisiae* deletion mutant *haa1*∆ transformed with the cloning vector (ND-not detected), or expressing *ScHAA1* (*dark grey bar*). **c** Transcript levels from *YGP1* in *S. cerevisiae* BY4741 transformed with the cloning vector (*black bar*), in *S. cerevisiae* deletion mutant *haa1*∆ transformed with the cloning vector (*light grey bar*), or expressing *ScHAA1* (*dark grey bar*) or *ZbHAA1* (*white bar*). *ACT1* mRNA level was used as an internal control. The mRNA levels from *HAA1* and *YGP1* genes in *S. cerevisiae* BY4741 transformed with the cloning vector in the absence of acetic acid (Time point 0) were set as 1 and the transcript levels from those genes were calculated relative to this control. mRNA values shown are the mean of, at least, three independent experiments. Results were analysed by two-way ANOVA. (*) *p*-value below 0.05, when comparing the relative mRNA levels in each strain at a given time point

To investigate the suggested role of the *HAA1* homologues from two *Z. bailii* strains in acetic acid tolerance, the susceptibility to acetic acid of *S. cerevisiae* BY4741 and derived deletion mutant *haa1*Δ strains expressing either *ZbHAA1* or *ScHAA1* was compared (Fig. 2). Two *ZbHAA1* gene alleles were considered in this analysis, one from *Z. bailii* IST302 (ORF ZBIST_2620, Additional file 2 (Palma M et al.: Genome sequence of the highly weak-acid-tolerant Zygosaccharomyces bailii IST302, amenable to genetic and physiological manipulations, unpublished) and the other from *Z. bailii* CLIB 213^T^ [22]. Both nucleotide sequences share 99.7% identity, with the corresponding encoded proteins having three amino acid substitutions. These *ZbHAA1* genes were cloned into pGREG506 under the control of *S. cerevisiae* BY4741 *HAA1* promoter. *S. cerevisiae* BY4741 *HAA1* gene sequence was also cloned into the same vector, under the control of its native promoter and used as an internal control for comparative expression experiments. The expression of either *ScHAA1* or the two *ZbHAA1* genes in *S. cerevisiae* BY4741, cultivated in supplemented media with 60 or 75 mM acetic acid, significantly reduced the duration of the latency phases and increased the specific growth rates when compared with the host strain harbouring the cloning vector (Fig. 2a, Table 2). Interestingly, the increase of acetic acid tolerance of *S. cerevisiae* parental strain was maximal when *ZbHAA1* from the more tolerant yeast strain *Z. bailii* IST302 (Palma M et al.: Genome sequence of the highly weak-acid-tolerant Zygosaccharomyces bailii IST302, amenable to genetic and physiological manipulations, unpublished) was expressed (Table 2). Also, the heterologous expression of *ZbHAA1* gene alleles in *haa1*Δ mutant strain was found to rescue the susceptibility phenotype of the deletion mutant by increasing its tolerance to 60 mM acetic acid up to the level of the *haa1*Δ mutant expressing *ScHAA1* gene (the duration of the latency phase is reduced of approximately 30 h) (Fig. 2b; Table 2). Differences registered in acetic acid tolerance in the parental strain harbouring the cloning vector and in the *haa1*∆ mutant expressing *ScHAA1* (Fig. 2; Table 2) are likely the result of *ScHAA1* expression from a centromeric vector [24] and not from the chromosomal locus. In fact, the mRNA levels from *ScHAA1* in *haa1*∆ expressing *ScHAA1* from the recombinant vector are above those in the parental strain transformed with the cloning vector, both in the absence (Time point 0) or presence of acetic acid stress (Time points 1 and 2) (Fig. 3a). However, the mRNA levels from *ZbHAA1* in *haa1*∆ expressing *ZbHAA1* could not be calculated because the parental strain used as a reference strain for the calculation of relative mRNA levels does not harbour the *ZbHAA1* gene (Fig. 3b).

**Table 2:** Duration of the lag phase of growth (h) and maximum specific growth rates (μ_max_ (h^−1^))

	0 mM Acetic acid		60 mM Acetic acid		75 mM Acetic acid	
Strain	Lag phase (h)	μ_max_ (h^−1^)	Lag phase (h)	μ_max_ (h^−1^)	Lag phase (h)	μ_max_ (h^−1^)
BY4741 + pGREG506_noHIS3	-	0.22 ± 0.016	12.7 ± 3.4	0.22 ± 0.007	35.6 ± 5.4	0.15 ± 0.010
BY4741 + pG_ScHAA1	-	0.22 ± 0.013	8.3 ± 2.2	0.16 ± 0.004	15.4 ± 1.8	0.12 ± 0.004
BY4741 + pG_ZbHAA1-CL	-	0.21 ± 0.023	7.2 ± 2.1	0.20 ± 0.010	11.9 ± 1.0	0.13 ± 0.002
BY4741 + pG_ZbHAA1-IST	-	0.23 ± 0.016	6.3 ± 2.3	0.21 ± 0.018	10.8 ± 0.4	0.13 ± 0.001
*haa1*∆ + pGREG506_noHIS3	-	0.23 ± 0021	37.1 ± 5.0	0.16 ± 0014	NG	NG
*haa1*∆ + pG_ScHAA1	-	0.23 ± 0014	7.9 ± 1.2	0.18 ± 0013	15.5 ± 1.9	0.13 ± 0002
*haa1*∆ + pG_ZbHAA1-CL	-	0.23 ± 0013	8.1 ± 0.9	0.21 ± 0010	14.3 ± 0.0	0.15 ± 0001
*haa1*∆ + pG_ZbHAA1-IST	-	0.24 ± 0015	9.1 ± 1.2	0.19 ± 0017	12.9 ± 0.7	0.14 ± 0005

Duration of the lag phase of growth (h) induced by acetic acid stress and maximum specific growth rates (μmax (h^-1^)) of *S. cerevisiae* BY4741 and derived deletion mutant *haa1*∆ expressing *ZbHAA1* from *Z. bailii* strains IST302 (pG_ZbHAA1-IST) and CLIB 213^T^ (pG_ZbHAA1-CL), *S. cerevisiae HAA1* (pG_ScHAA1) or the cloning vector (pGREG506_noHIS3). Mean and standard deviation values of at least three independent experiments are indicated. (−) No lag phase detected; (NG) No growth detected during the course of the experiment (65 h)

### ZbHaa1 is involved in the regulation of *S. cerevisiae* acetic acid-responsive gene *YGP1*

The mRNA levels from *YGP1* gene in *S. cerevisiae*, whose transcriptional activation under acetic acid stress is dependent on Haa1 [4], *w*ere compared in *S. cerevisiae haa1*Δ mutant expressing either *ScHAA1* or *ZbHAA1* and in the parental strain and derived *haa1*Δ mutant transformed with the cloning vector (Fig. 3c). As expected, the relative mRNA levels from *YGP1* gene in the *haa1*∆ mutant were below the levels in the parental strain, both in the absence of acetic acid (Time point 0) or following sudden exposure to acetic acid (Time points 1 and 2). An increase in mRNA levels from *YGP1* gene was detected in the *haa1*Δ mutant expressing *ZbHAA1* or *ScHAA1*, respectively after 1 and 2 h of acetic acid supplementation of the growth medium, but no activation was detected in *haa1*∆ harbouring the cloning vector (Fig. 3c). These results indicate that ZbHaa1 is also able to mediate the transcriptional activation of *YGP1* in *S. cerevisiae* cells challenged with acetic acid stress.

### *ZbHAA1* is required for *Z. bailii* tolerance to several weak acids

**Fig. 4:**
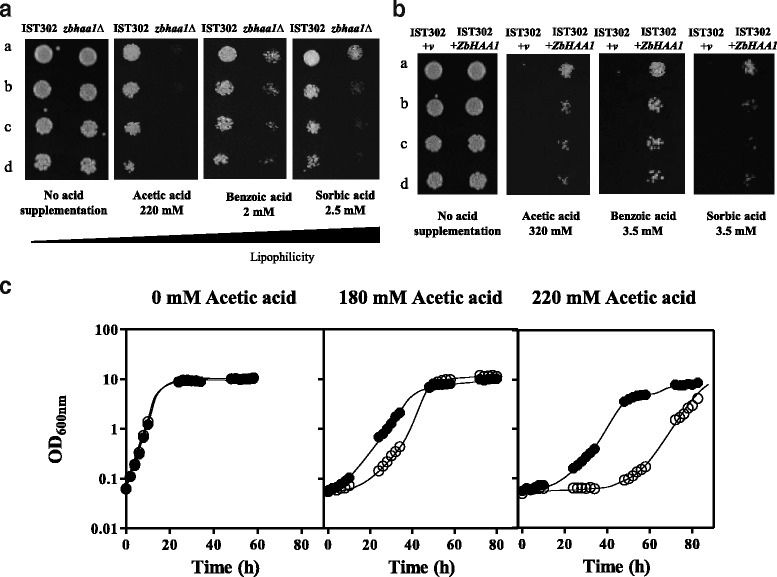
The *ZbHAA1* is a determinant of tolerance to acetic acid in *Z. bailii*. **a** Growth of *Z. bailii* IST302 and derived mutant *Zbhaa1∆* was compared by spot assays in MM medium (pH 4.5) supplemented, or not, with equivalent concentrations of acetic, benzoic and sorbic acids (i.e. concentrations that induced similar growth inhibition in strain IST302). **b** Growth of *Z. bailii* IST302 harbouring the cloning vector pZ_3_ *b*T (+v) and *Z. bailii* IST302 expressing an extra copy of *ZbHAA1* (+*ZbHAA1*) was compared by spot assays in MM medium (pH 4.5) supplemented, or not, with concentrations of the different weak acids that impaired growth of *Z. bailii* IST302 (+v). The images depicted in **a**) and **b**) were taken, respectively, after 4 or 5 days of incubation at 30 °C and are representative of at least three independent experiments. For both **a**) and **b**), cell suspensions with an absorbance at 600 nm of 0.05 ± 0.005 (lane a) and subsequent dilutions of 1:5, 1:10 and 1:20 (lanes b, c and d, respectively) were spotted onto the surface of agarized medium; **c** Growth curves of *Z. bailii* IST302 expressing an extra copy of *ZbHAA1* (●) or harbouring the cloning vector pZ_3_ *b*T (○). Growth was performed in MM medium (pH 4.0) supplemented with 0, 180 or 220 mM acetic acid. Growth curves are representative of at least three independent growth assays that produced similar results

To examine the hypothesized role of the expression of *ZbHAA1* in *Z. bailii* tolerance to acetic acid, a *Zbhaa1∆* deletion mutant was prepared in *Z. bailii* IST302 and the susceptibility of the parental strain and derived mutant to acetic acid, as well as to other weak acids used as food preservatives (benzoic and sorbic acids), was compared by spot assays (Fig. 4a). Using equivalent concentrations of the different weak acids or, in other words, concentrations that induce a similar duration of growth latency of the parental strain, it was possible to demonstrate that *ZbHAA1* expression is required for increased *Z. bailii* tolerance to the three weak acid food preservatives tested, but *ZbHAA1* protective effect was more evident under acetic acid stress (Fig. 4a). In fact, no cell growth was detected in the *Zbhaa1∆* mutant strain after 4 days of incubation with 220 mM acetic acid, whereas this mutant strain was still able to resume growth after a latency of approximately 4 days when cultivated in the presence of equivalent concentrations of the more lipophilic benzoic and sorbic acids. This result is in line with results reported before for the effect of *HAA1* in *S. cerevisiae* indicating that maximal protection is exerted against the short-chain length more hydrophilic weak acids [2].

Weak acid concentrations that impaired growth of *Z. bailii* IST302 harbouring the cloning vector were used to compare the tolerance of *Z. bailii* IST302 expressing an extra copy of *ZbHAA1* (Fig. 4b) that led to a remarkable increase of *Z. bailii* tolerance to the three weak acids (Fig. 4b). The enhanced tolerance to acetic acid of *Z. bailii* IST302 expressing an extra copy of *ZbHAA1* was also confirmed in liquid MM medium supplemented with 220 mM acetic acid at pH 4.0, where a decreased duration of the latency phase of approximately 30 h was observed (Fig. 4c). In summary, *ZbHAA1* is an important player in *Z. bailii* tolerance to acetic acid and other weak acid food preservatives.

### ZbHaa1 is required for acetic acid-induced transcriptional activation of *Z. bailii* genes homologous to *S. cerevisiae* Haa1-target genes

**Fig. 5:**
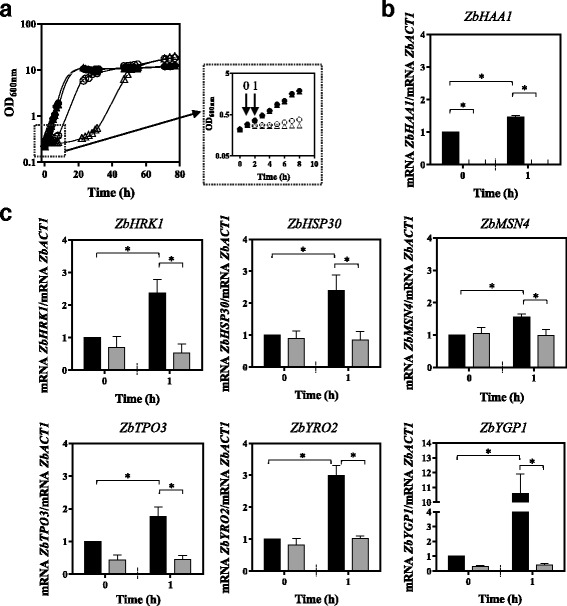
ZbHaa1 is required for acetic acid induced transcriptional activation of *Z. bailii* genes demonstrated to be homologous to *S. cerevisiae* Haa1 target genes. **a** Growth curves of *Z. bailii* IST302 (●,○) and of its derived deletion mutant *Zbhaa1∆* (▲,△) cultivated in MM medium, pH 4.0 (filled symbols) or in the same medium supplemented with acetic acid (empty symbols). Acetic acid was added after 1 h of growth of exponential cells prepared under standard conditions in MM medium to a final concentration of 140 mM. Yeast cells were harvested before acetic acid supplementation (Time point 0) and 1 h after acetic acid addition (Time point 1). Comparison of the transcript levels from **b** *ZbHAA1*, and **c** *ZbMSN4*, *ZbHRK1, ZbHSP30*, *ZbTPO3*, *ZbYGP1* and *ZbYRO2* in *Z. bailii* IST302 (dark bars) or in the derived deletion mutant *Zbhaa1∆* (grey bars) in the absence of acetic acid (Time point 0) and 1 h after acetic acid supplementation (Time point 1). *ZbACT1* mRNA level was used as an internal control. The mRNA level from *Z. bailii* IST302 genes *ZbHAA1*, *ZbMSN4*, *ZbHRK1*, *ZbHSP30*, *ZbTPO3*, *ZbYGP1* and *ZbYRO2* in the absence of acetic acid (Time point 0) were set as 1 and the transcript levels from those genes were calculated relative to this control. mRNA values shown are the mean of, at least, three independent experiments. Results were analysed by two-way ANOVA. (*) *p*-value below 0.05, when comparing the relative mRNA levels in each strain at a given time point

The effect of the presence of acetic acid stress and the expression of *ZbHAA1* in the transcription levels from six *Z. bailii* genes homologous to *S. cerevisiae* genes whose transcription was previously reported to be dependent on Haa1 regulation under acetic acid stress [4], was examined (Fig. 5). The nucleotide sequences of the referred *Z. bailii* genes, homologous to *S. cerevisiae* Haa1 target genes, specifically *ZbHRK1*, *ZbHSP30*, *ZbTPO3*, *ZbMSN4*, *ZbYGP1* and *ZbYRO2,* are available in Additional file 2. The mRNA levels from the selected putative ZbHaa1 targets in *Z. bailii* IST302 were found to increase during the early adaptive response to the acid, specifically 1 h after sudden exposure to acetic acid, this activation being significantly reduced in *Zbhaa1∆* deletion mutant (Fig. 5). These results indicate that the activation of the selected genes in response to acetic acid stress is dependent on ZbHaa1, and suggest that the Haa1 regulon is similar in *S. cerevisiae* and *Z. bailii* species.

### Phylogenetic analysis of the Haa1 and Cup2 homologues in Saccharomycetaceae yeasts

**Fig. 6:**
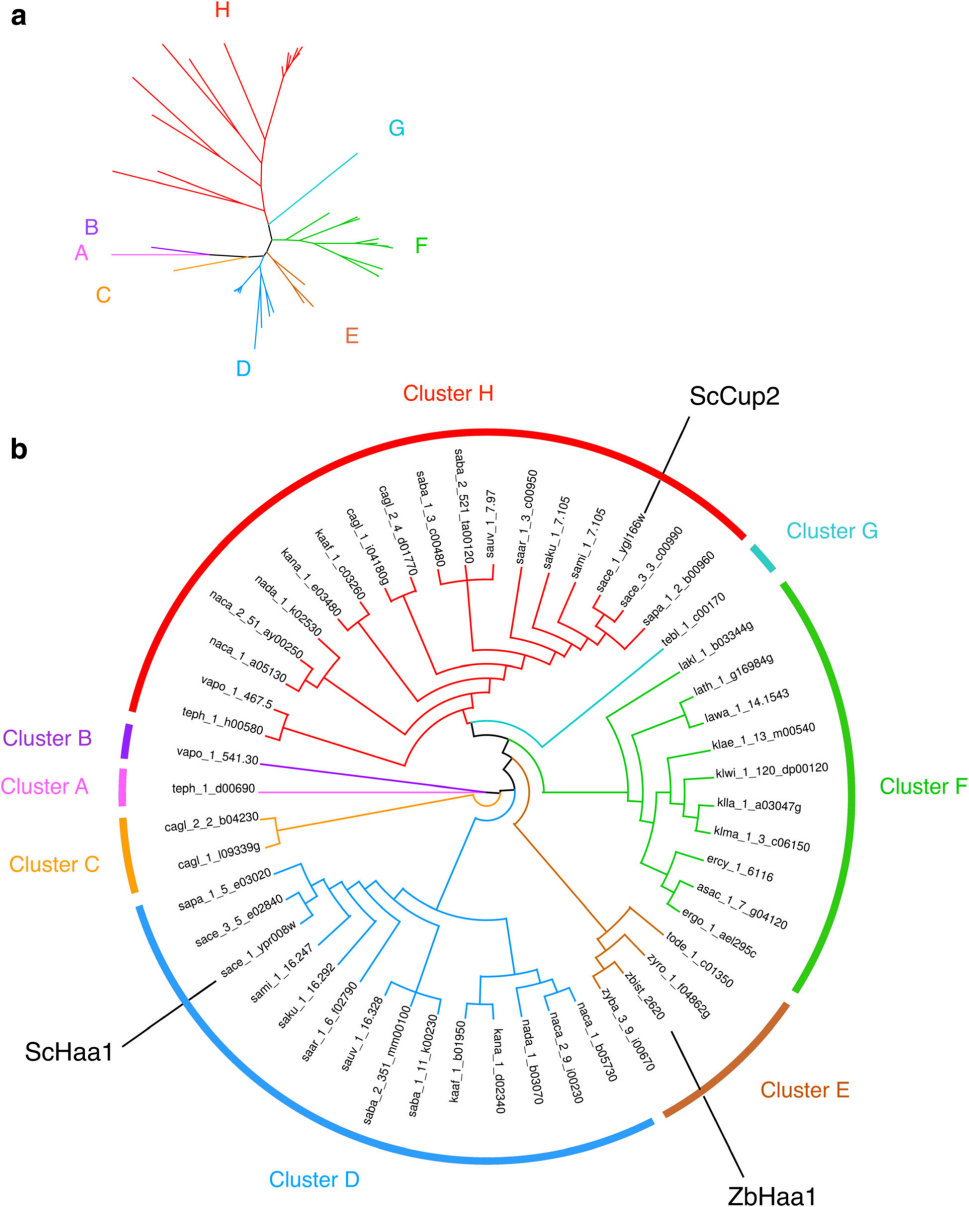
Phylogenetic analysis of *S. cerevisiae* Haa1 and Cup2 transcription factors homologues encoded in the genomes of 33 strains of 28 yeast species belonging to the Saccharomycetaceae family. **a** Radial phylogram showing the amino acid sequence similarity distances between these 51 full-size proteins. **b** Circular cladogram showing the tree topology, with the name of the *S. cerevisiae* and *Z. bailii* members indicated. MrBayes software suite was used in phylogenetic tree calculation using simulation statistical options described in materials and methods. The gene and species annotation adopted in this study uses the four letters code described in Table 1. The ORF teph_1_h00580, was chosen as outgroup due to the strong dissimilarity of its amino acid sequence when compared with those of the remaining members of this gene family

Using the multiple alignment of the amino acid sequences of the putative Haa1 and Cup2 homologues of 33 strains from 28 pre- and post- WGD species, including *Z. bailii* CLIB 213^T^ and IST302 sequences, as input, MrBayes phylogenetic software suite was used to construct a phylogenetic tree representing this family of transcription factors with copper-fist binding domain. After running two MCMC simulations for 1200000 generations, the standard deviation of split frequencies was below 0.01 and the potential scale reduction factor (PSRF) was reasonably close to 1.0 for all parameters, indicating their convergence [42, 43]. The parameters of the Gamma distribution, assumed being equal by MrBayes, converged into a value of 1.145, with the 95% Highest Posterior Density (HPD) interval ranging from 1.037 to 1.262, while the total tree length of the phylogenetic tree converged into a value of 37.112 (95% HPD interval = 34.139–40.085). The corresponding consensus Bayesian phylogenetic tree was retained for further analysis. A maximum likelihood (ML) approach made available by the PhyML software suite was used to confirm the phylogenetic relationships between the Haa1 and Cup2 homologues. The credibility of the phylogenetic clades was confirmed by inspection of the bipartition probabilities and bootstrapping values calculated for each internal node of the Bayesian and ML trees, respectively. Since the Bayesian and the ML statistical approaches originated similar phylogenetic trees regarding cluster composition and credibility (Fig. 6, Additional file 4), the analysis of the phylogenetic relationships between these transcription factors made in this study is based only in the tree obtained with MrBayes.

The analysis of the multiple alignment of the Haa1 and Cup2 transcription factor homologues revealed that their transactivation domain is poorly conserved in the Saccharomycetaceae yeast species under study, being responsible for the long branches observed in the corresponding tree (Fig. 6). This phylogenetic tree is divided into eight clusters, labelled from A to H. The translated ORF teph_1_d00690 was chosen as outgroup of this phylogenetic tree (cluster A) due to the strong dissimilarity of its amino acid sequence when compared with the ones of the remaining members of this gene family. The division of the Haa1 and Cup2 homologues into the proposed phylogenetic clusters was based on the following criteria: 1) protein length and 2) the taxonomic classification of the yeast species encoding each putative transcription factor. Cluster H comprises only proteins ranging from 221 to 356 amino acids (260 amino acids in average) and the remaining clusters comprise proteins ranging from 525 to 893 amino acids (671 amino acids in average). Nevertheless, the analysis of the amino acid and nucleotide sequences confirmed that all the phylogenetic clusters comprise full-size proteins, being free of fragments, pseudogenes and other type of sequence artefacts. The protein size and the analysis of the amino acid sequence similarity relationships shown by these transcription factors suggests that cluster H comprises the orthologues of *S. cerevisiae* Cup2 and clusters A, B, C, D and G comprise the orthologues of *S. cerevisiae* Haa1. With the exception of *Tetrapisispora blattae*, the genomes of the yeast species that have diverged after the WGD event encode two proteins belonging to this gene family, each one corresponding to the Haa1 or the Cup2 orthologues. Reinforcing the notion that these two *S. cerevisiae* genes are paralogues with origin in the WGD event [1, 19, 20] is the fact that the genomes of the protoploid Saccharomycetaceae species analysed in this study encode a single Haa1/Cup2 homologue (clusters E and F). Consistently, the genomes of the yeast species classified in the *Zygosaccharomyces* taxonomic genus under analysis in this study encode a single Haa1/Cup2 homologue: *Z. bailii* IST302 (ZBIST_2620), *Z. bailii* CLIB 213^T^ (ZYBA_3_9_I00670) and *Z. rouxii* CBS 732 (ZYRO_1_F04862g).

All-against-all Needleman-Wunsch alignments were constructed to cross-compare the amino acid sequence similarity between the possible combinations of these three groups of proteins: Haa1 orthologues (clusters A, B, C, D and G), Cup2 orthologues (cluster H) and Haa1/Cup2 orthologues encoded in the protoploid Saccharomycetaceae genomes (clusters E and F). The amino acid sequence of the Haa1 and Cup2 orthologues showed, in average, 29.7% and 15.0% identity with Haa1/Cup2 protoploid orthologues, respectively (with the pairwise alignment comprising 63.5% and 31.7% of gaps, respectively). On the other hand, the comparison between the Haa1 orthologues versus the Cup2 orthologues showed that these share, in average, 12.7% identity between the corresponding amino acid sequences (and 67.4% of gaps). Considering that the protein size of the post-WGD Cup2 orthologues is less than one half of the size shown by the post-WGD Haa1 orthologues and of the protoploid Haa1/Cup2 orthologues, it is likely that the lower sequence identity between Cup2 orthologues and the members of other phylogenetic clusters is essentially due to the heterogeneous size and gap introduction in the pairwise sequence alignments.

### Reconstruction of the evolutionary history of Haa1 and Cup2 orthologues based on gene neighbourhood analysis

**Fig. 7:**
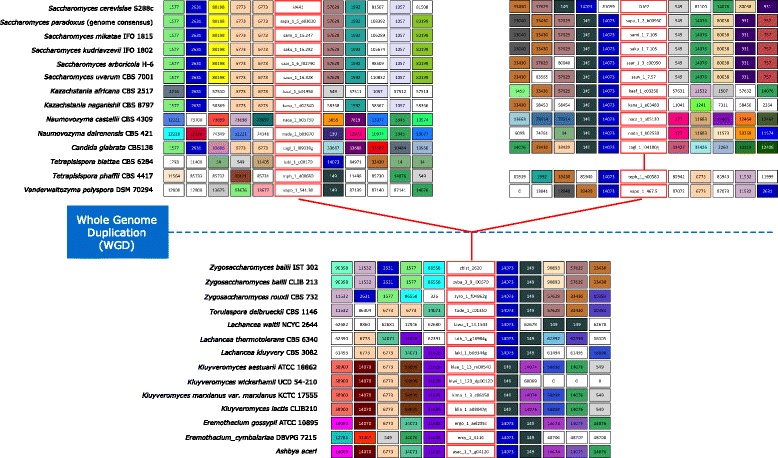
Gene neighbourhood analysis of the *S. cerevisiae HAA1* and *CUP2* orthologues from species of the Saccharomycetaceae family. Central boxes represent *S. cerevisiae HAA1* and *CUP2* orthologues. Adjacent boxes represent their gene neighbours. Homologous gene neighbours are highlighted using the same colour and identified with the same number. A white box represents genes with no homologous neighbours in the represented chromosome region. The synteny was assessed with 15 neighbours on each side, but for the sake of clarity this representation was truncated to 5 neighbours (see Additional file 5 for full neighbourhood details)

**Fig. 8:**
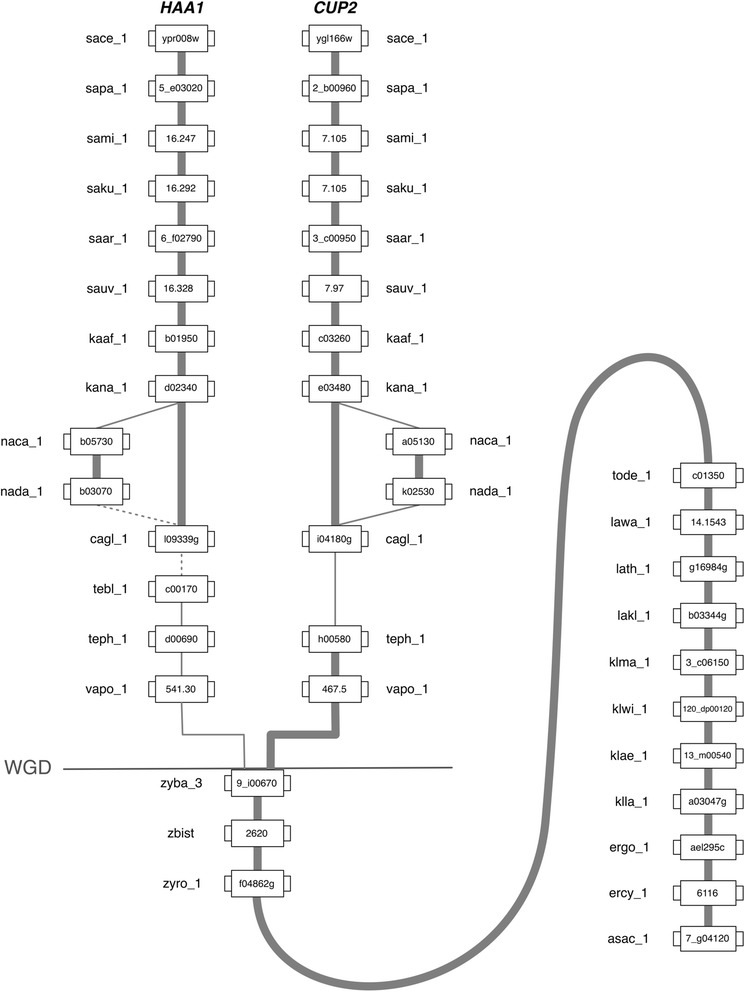
Reconstruction of the evolutionary history of the Haa1 and Cup2 orthologues in Saccharomycetaceae yeasts. Each box represents a gene. Lines connect genes sharing common neighbours. Thick lines connecting genes indicate the existence of stronger synteny evidence. The line labelled as WGD indicates the point in time in the evolution of the Saccharomycetaceae yeast where the Whole Genome Duplication (WGD) event occurred

The grouping of each of the ORFs into a specific cluster of amino acid sequence similarity allows the exploitation of the information on the chromosome environment where the *HAA1* and *CUP2* orthologues reside. Although the synteny information comprised in the YGOB database [20] already allows such analysis for most of the yeast species examined in this study, the genome sequences of *Z. bailii* species are still lacking in this database as well as the sequences of other species with the genomes currently available (*S. paradoxus*, *S. arboricola*, *S. bayanus*, *Kluyveromyces marxianus var. Marxianus*, *K. wickerhamii*, *K. aestuarii* and *Ashbya aceri*). For this reason, and since the combination of phylogenetic and comparative genomic analyses is a more reliable strategy to elucidate the orthologue/paralogue status of homologous genes, the analysis of the chromosome environment where all the *HAA1* and *CUP2* orthologue sequences reside was performed. Results confirm that these genes share many common neighbours (Fig. 7). Considering that putative Haa1/Cup2 transcription factors of protoploid Saccharomycetaceae species always show a protein size above 540 amino acids (average size of 622 amino acids), it is likely that the *HAA1*/*CUP2* ancestral gene would encode a protein with size closer to the *HAA1* orthologues. The gene neighbourhood analysis corroborated the indications obtained from classic phylogenetic methodologies that after the WGD event, a single gene lineage comprising the members of the Haa1/Cup2 family gave rise to two sub-lineages, each comprising the orthologues of *S. cerevisiae HAA1* and *CUP2* genes (Figs. 7 and 8). With the exception of the yeast species from *Naumovozyma* genus, all orthologues encoded in late-divergent post-WGD species share strong synteny (i.e. inside each sub-lineage). However, *Vanderwaltozyma* and *Tetrapisispora* species, belonging to early divergent taxonomic genera shortly differentiated after the WGD event, shows that their *HAA1* and *CUP2* orthologues share scarce synteny with the *S. cerevisiae HAA1* and *CUP2* genes and with other genes encoded in post-WGD species that evolved later, suggesting local genome-shuffling.

### The single Haa1/Cup2 orthologue from *Z. bailii* is also involved in response and tolerance to copper stress

**Fig. 9:**
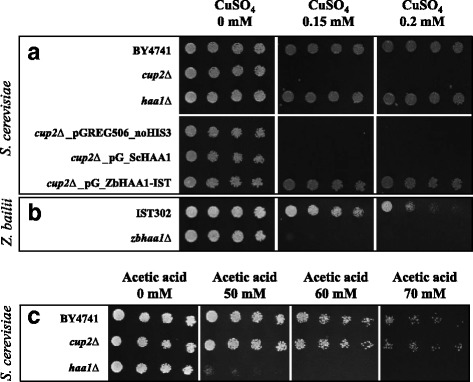
ZbHaa1 is required for copper tolerance. Susceptibility to copper was compared in **a** *S. cerevisiae* BY4741 parental and derived deletion mutants *haa1*∆ and *cup2*∆, in *cup2*∆ expressing the recombinant plasmids pG_ZbHAA1-IST, pG_ScHAA1 or the cloning vector and in **b** *Z. bailii* IST302 parental and derived deletion mutant *Zbhaa1∆* strains. **c** *S. cerevisiae* BY4741 parental and derived deletion mutants *haa1*∆ and *cup2*∆ were also compared in what concerns their susceptibility to acetic acid. Cell suspensions with an absorbance at 600 nm of 0.05 ± 0.005 (lane a) and subsequent dilutions of 1:5, 1:10 and 1:20 (lanes b, c and d, respectively) were spotted onto the surface of the appropriate agarized media, supplemented with appropriate concentrations of copper or acetic acid. The images depicted were taken after 3 days of incubation at 30 °C and are representative of at least three independent experiments

As previously demonstrated by Keller et al. [1], both *S. cerevisiae* BY4741 and derived *haa1*∆ mutant strains are more tolerant to CuSO_4_ than *cup*2∆ mutant strain that is very susceptible to this metal cation (Fig. 9a). On the other hand, *S. cerevisiae* BY4741 parental and derived *cup2*∆ mutant strains are remarkably more tolerant to acetic acid than *haa1*∆ mutant (Fig. 9c). Considering the proposed evolutionary history of Haa1 and Cup2 orthologues and their specific physiological functions in *S. cerevisiae*, the hypothesis of whether the sole ZbHaa1 could be involved in copper tolerance was tested. The heterologous expression of *ZbHAA1* in *S. cerevisiae cup2*Δ mutant strain was found to rescue the susceptibility phenotype of the deletion mutant by increasing its tolerance to CuSO_4_ up to the level of the parental strain (Fig. 9a). Contrasting with these phenotypes, no growth was detected in minimal medium containing 0.15 mM CuSO_4_, either in *S. cerevisiae cup2*Δ transformed with the cloning vector or in the same background strain expressing *ScHAA1* (Fig. 9a). The susceptibility of *Z. bailii* IST302 and derived deletion mutant *Zbhaa1∆* to copper stress was also tested. Differently from the absence of alteration of the copper susceptibility phenotype by the deletion of *ScHAA1* in *S. cerevisiae*, the deletion of *ZbHAA1* in *Z. bailii* IST302 led to a marked increased susceptibility to copper (Fig. 9b). These results reinforce the concept that ZbHaa1 might have a dual function, being putatively involved in the regulation of acetic acid (as demonstrated above) and copper-responsive genes as found for its *S. cerevisiae* single orthologues Haa1 and Cup2, respectively.

**Fig. 10:**
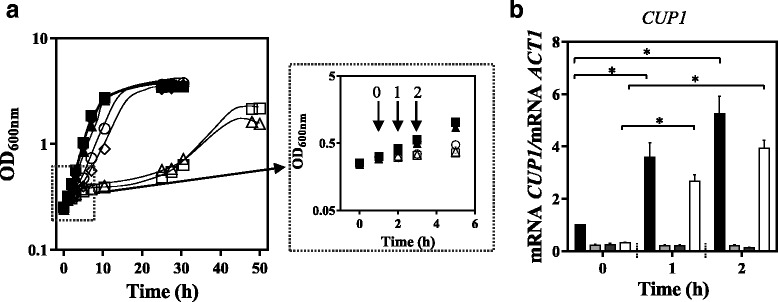
The expression of *ZbHAA1*, but not of *ScHAA1* gene, in *S. cerevisiae cup2∆* mutant leads to the activation of transcription from *S. cerevisiae* Cup2 target gene *CUP1.* **a** Growth curves of *S. cerevisiae* BY4741 parental strain transformed with the cloning vector (●,○) and of its derived deletion mutant *cup2*∆ expressing the recombinant plasmids pG_ZbHAA1 (♦,◇), pG_ScHAA1 (▲,△) or the cloning vector (■, □) cultivated in MM4-U (filled symbols). CuSO_4_ was added after 1 h of growth in MM4-U to a final concentration of 0.1 mM (empty symbols). For mRNA levels quantification, yeast cells were harvested before CuSO_4_ supplementation (Time point 0), or 1 or 2 h after the addition of CuSO_4_ (Time points 1 and 2, respectively). **b** Comparison of the transcript levels from *S. cerevisiae* gene *CUP1* in time points 0, 1 and 2. *ACT1* mRNA level was used as an internal control. The mRNA level from *CUP1* gene in *S. cerevisiae* BY4741 transformed with the cloning vector (*black bar*) in the absence of CuSO_4_ (Time point 0) was set as 1 and the transcript levels from *CUP1* gene were calculated relative to this control. *CUP1* transcript levels in *cup2*∆ transformed with the cloning vector, pG_ScHAA1 or pG_ZbHAA1-IST are represented by light grey, dark grey or white bars, respectively. mRNA values shown are the mean of, at least, three independent experiments. Results were analysed by two-way ANOVA. (*) *p*-value below 0.05, when comparing the relative mRNA levels in each strain at a given time point

**Fig. 11:**
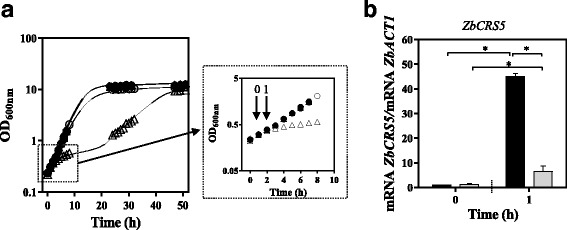
ZbHaa1 is required for the transcriptional activation of *ZbCRS5* gene in copper stressed cells of *Z. bailii*. **a** Growth curves of *Z. bailii* IST302 (●,○) and of its derived deletion mutant *Zbhaa1∆* (▲,△) cultivated in MM medium (filled symbols) or in the same medium supplemented with CuSO_4_ (empty symbols). CuSO_4_ was added after 1 h of growth in MM medium to a final concentration of 0.08 mM. Yeast cells were harvested before CuSO_4_ supplementation (Time point 0) or 1 h after CuSO_4_ addition (Time point 1). **b** Comparison of the transcript levels from *ZbCRS5* in *Z. bailii* IST302 (*dark bars*) or in the derived deletion mutant *Zbhaa1∆* (*grey bars*) in the absence of CuSO_4_ (Time point 0) and 1 h after CuSO_4_ supplementation (Time point 1). *ZbACT1* mRNA level was used as an internal control. The mRNA level from *Z. bailii* IST302 gene *ZbCRS5* in the absence of CuSO_4_ (Time point 0) was set as 1 and the transcript levels from this gene were calculated relative to this control. mRNA values shown are the mean of, at least, three independent experiments. Results were analysed by two-way ANOVA. (*) *p*-value below 0.05, when comparing the relative mRNA levels in each strain at a given time point

In order to confirm the hypothesized role of ZbHaa1 in the response to copper, the transcript levels from *S. cerevisiae* metallothionein encoding gene *CUP1,* that is regulated by Cup2 under copper stress [44], were compared in *S. cerevisiae cup2*Δ mutant strain expressing *ZbHAA1*, in *S. cerevisiae* parental strain (positive control) and in the derived deletion mutant *cup2*Δ transformed with the cloning vector or expressing *ScHAA1* (both used as negative controls). An increase in the mRNA level from *CUP1* was observed in cells expressing *ZbHAA1* after 1 and 2 h of exposure to 0.1 mM CuSO_4_ (Fig. 10). This increase was not detected in *S. cerevisiae cup2*Δ mutant strain either transformed with the cloning vector or expressing *ScHAA1*, demonstrating that *S. cerevisiae CUP1* gene is regulated by ZbHaa1 under copper stress. Since no homologue of *S. cerevisiae CUP1* could be identified in *Z. bailii* IST302 genome sequence, the homologue of *S. cerevisiae* metallothionein encoding gene *CRS5*, whose expression is dependent on *CUP2* upon copper stress [45], was identified in the genome sequence of *Z. bailii* IST302 (ORF ZBIST_3713, here mentioned as *ZbCRS5*, Additional file 2). A remarkable increase in the mRNA levels from *ZbCRS5* in *Z. bailii* IST302 parental strain was found during the early adaptive response to copper (1 h after sudden exposure to copper), being this activation of *ZbCRS5* transcription significantly reduced in the *Zbhaa1∆* deletion mutant (Fig. 11), indicating that the activation of *ZbCRS5* is dependent on ZbHaa1 in *Z. bailii* cells under copper stress.

## Discussion

The physiological role of *Z. bailii* transcription factor ZbHaa1, homologous to *S. cerevisiae* Haa1, in the response and tolerance to acetic acid and copper was examined in this work by exploring functional and evolutionary analyses. It was demonstrated that ZbHaa1 is a functional homologue of ScHaa1, playing a role in *Z. bailii* tolerance to acetic acid and to other weak acid food preservatives. As previously reported for ScHaa1, the effect of ZbHaa1 is more evident for the more hydrophilic short-chain weak acid acetic acid [2]. Moreover, similarly to the increased tolerance to acetic acid resulting from the overexpression of *ScHAA1* in *S. cerevisiae* [46], the expression of an extra copy of *ZbHAA1* in *Z. bailii* remarkably increases the tolerance of *Z. bailii* to all the weak acids tested. Furthermore, the involvement of ZbHaa1 in the transcriptional activation in response to acetic acid stress of *Z. bailii* genes homologous to *S. cerevisiae* Haa1-target genes reinforces the concept that ZbHaa1 is indeed a functional homologue of ScHaa1 and that the ZbHaa1-regulon is, at least in part, similar to the ScHaa1-regulon described before [4].

The identification of *S. cerevisiae* Haa1 homologues in Saccharomycetaceae yeast species showed that *Z. bailii* and all the other pre-WGD species do possess a sole Haa1 homologue that is also homologous to *S. cerevisiae* Cup2 [1], a transcription factor involved in yeast response and tolerance to high copper concentrations [47]. The phylogenetic and gene neighbourhood analyses of the Haa1 and Cup2 homologues identified among 33 strains of 28 pre- and post-WGD Saccharomycetaceae yeast species allowed outlining their evolutionary history. A clear separation of the Haa1 and Cup2 homologues into three main groups resulted from the analysis: two groups containing either Haa1 or Cup2 orthologues encoded in the genomes of post-WGD yeast species, and a third containing Haa1/Cup2 orthologues from pre-WGD species. The only exception to the evolutionary pattern observed, in which post-WGD yeast species encode two members of the Haa1/Cup2 family in their genomes, is *Tetrapisispora blattae* that only possesses in the genome one member of this family. Since the genome sequencing was performed with high coverage (above 20X) and assembled into few scaffolds (10), it is likely that this yeast species may indeed encode a single member of this gene family. However, based on the size of the encoded protein (604 amino acids) and on its position in the phylogenetic tree, it was assigned as a Haa1 orthologue, although two neighbour gene families that also reside adjacent to the chromosomal region where the majority of Cup2 orthologues reside were found in its neighbourhood.

The evolutionary analysis performed reinforces the conclusion of previous studies suggesting that *S. cerevisiae* Haa1 and Cup2 are paralogues with origin in the WGD event [1, 19, 20]. After a gene duplication event, functional redundancy of the two duplicates is frequently avoided for their preservation in the genome and, eventually, fixation in the population gene pool [48–50]. Altogether, the indications obtained in this study led us to hypothesize that the sole *ZbHAA1* gene could encode a bifunctional transcription factor controlling both the acetic acid and copper stress-response regulons that, upon the WGD event, subfunctionalized and partitioned the independent control of each regulon. In this study, experimental evidences are provided indicating that ZbHaa1 is indeed also involved in *Z. bailii* response and tolerance to copper stress. Moreover, the expression of *ZbHAA1* in *S. cerevisiae cup2*∆ mutant leads to the transcriptional activation of the metallothionein encoding gene *CUP1* that in *S. cerevisiae* is activated upon copper stress under the dependence of Cup2 transcription factor [44]. Moreover, *ZbHAA1* expression in *Z. bailii* significantly increases the transcription of *ZbCRS5*, a homologue of *S. cerevisiae CRS5* gene that is also regulated by Cup2 transcription factor in copper-challenged cells [45]. The fact that all genomes of the protoploid yeast species examined in this study encode a single Haa1/Cup2 orthologue suggests that the single Saccharomycetaceae yeast ancestral would have been a bifunctional transcription factor.

It is recognized that the duplication of transcription factors and/or of their gene targets is one of the most important evolutionary forces driving the evolution of transcriptional regulatory networks, leading to the expansion of existent networks or to the emergence of new ones, as well as allowing the acquisition of novel patterns of gene expression [50–52]. This study shows that the *CUP2* orthologues experienced a series of deletion events throughout the evolution of the post-WGD species that led to a progressive decrease in the protein size. This reduction occurred essentially at the level of the transactivation domain while the DNA-binding domain remained highly conserved. Hence, the average protein size of 622 amino acids observed in the Haa1/Cup2 orthologues encoded in the genomes of the protoploid Saccharomycetaceae yeasts was reduced to a total of 341 amino acids in the Cup2 orthologue encoded in the genome of *V. polysporus*, a species that descended from an ancestral yeast that diverged immediately after the WGD event [53]. Further deletions reduced the length of these proteins in late-diverging yeast species, with the *S. cerevisiae* Cup2 transcription factor showing a total size of 225 amino acids. These results support the hypothesis that after the WGD event this bifunctional ancestral gene divided its functions among the daughter duplicates through the acquisition of neutral degenerative mutations. Under this hypothesis, the observed indels in the amino acid sequences of the Cup2 orthologues would be explained by a specific subfunctionalization model, known as Duplication, Degeneration and Complementation [54]. Other cases of subfunctionalization occurring in yeast and consistent with this model have been reported in the literature, for instance, for the paralogue pairs Gal1 and Gal3 [55], Sir2 and Hst1 [56] and Bat1 and Bat2 [57]. The completion of the subfunctionalization process that was in the origin of Haa1 and Cup2 is still unclear. Our results and those reported by Keller et al. [1] show that Haa1 is not involved in *S. cerevisiae* tolerance to copper. Although Cup2 was recently identified as a determinant of acetic acid tolerance in a quantitative trait loci analysis of pooled segregants that revealed the polygenic nature of high acetic acid tolerance in yeast, suggesting that in superior segregrants the *CUP2* allele could take over the function of *HAA1* [58], for *S. cerevisiae* S288c and under the experimental conditions used, we could not find evidences supporting the involvement of Cup2 in acetic acid tolerance. Our study points towards the evolution of both paralogues in a way that each one regulates a subset of the target genes all regulated by the single ancestral bifunctional transcription factor, given that ZbHaa1 activates *Z. bailii* genes homologous to both *S. cerevisiae* Haa1 and Cup2 target genes under acetic acid or copper-induced stresses. However, apparently, no cross-talk has been observed between both regulators in *S. cerevisiae* [1]. *S. cerevisiae* Haa1 and Cup2 transcription factors recognize different motifs in promoter regions, respectively, 5’-(G/C)(A/C)GG(G/C)G-3’[5] and 5’-TC(T)_4-6_GCTG-3’ [59] or 5’-HTHNNGCTGD-3’ [60]. In this study it is demonstrated that ZbHaa1 can activate *S. cerevisiae* Haa1 and Cup2 target genes under acetic acid or copper stresses, respectively, which suggests that ZbHaa1 is apparently able to bind to the promoter regions of all those target genes. We have examined the promoters of the *Z. bailii* genes found in our study to be activated by ZbHaa1 under acetic acid or copper-induced stresses using the YEASTRACT database [61] and identified, in the promoter region of ZbHaa1 target genes *ZbHRK1*, *ZbHSP30*, *ZbMSN4*, *ZbYRO2* and *ZbCRS5,* the presence of both *S. cerevisiae* Haa1 and Cup2 binding sites, but in *ZbTPO3* and *ZbYGP1* only the *S. cerevisiae* Haa1 recognition motif was found. Interestingly, the *S. cerevisiae* promoters of the Haa1 target genes *YGP1*, *MSN4* and *HSP30* and of the Cup2 target gene *CRS5* also hold the binding sites for both Cup2 and Haa1 transcription factors. In order to understand how ZbHaa1 transcription factor regulatory network in *Z. bailii* relates with those regulated in *S. cerevisiae* by Haa1 and Cup2 transcription factors, it is crucial to determine the full ZbHaa1 regulon in *Z. bailii* and to elucidate the regulatory patterns of the target genes under copper or acetic acid-induced stresses. To get this information, it would be necessary at first to compare the transcriptomes of the parental *Z. bailii* strain and the mutant deleted for the gene encoding ZbHaa1 under those stresses and, subsequently, to fully identify the DNA motif(s) recognized by this transcription factor. Such information would provide insights for the understanding on how the DNA binding sequences have changed throughout evolution, and the mechanisms underlying the higher and lower specificity of the different orthologue proteins for the corresponding DNA binding motifs and subsequent transcriptional activation.

## Conclusions

Based on functional and evolutionary analyses, this study demonstrates that the *Z. bailii* transcription factor homologous to *S. cerevisiae* Haa1 (ZbHaa1) is a bifunctional transcription factor, being required for response and tolerance to both acetic acid and copper stresses, and suggests the subfunctionalization of the ancestral Haa1/Cup2 orthologue after whole genome duplication, originating Haa1 and Cup2 paralogues. In *S. cerevisiae,* these genes were described as being individually involved in tolerance to acetic acid and copper stresses, respectively.

## Additional files

Additional file 1: Primers used in this work. (PDF 288 kb)
Additional file 2: Nucleotide sequences of the *Zygosaccharomyces bailii* IST302 genes studied. (PDF 105 kb)
Additional file 3: Amino acid sequences of the Haa1 and Cup2 homologues identified in the 28 Saccharomycetaceae yeast species. (PDF 60 kb)
Additional file 4: Phylogenetic analysis of *S. cerevisiae* Haa1 and Cup2 transcription factors homologues encoded in the genomes of 33 strains of 28 yeast species belonging to the Saccharomycetaceae family. A) Radial phylogram showing the amino acid sequence similarity distances between these 51 full-size proteins. B) Circular cladogram showing the tree topology, with the name of the *S. cerevisiae* and *Z. bailii* members indicated. PhyML software suite was used in phylogenetic tree calculation. The gene and species annotation adopted in this study uses the four letters code described in Table 1. (PDF 1465 kb)
Additional file 5: Gene neighbourhood analysis of the *S. cerevisiae HAA1* and *CUP2* orthologues from species of the Saccharomycetaceae family. Central boxes represent *S. cerevisiae HAA1* and *CUP2* orthologues. Adjacent boxes represent their gene neighbours. Homologous gene neighbours are highlighted using the same colour and identified with the same number. A white box represents genes with no homologous neighbours in the represented chromosome region. (PDF 2076 kb)

## Abbreviations

ACRE: Acetic acid responsive element
CuRD: Copper regulatory domain
DBD: DNA binding domain
HPD: Highest Posterior Density
LB: Luria-Bertani
MCMC: Markov chain Monte Carlo
ML: Maximum likelihood
MM: Mineral medium
MPI: Message Passing Interface
ORF: Open reading frame
PCR: Polymerase chain reaction
RT-PCR: Reverse transcription polymerase chain reaction
SGD: Saccharomyces genome database
WGD: Whole genome duplication
YGAP: Yeast genome annotation pipeline
YGOB: Yeast gene order browser
YPD: Yeast extract-peptone-dextrose
YPF: Yeast extract-peptone-fructose
