# Supplementary material for: The Zygosaccharomyces bailii transcription factor Haa1 is required for acetic acid and copper stress responses suggesting subfunctionalization of the ancestral bifunctional protein Haa1/Cup2
Source: BMC Genomics. 2017 Jan 13;18:75. doi: 10.1186/s12864-016-3443-2 (PMC5234253; doi:10.1186/s12864-016-3443-2)
Supplement: Additional file 1: — Primers used in this work. (PDF 288 kb) [file 12864_2016_3443_MOESM1_ESM.pdf]

Additional file 1. Primers used in this work.

NAME	SEQUENCES (5'→3')
**Cloning of *ZbHAA1* and *ScHAA1* into pGREG506 (1)**	
ScHAA1prom-F	*AACAAAAGCTGGAGCTCGTTTAAACGGCGCGCC*CCTACTAAATTATCAATCCTTGCAATTAGCTTC
ScHAA1prom-R	*TGTCGACGGTATCGATAAGCTTGATATCGAATTC*TATTTTTTAGGTTTTTTTTTTCTATACTTTTGTTTCC
ScHAA1rec-F	*GAATTCGATATCAAGCTTATCGATACCGTCGACA*ATGGTCTTGATAAATGGCATAAAGTATGCC
ScHAA1rec-R	*GCGTGACATAACTAATTACATGACTCGAGGTCGAC*TCATAACGAAGACATGAAATTATCCAAATCC
ZbHAA1rec-F	*GAATTCGATATCAAGCTTATCGATACCGTCGACA*ATGGTGTTGATAAACGGTGTCAAG
ZbHAA1rec-R	*GCGTGACATAACTAATTACATGACTCGAGGTCGAC*TCACAATGTGGACATGAAATCG
**Construction of *ZbHAA1* disruption cassette (2)**	
*haa1*a-1	*ATGGTGTTGATAAACGGTGTC*
*haa1*a-2	**CGAGGCAAGCTAAACAGA***TGATATTGAGGCGAGTGAAGG*
*haa1*b-3	**GGCGTAATCATGGTCATAGC***CGTAACCATCAATCCCTCCAT*
*haa1*b-4	*TCACAATGTGGACATGAAATCG*
kan-5	*CCTTCACTCGCCTCAATATCA***TCTGTTTAGCTTGCCTCGTC**
kan-6	*ATGGAGGGATTGATGGTTACG***GCTATGACCATGATTACGCC**
**Cloning of *ZbHAA1* (ORF ZBIST_2620) into pZ$_3$*b*T (3)**	
ZbHAA1pZ-F	*TTTCCCCGAAAAGTGCCACCTGGTATCGGA*CATCCTCTGTATCGCAGTAGGTG
ZbHAA1pZ-R	*GGAAAAACGTTCATTGTTCCTTATTCAGTTAG*TCACAATGTGGACATGAAATCGTC
**Comparison of mRNA relative levels by Real Time RT-PCR in *S. cerevisiae***	
ACT1-F	CTCCACCACTGCTGAAAGAGAA
ACT1-R	CCAAGGCGACGTAACATAGTTTT
CUP1-F	ATGAAGGTCATGAGTGCCAA
CUP1-R	CATTTGTCGTCGCTGTTACAC
YGP1-F	TGTACAATGTTGCCCGTGTTG
YGP1-R	GGCACCGGCGGATGA
YRO2-F	TGGATCCCAGTCAGAGCAAAGT
YRO2-R	ACCTGGGTGCTCCTTTTGG
TPO2-F	TGAGTGATCAAGAATCTGTTG
TPO2-R	CGGTACGGTTCAATTGCTTT
**Comparison of mRNA relative levels by Real Time RT-PCR in *Z. bailii***	
ZbACT1-F	TCACGTTGTGCCAATCTATGC
ZbACT1-R	CGGCCAAGTCGATTCTCAAG
ZbYGP1-F	CCTTCTGCGGGTGAAACTTC
ZbYGP1-R	GCTGGCATCAGAGGCGATAC
ZbMSN4-F	ACACAGCGACACGGATTCAA
ZbMSN4-R	GTGATCCATCCAGCACTGACA
ZbYRO2-F	CCTCGCTACTGGGCAACACT
ZbYRO2-R	CGTGTTAGCCAAACCAACATTG
ZbTPO3-F	GTGACCAATGACCCGGAAAA
ZbTPO3-R	AACCGTATGCCACGCAGATC
ZbHRK1-F	CGGACTCACTGCATTCTGCAT
ZbHRK1-R	GACCCCATAAACTCCTGCAAAC
ZbHAA1-F	TCGGTGCTGCATTCTCATCA
ZbHAA1-R	TCCTGCCTTGCTTTTGTTGA
ZbCRS5-F	CTAGCTGTACTGGTAAGGGATGCA
ZbCRS5-R	GATCACCGCAGCTGCTACATT

(1) These primers contain a region with homology with *ScHAA1* promoter (underlined) and a region with homology with pGREG506 vector (represented in italic).
(2) These primers contain a region with homology to *ZbHAA1* (represented in italic) and a region with homology to the flanking region of KANMX4 (represented in bold).
(3) These primers contain a region with homology with pZ$_3$*b*T vector (represented in italic) and a sequence underlined, which corresponds to the sequence 1000bp upstream ZBIST_2620 (primer ZbHAA1pZ-F) and

the 3’ end of ZBIST_2620 (ZbHAA1pZ-R)
