# Supplementary material for: The Zygosaccharomyces bailii transcription factor Haa1 is required for acetic acid and copper stress responses suggesting subfunctionalization of the ancestral bifunctional protein Haa1/Cup2
Source: BMC Genomics. 2017 Jan 13;18:75. doi: 10.1186/s12864-016-3443-2 (PMC5234253; doi:10.1186/s12864-016-3443-2)
Supplement: Additional file 2: — Nucleotide sequences of the Zygosaccharomyces bailii IST302 genes studied. (PDF 105 kb) [file 12864_2016_3443_MOESM2_ESM.pdf]

Additional file 2. Nucleotide sequences of the *Zygosaccharomyces bailii* IST302 genes studied

**Gene name (ORF) [GenBank® accession number]**	**Sequence**
ZbHAA1 (ZBIST_2620)<br>[KX656024]	ATGGTGTTGATAAACGGTGTCAAGTACGCCTGTGAAAGATGTATAAGGGGCCATAGAGTT<br>ACCACTTGCAACCACACAGATCAGCCTCTGATGATGATTAAGCCGAAGGGGAGGCCATCC<br>ACTACGTGTAACCACTGCAAGGAGTTGAGGAAAAACAAGAACGCCAACCCGTCGGGCGTA<br>TGTACGTGCGGGCGACTCGAGAAGAAACGGCTGGCGCAGAAGGCCAAGGAGGAAGCTCGA<br>GCTAAGGCGAAGGAAGAGAAAAAACTACACGAGTGCCGTTGCGGATATGACGAGCCATGC<br>AGATGCCATTCCAGCAGGAGGAGGCACCAGTCAAGGAAAGTATCGGTGAATGGTAAGGGC<br>AGCAGTTCGAGGCCAGTGTCTCAGCAGTTCGACGCAAATAACCACGTGATGTCACCAGTG<br>TCGATGGATTCGAGCTCCAGTATGCATAACGTCAGTACTTCAGGCAACGGGAAGTCCACC<br>GAAAGCTCGGGTATGCTAGCTTCTGGTTTTCTAGATACAGAGGTTGGAAACGGCGGCAAA<br>GTGTCCAAAGAGTTTCATCAGGTGCCTTCACTCGCCTCAATATCATCACTGCATTCAGGG<br>CAATCATTCGACCAAAAGGTTAATCTTCCACAATCTCCTCTTCTAGGAGGAGGTGGTTCA<br>CTTGGTTTTTTGAATGGCGGTGGTGGAAATCGCAACAGTGGAAATTTTACAAATTGGGGG<br>GACGAGGCGTCACTGTATTCTGTCAGATCTGATTCTAAAGTTAACCTGACGGAGCATAGT<br>GGAGTTCTCACGGGAATGCAGCCTCCCTCAAGTAGTGTTCCAAAGAGAACCTCTTCCACA<br>AAGGCACAGCTGGGTAATGTCAAGGTACCGTTGGAGGAGTATCTGTCCCCTTCAGACAAC<br>TTTAATTCTAAGATAAATGAGACGAGTTCTCCTATGCAGGACTGGTATTTTGAGAGGACT<br>CCTAATGAAGACCCCAATATCTCTGATCAGTTACTTGGAGTGGACGGCAGTGGCGGCGAT<br>GCTGGCGCTAGCCTTCAAGCAGCGCAAAATAGTGGGCTTTTAGACATGTTTATGGACTCT<br>TCGACGATTCCAATTCTGTCAAAAAGCAGTTTACTCATGCAGGACAAATTAGGCCCGGCC<br>GTAGGTAATAACACTAACAACTGTACTACTAGCCCTTCAAATGGTCCCAAAGGGAGAACT<br>TGGAGTAGCCCTATGGCTGCTGATAAATCGGATACTCTTAGTGTAGATGGAGAAAGCGTC<br>AGGAGCGTTGAAGCATTGTCATTAATACCGAGTTACATGGATATCCCAGACAGGGCTCCC<br>AGCCTACACAACGTCCAATCGGTGCTGCATTCTCATCAACACCAACCAGGACATTTCAAT<br>CAACAAAAGCAAGGCAGGAGGTCAGGCAGTGTCAGCAGGAGTCACAGACCTCCCCCACCT<br>ACGTCCAGGAGCGGCGTTCCCGTAACCATCAATCCCTCCATGGTTAGTAGCATCGACGAC<br>ACAATTAGCGTAAATTCGCTGCAAAGTCCCACAGGTTCCGTCCTCGATAACCACAGTTTG<br>TCTACTCCCCTCAGCAATGGGGCAGACTATGGATCTTGCAGACCGGAAAGAGTGAGATCA<br>CCGCTCTTGGGATTGTCAGAGAACAACCCAACGTCTCCACAATTTTATCAACAACAACCT<br>CAAGTTTTGCAATCAGCGACTGGCCCATCAGACTCGGAACTGGATCAGTTACTAGGATTC<br>GACACTGGTAATAATAGTGGCATCATGGACAATACAAACTATTCAGATAAAAATTATGGC<br>AATTTTAATCCGAGTGCTAACATTAACCTCAACGGCAACCGCAATGGAAACGGTAATGTA<br>AGTAGCAACAGCAACGGGAGCAGGAACGGGAGCGGGAGCGGAAACGGCAACAGTAATGGT<br>AACCTGTTATTCCAAAACGACATGAATACTTTATCAAGCCCAGCAATGAAGGATGTTCTC<br>CAAGACACGTCACCTATGAGTAGCAACCAGACTGTCTCGCCACCGAGTCAGTTACTCACG<br>GAAAAAGGTTTTGCCGATTTGGACGATTTCATGTCCACATTGTGA
ZbHRK1 (ZBIST_0481)<br>[KX656025]	ATGCCTAATTTACTGTCGAGGAGACCGTTCCACCATGAACATCACCATCATCATGAACATTCGC<br>CGAATTCCAAACACTCTAGATCGTTTCTCAGCTTCGCACACAAAAGGCAAAGCAACGACTCAAT<br>AAAGAGTGACAAGTCCACGGACTCACTGCATTCTGCATACTCCCCTCATGGTCAGGGCCAAGG<br>GTTCAGTTTGCAGGAGTTTATGGGGTCGTCGTCGCCCAAGTCCTCACATTCGATGGTTGAGCT<br>AAAGAGGTTCCTGAGGCCAAAGATCAACCGCAAGTCGTCTGGAAGTTCGGGACATCACCACCG<br>CGGTGGAGTGAGGCCCTCTGCGTCAACTGTGGAGCTGCCACAGAACTTGCTGCAGCAGTCGC<br>AGCAGTACGCAAAGTCGAATTCTCAGGAGGCAGCTGATCACCATGCACATACGCAATCTGCTA<br>TACCGCCTAGCAGCGACAGTACGCTTTCGCTTTCGAACAAGGTCAATATCTACCATGACGATTC<br>GATACTGGCACAGAAGTACGGAAAGCTCGGTCGTGTGTTGGGTTCTGGTGCTGGTGGTAGCGT<br>CAAGGTGTTGTGGAGACCGAGTGACAATGCTACTTTTGCTGTGAAAGAGTTCCGTCCCCGTAA<br>GCAAAACGAATCTGTCAAGGATTATGCAAAGAAGTGCACAGCTGAATTCTGTATCGGTTCGACG<br>CTGCGTCATTGTAACATCATTGAGACCATGGATATCTTCAGCGATACCAACCAGAATAGGTATTT<br>TGAGGTGATGGAGTATGCGCCGGTAGACTTCTTTGCCGTTGTCATGACAGGCAAAATGTCTAG<br>GGGCGAGATCAATTGCTGCCTGAAACAGATTATGGAAGGTGTCAAATACTTACACTCTTTGGGG<br>CTGGCCCACAGAGACCTTAAACTCGATAACTGCGTGATGACGCTCGATGGTATCTTAAAACTGA<br>TTGATTTCGGTAGTGCCGTGGTATTCAGATATCCGATGGAAGTCGGAGTAACGCCAGCGCATG<br>GCATTGTTGGCAGTGACCCTTACTTAGCGCCAGAGGTGATATCACACTCCAAGTGCTACGAAC<br>CGCAGCGAGCAGATATCTGGTCCATAGGTATCATATACTGTTGCATGATGTTGAAAAGATTCCC<br>TTGGAAAGTGCCTAAGGAGTCGGATGAGAACTTTAGACTCTACTGTCTGCCCGATGACGAACC<br>TCACGATTATGTGGCCTCTGCCAAGCACCATGAGGAGTTGTTAAATGAACGTAGAGAGAGACA<br>CGAGAGACAGCAAAGAGGTGAAGTTGAACCAGAAGATGAGTGCGAGCATCACCACCATCACCA<br>TCATCACATACACGGGGAAGAACACCATCATCATCAAGAAGCAGAAAAAGCAGAGAAGGAACA<br>ACAGCAAGAACCTGAGAAAACAGAAAAGGAACAGCAGCCTGAAGGTGGAAAGTGTAACCAAGA<br>ATCTCAGGATCCAAAACACGAACAGATGCAGGAAGCTGAGGGTACGAAAAAAGAGCAAGGATC<br>AGATGACACAAAAGGTGCACAGCAGCACGATGAGCAGCATTCACAGCAGGTCTCTCAGCAAGC<br>AGATCAGCAATCTCAGATTGATGCGAAATCAGTTGCTTCGCATCCAAAACCCCCCGCCAGTTCC<br>CAGAACTCCCCACACCACAGGAGGATCTTACACGGTCCCTACAGGTTGCTTCGTCTTCTCCCT<br>CATGCAGCCAGACCTATAATGTCTAGAATCTTGGATGTGGACCCAACTAAGAGAGCCACTATGA<br>AAGACATATACGATGACGATTGGTTTGCCGCTATTCAGTTTTGTACCGTTAATGATAAGAAGGAA<br>GTCGTCCGTGCCCCAAACCATCATCATACTCTGGTTAGAGAGGATGCCAACGGTTCGGAGACC<br>TTCAAAGTTTAA

>ZbHSP30 (ZBIST_0459)<br>[KX656026]	ATGGCTCCAGCATTTCAACCTTTGGTAAAAGCAGGTAACCAGGCAGTTGCCATTAACAGACCAC<br>ACGGACTAGACTACCACATCACCAAGCGTGGTTCAGACTGGCTGTGGGCCGCAACGGCATTGT<br>TTGGCTTGGTTGCCTGTATCTATGTGCTTCTTTTCTTTGTCGCTGAGATCAAGAACACCTCCGG<br>ACTAGCCAGGTATTCCCTTGCTGCTCCTTTCCTTATTGCCTTCTTTGAGTTTTTCGCTTACTTCA<br>CTTACGCTTCTAACTTGGGGTGGACTGGTACGCAGGCAGAATTCCACCACGTCACCGTGAGCA<br>GGCCTGTCACCAATGAGAGTCCAGGTGTGCGTCAGGTATTCTATGCCAAGTACATCGCTTGGT<br>TTTTGTCGTGGCCCTTACTGCTCTTCCTTCAGGAGCTAGCTGCTGCCTCCACGAGTACTTCCAT<br>TCAGCTGGAATCAGTATCAGTGTTGAACATGATCCACAGTTTGTTGGTGCAGATTGTTGGTACG<br>TTCTTCTGGGTCATTGCTCTGCTAGTCGGTGCTCTTATCCCCTCTACCTACAGATGGGGTTACT<br>GGACAATCGGTACCTTTATTATGCTTGTCACCCAAGGCATCATCTTACAAAGACAGTTCCAGGC<br>TCTTCATGCCCGTGGTTTCGCAGTGTGCGTTCTTCTCTTTGTTAACTTGATAGTTTGGTTGTACT<br>TCATTTGTTGGGGTTTGTCTGAGGGTGGTAACAGGATTCAACCTGACTCCGAGGCCATCTTCTA<br>CGGTATTTTGGATTTGTGCGTATTCGCCATCTACCCATCCTTCCTAGTATTCCTCATTGGCCAGT<br>TTGGTAACTGGCCAAACTTCTCTTTCAGAGGCGGCGCAAAGAGCTACAATGAGGGACCCGTTG<br>CCACCAGTGGTACCACCGCTGCTGCTAACCCTGAGATTACAGCTCACAGAGCTAGTACTAGTG<br>ATGAGGTTCATTACGAGAAGACTGAAGGCCCTAACTCTATAAGGGACTCCGGTGAGACCCAAG<br>TACCCAGATCTTCTGCAGCGCACAACAGAGGATCGGAAACAGAATCTGAGTCTGAGTCTGTTT<br>GA
>ZbTPO3 (ZBIST_0758)<br>[KX656027]	ATGTCCACTCGCAACAGCGGAGAGTCTAGTTTACAATCGGATTCAAGCTCGCTGCGCAGC<br>TCGCAGTCATCAACGAGCAACCGCAGCGAGAAGCCCCAGCAGCTGCTGGATCGGCCCAGC<br>AGCGAGATCAGCAGGCGCCAAACGATAGGCGCCAGCATAGCGCTCACACGGACAGAGACA<br>GCACAAACATTGCAGGAGCTGGGTGTGCGTAAGAGCGCCCCTGTGCCCGATGTGGTGGCG<br>CCTACGTATGCGGCTCCCCCAGTTTTCCCCGAGGAGTACACTATGGAGACGCCCTCTGGT<br>GTGGTGCCGCTGACACAGCTGGAGACGCTGGGCAGGCAGGCCACCAACCTGTCGCGGCGC<br>ACCACGGAGCGTCCCCCTGTGCTGGAGAACCTGGAGGAAGTTGAGACAGCCGCGGAGAAG<br>GAGGAGGACATGAGCCCCCATTTTGTGACATTTGTGACCAATGACCCGGAAAACCCTCAC<br>AACTGGGCGCCCATGTGGCGCTGGACCTACACAGTGCTGCTTTCGACGCTGGTGATCTGC<br>GTGGCATACGGTTCGGCATGCGTGGCTGGTGGTCTGGGGACGATTCAGAAGAAGTATCAC<br>GTGGGCCAGGAGGTAGCGATCTTATCGGTGTCTCTGATGGTCATTGGGTTTGCGCTGGGA<br>CCATTGATCTGGTCTCCATTCAGTGACCTGTACGGGCGCCGTGCTACCTACTTTGTGTCA<br>ATGGGGCTGTATGTGATCTTTAACATACCCTGTGCCCTTTCTCCCAACATTTCCGGCCAC<br>ATGGTGTGTCGGTTCTTGTGCGGTGTGTTTGCTTCGTCTGGGCTGTGCATGGTCGGTGCC<br>TCTATCTCAGACATGTTCCCCGCAGCCACCAGGGGTAAGGCTATCGCGTTTTTTGCGTTT<br>GCGCCTTACTGTGGACCCGTGTTCGGGCCCCTCGTCAACGGGTTTGTGTCCGTGTGTACG<br>GGCCGTATGGACTACATCATTTGGTTCAACATGGCATTTGCCGGGGTCATGTGGATCATC<br>GTCTCGGCAATCCCCGAGACGTACGCACCAGTGATCTTGGCCAGGAGGGCCAAGCGGATG<br>AGGAAGGAGACCGGGGACCAGCGTCTAATGACAGAGGCCGAGGCCCAGGGTGTCAGCTTC<br>AAAGACATGATGCGTGCGTGTCTGTTGAGGCCGCTGTACTTTGCAGCGACAGAGCCTGTG<br>CTGGACTTGACGTGCGGTTACGTGTGTCTGATCTATTCTTTGCTTTACGGGTTTTTCTTT<br>GCATATCCTGTTATCTTTGACGAGCTTTACGGCTACAAGGACAATCTAATCGGTCTTATG<br>TTCATCCCCATTTTGATCGGAGCGTGCATGGCGCTGGGAACGACTTTTTGGTGCGAGGCC<br>ATGTACCTGAGGACTATTGCGAAGCGGAAACCGACACCAGAGGACCGTCTATTGGGTGCT<br>ATGGTTGGCGCGCCTTTTGCTGCCATTGCTTTGTGGATGCTGGGCGCCACGTCTTACAAG<br>CACCTGATCTGGGTTGCACCTGCATCCAGTGGTCTCGCTTTCGGATTCGGTATGGTGCTC<br>ATTTACTATTCGCTAAACAACTACATCATTGACTGTTATGCCATGTACGCCTCCAGTGCG<br>CTTTCTACTAAGGTGTTTTTACGTTCTGCAGGCGGTGCGGCTTTCCCCTTGTTCACCACA<br>CAGATGTACCATAACCTGGGGCTGCAGTGGGCTTCGTGGCTTTTGGCGTTTATCGCTTCG<br>GGTATGATTATCATTCCGTTTGCCTTCAAGCGTTGGGGTCCCTTTTTGAGAGCTCATCTG<br>GGCAAGGGCGACTACTCGATGGATGCGGTGAACAGGGCGGCGGAAGAGAAGATGGAAGAG<br>AAGCGCGCTCATGAGGCAGGCGAGGAGATGGCGGAAGAAGTAGCCAATGACGTGTAA
>ZbMSN4 (ZBIST_4795)<br>[KX656028]	ATGAGTGATCTCTTCATGGAGTTGGACACTGCTCTTCCCGAAGTACTGTTGAAAGACCCA<br>CAGGACCATCTCGGGGTGGACCTGAATCCAGACTCTCGGAACGACACAGCAACGTCCCTG<br>TCACTGGATTTCAGTAAGGAGGGCGGCTCCTCCGCTGGTGGTAGCGGTAGCGGTTCTGGT<br>AGCGTGTCTGTCGGTTACGGCAGGCACCACAACTGTGCAGACGCTTCCCCGTGGGAGAGT<br>CCTCAAGAAACCGCACCGGAGGCTACTGGAAATGCTACCGGAATCACCGTGGCCAGTGGG<br>AACAGCGGAACTACTGGTACCACAGCTACTGCCAATACCACTACGAGCGCCACTACTAAT<br>ACAGCAAATACGAACACGGACACGAACACGACCCAAAACGCAGGGTTCATTGACCCCAAC<br>CTGCTGAGCAACAACCTGTTCATGGACACGTTTGGGCTCTCGCCGTCGAACGAGGACTTG<br>AACGGGCCGGACCCGTATGCGCAGCCCAACCTGAACTCGCTGCTGGACGACTACGTTTCT<br>ACGGAGATGATTCTCAACGGGCAAGGTGCCACCCAGGATGTAATTACGACACCTCTGCCG<br>GAGATGACAGGCTCTAGGGGCTCCATTTCTCACAATGTTGACTTCTGGAATTTGACAGAA<br>CAGAGTGCAAACACTCAGCATCTCGAAGACCAAATCTCACCTAGAGCTTTGAGGAGACCT<br>ACCACGCCCTTCACGGGGATTGACAGAGAGCTCGCAGAGGTGCTCAACGGCTATCATATC<br>AACTTCAGACGCAACTCGCTAACCGCCAATGTGCCACAGGCAACACAGCGACACGGATTC<br>AAGAAACAGCCCCAGCGCAGCTCCATGTCAGTGCTGGATGGATCACTGAACACCGACCTC<br>TTCAACAAACTTTACGAAAATGGCGTTCCCTCCTCAAATCTCAAAGTTCTGCCTTCTTGG<br>GACCGTAACGGGTCTTCTGAAGAAGAGGAGGAGGACGTAGACCCTTCCACAGCTGCCGAC<br>ATGCCGGGGCTCGAACCTATAGTGTCCCCCAAGTCACCGAAGTCCGTGTCGGGGGGCCAA<br>GCAAAGAATACACAGCGTTTCATCAATCCATCGATGGTACTCTCTGACAACGCCTCTGCT<br>TCCGCTAGGGCGGCCACCACGGGTACTGAAAAGATAGACGTGGCGCCCAAACGCTACGAT<br>ATCGCCCTAAATCCCCCACCAAATTACCAATCGACAATACCCGAAATTTCCACGGTTCCC<br>ACGGTTCCCACGGTTCCCACGGTTCCCACGGTTCCCACGGTTTCCACGGTTTCCACGGTT<br>CCCACGGTTCCCACGGTCCCGAGAGTTCCCGCGGCCCACTCTGCCTCAACTGGATCTACG

	GGATCCAATGGGCCTACGTTGGAGCAGCAACAGCAGCAGCAGCTGCAGATGCACGCTCCT<br>ACGAAACGAAGGCGCTCTACTGCTAATATTCTGACCATTAGTAGCAACAATATTCAGAAT<br>GGCGTGACCGTGACACCGGCGCAGGCACCAGCAACGAACAGTAGTGGCAGCAACAGCAGA<br>TCCATAACGCCGATGAGCGGGTCCGATGACGACGCTAAACCCTTCAAGTGTAGAGAGTGC<br>TCCAAGGCATTCCGCCGCAGCGAGCACCTCAAAAGACACATACGATCCGTGCACTCTAGC<br>GAAAGACCTTTTGCGTGTATGTTCTGCGAAAAGAAGTTCAGCAGAAGCGACAACTTGTCC<br>CAGCATCTTAAGACGCACAAGAAGCACGGCGATTTTTAA
>ZbYGP1 (ZBIST_0509)<br>[KX656029]	ATGAAGTTGTCAAACGTCGCTTTATTCATGGGTGCGTGCTCTTCCGCAGTGGCCAACCCA<br>GTCCACGAACTTCTGTATAGACGTGACGTGGCTAATGGAACTACTGTAGCTGGCGGCTCT<br>AACTCTTCAGCGGCTGGCGATGCCTCTAACGGCACTGCTACTGCATCGAGTACTTCCCCA<br>AGCAGTACTGCTGCCACGAATGTGACTGGAGGCGGTTACGGCAACTCGAGCTCAGGTTCA<br>GGCTCTAGTTCGGGTTCCAGCGGCTCGGTTAGCGTGTTTGTGACTGGCGGTACTAGCACG<br>TTATCCAACTCTAGCGGGGTGGACGTCAATTGGCTATTCAACAGCTCGCAGTCGCTAAAC<br>GTAACGCAGTTATACCAGATTTCCATCCAGATTAATCAAACACTAGGGCAGGGCTCTAAC<br>AACGGTGTGGTGATAGTTTCTAACGCGGAGTCTCTAGAATCCCTTGGATTCTTTTCTTCG<br>CTCGTCTTCAACACAGACAAGCCGATAGTCATTGCACAAAATGCGGCGACCGGTGCTGCA<br>ATCGCCCAAGACCCATCGTCCAAGGCACGTGGTCCTCTGGTGCTCGGCGACAACCACCTG<br>ATATACCCCGGTGTGTTTGCCCCTTCTGCGGGTGAAACTTCTTCCTGTGCCGCCGTTGGT<br>ATCGCCTCTGATGCCAGCAACGCTACCTGGTTCTTTGAGAACTCTGTACCAGCGTTGACT<br>GGGCCATCTTCCGTCATCAAGCAAAACTACACAAACTTTACTAACATAGACGTTCAGAAT<br>ACACCGGTGGTACCTATTATCTACGATGGTGGCTACTCCACTCAGATCATCTCTTCATTG<br>ACCTCCGCCTCTGGATTTGTCATTGTAAGTTCTGGTGTGAATTCTACTACATCTAGCATT<br>GGCAATACTTCCGTTCCAGTGGTCTTCGCTGAAGGTGGTTCTGGTCTACACTTCGTTGGC<br>GACGAAGACGTGCCTCAAGGTACTATTCCAGCCGGATACTTGTCCCCAGTTAAGGCTCAA<br>ATTCTACTGTCCGTCGCTGCTGTTAACGGTGTCAGCAGCTCCGATGCTTTGCGCTCTCTA<br>TTCCCATGA
>ZbYRO2 (ZBIST_0953)<br>[KX656030]	ATGAGTGAGTTTGTCGACCTTTACAAAAGAGGTGGTAACCAAGCCATCGTGATTAACAAA<br>CCGTACAATACGGATATCCACATTACGGACCGTGGTTCTGACTGGTTATGGTCTGCTATG<br>TGTGTTTTCATCGCGTTGGCGATGATTTGTGTGTTTTTCATGTTCCGCAAACCCGCTACC<br>GAACGTCTATTTTACTACACAGCATTCGCTCCACTAGTGTTCATGGCACTCGACTATTTC<br>ACTCTAGCTTCTAACTTGGGATGGATTCCCGTTAAGGTCAAGTACAACCACGCCAGGACT<br>GCCTCCGAGCACGGTCACCACGGTACCAGGCAGGTCTTCTACGCTCGTTACATCGGCTGG<br>TTCATGTCTTGGCCATGGCCAATTGTCCAGGCCTCGCTACTGGGCAACACTCCTATGTGG<br>CAGATTGCCTTCAATGTTGGTTTGGCTAACACGTATGTGGTAGGTATGTTGATTGCTGCT<br>GTCGTGCACACCACTTACAAATGGGGTTACTACGTGTTTGCCATTGCTGCCGGTATCATT<br>ACCTGTATTTCGGTGATGACCACGACGCGTAACTTGTCGAAGAACATTGGACCCGACGTG<br>CTTGCTAATTTCCAGCGCTACTTCTTTGTCGTTATGCTAATGTGGTTCATCTACCCACTA<br>TGTTTCGGTCTTTCCGAGGGTGGTAACGTGTTACAGCCAGACTCTGAGGCTGTCTTTTAC<br>GGTGTTCTTGATGTGATTTACTTGGGTATTTTGCCTCTGCTATTTGTGCCTTTTGCTTCT<br>CATATTGGTATTGAGAGATTGGGCCTAAACTACACTGTTAACCACTACGGTCAGGCTCCA<br>ACTGGTGCCCAGGGTGGTGCACAGCACGCTGCCCCAACGCCACGTCCACCTGCTCCTACG<br>CCAGCCAAAAAGGCCCCAGCTGCTGGAACCAAGAAGAAGACGAAGAAGGTGAAGAGGGTC<br>AAGAAGGTCACCAAGAAGAAGCACGCGCCAAGCGAGGAAGAGCATTCAAGTGAGAAGGTG<br>AGCGAGCCACTAGAAGAAGAATCTGCTGGTTACTCTAGTTAG
>ZbCRS5 (ZBIST_3713)<br>[KX656031]	ATGTTGAGTGAGATTGTTAACTACGGATGCGAAGCATGCAAAGACGCCTGCCAATGTGGC<br>AGTAAATGCGGCGCTAGCTGTACTGGTAAGGGATGCAACTGCAGCAAGCCTAAATGTAGC<br>AGCTGCGGTGATCACTGCTCATGCACTAGCGATAAGACTTGCGGTTGTAAGCACTGA
