# Supplementary material for: The Zygosaccharomyces bailii transcription factor Haa1 is required for acetic acid and copper stress responses suggesting subfunctionalization of the ancestral bifunctional protein Haa1/Cup2
Source: BMC Genomics. 2017 Jan 13;18:75. doi: 10.1186/s12864-016-3443-2 (PMC5234253; doi:10.1186/s12864-016-3443-2)
Supplement: Additional file 3: — Amino acid sequences of the Haa1 and Cup2 homologues identified in the 28 Saccharomycetaceae yeast species. (PDF 60 kb) [file 12864_2016_3443_MOESM3_ESM.pdf]

Additional file 3. Amino acid sequences of the Haa1 and Cup2 homologues identified in the 28 Saccharomycetaceae yeast species

ORF	Translated ORF
teph_1_**d00690**	MVLVNGIKYACERCIRGHRVTTCTHTDQPLMMIKPKGRPSTTCDLCKELRKNKRIVDKLLEEGKQCT<br>CGRLRKRLEQQKLKDEAKARSKELKRRNKLLGGGEQGNKSNVKIDLLKQHNDNTFDTTNYKRKDSV<br>ISERRMSALSMNSFTGTGKSISMLNNNPHLFGTDSSVHSYSDVSSMNSPTSIDGGNSFINGFSSSNP<br>NNFISNNSNLFTKNYSDSHSNLFNVFNSNNYLDSNVGNNNNNNNTSPQNNDSVHAISSPVNTSGKIT<br>KDYHRVNSLASISSTQSQHYLEQSFSSPQSPSFYNTTHITSNLTANTTNSNGKKDISISSIIPTNLSNM<br>SPDWLSGVNNSENNDNNNMKNSKSNDYPNNNIENNLNDIKIKQEHDLDDMNGLLDSIMNSSALQGM<br>SRASYLLKQENPSNHSLNNPLIKPEPKDDNILDFDVTTANKFTATNTNETNNNATLDKITNAQKNLNN<br>MKNITTNVNDGVGILSLTPGLLDMDANNNFSGANSLLNIPGNDNDFNASSFPDSQLLKNDQDQSLQE<br>PKIKNENYVMGLDPQDEDFAKHSDSIYSSYNDILKGMVNDMSSSNNYSNVNMDNSTIDVSMNSPSN<br>QFGNENLLKDKIFLNNTMNSDLDQQVIQNVIKSEYDDTFNI
teph_1_h00580	MIIDGKKYACQKCIRGHRVTTCNHSGVELVLIKPKGRPSTTCEHCKAVRHNTNFNPMGKCQCGKNV<br>KPIRVRRRSKASISSISNNTTPMVTPASSLMNDCDCLSGSICKCNVTKRKASVVSLTGHGSGFVNSNL<br>NRKSISSYISSPNLVHREDSFSNLFPTESVNNDLSLEFTDNFNKNNREKVDDSGVSLMDFFLNTDKS<br>VDQKIDFNNYPNTSPVSPTTTSFIDNVVGEMNIAMALSNNSSFLNNDDIGLPSDMEYIGKRSSTSESIS<br>PKFVSQPFKNDSNNNNINNSSLNTTQKKHFDTVTNNALVTDNGGTNVSDIFDLNNIFNILESNDVPAT<br>ANDFEFQRNNINWNISNI
naca_1_a05130	MIMLNGEKYACDLCIRGHRSSSCNHRDRQLTKLKPKGRPSTTCMHCKEMRKVKNVNPSGGCPCGK<br>IQNSKNENELKVLKKAAAGCTCLTGGTCRCHIKRRRAKNEDQSPISTTQTTDSSVDNLYDDLISGPILL<br>DDVLTPIFDDASPTMSGSVQQGSIKNLSPIDEFLIDPKTPLTVISDDEQNSDLSSAIKNESSMADIIKQF<br>AESDLQDVNGLTPTDLLQPNGEEVSLLMTKTEPQLNNGDDKIQNPKENEDDLFMDLLDLNNLPEINQ<br>YDYDSLNIAPNLALNNSTPS
naca_2_51_ay00250	MIMLNGEKYACDLCIRGHRSSSCNHRDRQLTKLKPKGRPSTTCMHCKEMRKVKNVNPSGGCPCGK<br>IQNSKNENELKVLKKAAAGCTCLTGGTCRCHIKRRRAKNEDQSPISTTQTTDSSVDNLYDDLISGPILL<br>DDVLTPIFDDASPTMSGSVQQGSIKNLSPIDEFLIDPKTPLTVISDDEQNSDLSSAIKNESSMADIIKQF<br>AESDLQDVNGLTPTDLLQPNGEEVSLLMTKTEPQLNNGDDKIQNPKENEDDLFMDLLDLNNLPEINQ<br>YDYDSLNIAPNLALNNSTPS
nada_1_k02530	MILLDGDKYACDLCIRGHRASTCNHTDRRLTKLRPKGRPSTNCSHCKEIRRISNVNPSGSCSCGKSK<br>KSKPGDKNKIEKDSAIDCGCMSGNPCKCHTNRKDRSTNKRSTTNSPLANNEAVENDDENGRKEEP<br>SQSLIPVLDEILNKEVGDLAVPPELQTTNEITKNEPDLNIHDYPNGRCLSNIQDNDILSPIDEFIADSNLP<br>NTIDPAFLDTGTDGLQLPSLSQPTLAPSTHGSLGVLLDDFYITEPISEFLQDSSKDSNSVFPVHTEKKG<br>FSEKNSNDNHYQRMANPNIMKE
kana_1_e03480	MIIIDKKKYACQFCIRGHRAASCNHSDRPLTEVRKKGRPSTACSHCKEMREAKNINPSGACLCHEAS<br>KSGTDLSPDSEAAMNACLCVTGEPCKCIFKRKRKPKTERKSASKMGVLSRPGDDNILKIDDSFKELV<br>SLLGPGVTTVTAPDTLNQQQLLFQGLNSTSPDIGSVGSVEPQKGAETPHQGGPLNHHLLADDSLTG<br>SIPNFTDTSEDIDRLLAANGLLQETNAGFPSGEIPDVVNSSLLQLDNTDLFSNITEGNNGYEK

cagl_1_i04180g	MVVINGVKYACDSCIKSHKAAQCEHNDRPLKILKPRGRPPTTCDHCKDMRKTKNVNPSGSCNCSKL<br>EKIRQEKGITIEEDMLMSGNMDMCLCVRGEPCRCHARRKRTQKSNKKDNLSINSPTNNSPSPALSV<br>NIGGMVVANDDILKSLGPIQNVDLTAPLDFPPNGIDNKPMESFYTQTSKSDAVDSLEFDHLMNMQMR<br>NDNSLSFPMSANQNEVGYQFNNEGNNSMNSTMKNTITQMDQGNSHSMTLHDIDEILNNGIELGNVN
cagl_2_4_d01770	MVVINGVKYACDSCIKSHKAAQCEHNDRPLKILKPRGRPPTTCDHCKDMRKTKNVNPSGSCNCSKL<br>EKIRQEKGITIEEDMLMSGNMDMCLCVRGEPCRCHARRKRTQKSNKKDNLSINSPTNNSPSPALSV<br>NIGGMVVANDDILKSLGPIQNVDLTAPLDFPPNGIDNKPMESFYTQTSKSDAVDSLEFDHLMNMQMR<br>NDNSLSFPMSANQNEVGYQFNNEGNNSMNSTMKNTITQMDQGNSHSMTLHDIDEILNNGIELGNVN
vapo_1_467.5	MVLIDGVKYACQRCIRGHRVSTCNHVDQPLTVIKPKGRPSTTCNHCKTMRQNHNINPTGKCECGKQ<br>LKSPKNRRRRTKSTKSLNGETSSGLLSPKSIIGNTTITTTTPSINNDNNNNNNNNNNNNNNNNNNYN<br>NNNKRNCDCQTGQICRCHNTRAKKRTSIASPLMENRRDSSTSILSSFSNSNISRDNSGLLDMFKIKD<br>SSISLANSLLNGGFEGEQYDSNVGSRTSSVVNDQVFIPKVEILSLTPDFIDFLNPENLLKNNENINIDTN<br>YLENIQNNDPDLNLMPLTPLDGTIITSTNPNESTETVSPKDDKLIPNDMGIVFNWNFQDLDNLEDIKNS<br>TS
kaaf_1_c03260	MIVINGVKYACELCIRGHRTHSCNHSDRKLIEVRRKGRPSTTCMHCKELRSINKSNPSGKCRCSSTA<br>VKKDISALDCLCTIGETCKCHSKRKRNTKMVNPAPIKYSPLPDSLANSEDKLDDFSSQLVEESLWDIL<br>NEGSPQEELPDETFETDSFLQALNRTETSHKEMDQMSTTSALDSYIEESPQFSRSNESSLSNIPFDL<br>VPDQQSNFQKQNVNISLINLQEDSIKGVRDIPEVTIEDRGSFSNFKGSDLRKTSKSGQDHEF
tebl_1_c00170	MVLINGVKYACERCIRGHRATTCTHTDQRLIMIKPKGRPSTTCKHCKELRINKNVNPSGVCTCSKKDS<br>ANNINNCNCPNGDVCKCHSKRKSKTKEKEKELRERDLNENGVDNVKNEIENEMMPPSDKQKIIKRK<br>RRDCNKIIIRTIIIANNNTSNSNLIPNSSDDQRLNLLDQNTSVGLYPLNNTKKISQNTRTRVGEVTVSLK<br>EYLPYEFGGIGNIDAQQLPVTNTTTKTNTPINMAPDTMTLSTQSDLTALDMDMDIDFNTFNTDIPLNQL<br>KNTISPSNNKNVASQAQSDIEDNISSLNYFNNDTVSLRSSQSNLINNINQNSNSNATPNTNNINSTSN<br>GILDNLLLDFNPVTNHTHQHHYSNNNQSTIGDSESIRSVEVLSLTPSFMDLTDNMLNNNKNTNNSNT<br>NINSSSPNSTSNTNIQNNGLTHFNATLQNHINFVPQTNTNSQIGSTTKIGNSSTLLPQTINSTTLGPSN<br>SQRLMDNLDLNNQMEIRDNLNHSLITNTTFYGADAQTSNTNSSFNITNNNNNTANLNFQQDIPDSLN<br>SILQSSSNTLNNNQNTNLLGVTDTVSNMNDINMLPIIDSNFNSASNVIIKKIMLMNQMLI
vapo_1_541.30	MVLINGVKYACERCIRGHRVTTCTHTDQPLMMIKPKGRPSTTCDLCKELRKNKKALINSPNAICTCGR<br>LEKKRQQQKLKEEAKAKAKALAKEQKREKSRNGLISSSERKKKINSSNASSISNLSNMIKAEGRLSEL<br>SLLSGNGMLPTQHSFTNIHPNSSGYAFSDVSSTNSPPSSIDGRSNYGTNPIAMSRNISESSNFLPNAS<br>SFLDSGHNIASPTSGKITKDYHRVASIASLSSLHSQQSLEQSFSPQSPIHNSLNFVGGSSLNSNTISTN<br>PLYASNNSESHINLSDLTSKQNLQRSKNELSISLDEFLPGDMDNSSPNWLNDTSLNNANIQSLNNSL<br>NQVNQRQNHNNSLIHNNSYNSANSQSLDSGLLDTFMDPSSISGISKATYLLQDNNNKNGTDSKNDIL<br>NYFNETLNIGNNNMSKGQEILQGRGDLNDLSPDSNIVKNELDDLRSLNSVEVISLAPGSLDIPDPSFS<br>STFFDNRQDTQNSLPFNATSKESTNVNNNPVISLSEKQSMHQPTSPDALFSNLNESINYPSQSNSIS<br>YGNLKSNNNFNNTNSPYDTNIQTDLDQILLQTTIPTETDDLLNI

cagl_1_I09339g	MVLINGVKYACERCIRGHRVTTCNHSDQPLMMIKPKGRPSTTCDYCKFLRKNKKTIPDESRCTCGRL EKKRLQKEAEEEARRKGLPLPVPEKKTRKKKEVSSMPPGIISPTSSGATRKKMISLEPRKAISSDHLH LHHSQTSPTMATNGKSLRRRNNTTSSSGLTIGLKEQNSFSSFSGHVGDVTSPASINELSVHPSNMSA NKFYNFSNSLNENDESTNLTSNPFLDRIDSSTSLESSVFNRNIRPNNDSSSLLGRDFLDTIDTGVNNQ QTAIHSAGVSGTSHSSTHTHPVPTGRVTKEYHHIPSMTSISSLHSTQSLEHNFNLPQSPPLSTVSFSL LSDTLSPPTHSGVFRKSNNMSHNNQPIRSLDWESGNQSDNNISPISNNNISQGDRNDYIPNGKNNH NYTASQPRKIQRSVSEQNSHENATIGLNPLYNSKKITPKTRTHVGEVIIPLDEYVPPDINGIGRVNGSE SLTAQDWPLPIDEAPNGVEFNDVMNNRNSDRDNNMANDIQSHYSDNFSTTATEAEQAIMSDSDIITS NPKDLAYTGLLDMLSNGSSVSNMSKMNFFGHGNNKNDRFSVNLNSRDPNNKQNNSIAVSRYSNDYI DDKKVTTDNASTRSVEVLSLTPSFMDIPDRMESNKTEIAADSTNSYNQTRSDSDRRAEFQHFQEQQ MLARQAQSQLYRKKNQTSTQDEPTQSFTSSSKFDGSDPDPILGIRKPSFEDNNMFSNASSHNALAN PKSNSISFDASRESSDYKGVLGKQPSNLMHVREVNNIDDSLLPTFADIDRYSGGDAYMDVDSSLAGV SSREEDSTRANNNQDTGEDFDNKGRFNLTHHTSPTLGRDDNSPISSLQVVSPPSQLLSDEGFAELD NFMTSL
cagl_2_2_b04230	MVLINGVKYACERCIRGHRVTTCNHSDQPLMMIKPKGRPSTTCDYCKFLRKNKKTIPDESRCTCGRL EKKRLQKEAEEEARRKGLPLPVPEKKTRKKKEVSSMPPGIISPTSSGATRKKMISLEPRKAISSDHLH LHHSQTSPTMATNGKSLRRRNNTTSSSGLTIGLKEQNSFSSFSGHVGDVTSPASINELSVHPSNMSA NKFYNFSNSLNENDESTNLTSNPFLDRIDSSTSLESSVFNRNIRPNNDSSSLLGRDFLDTIDTGVNNQ QTAIHSAGVSGTSHSSTHTHPVPTGRVTKEYHHIPSMTSISSLHSTQSLEHNFNLPQSPPLSTVSFSL LSDTLSPPTHSGVFRKSNNMSHNNQPIRSLDWESGNQSDNNISPISNNNISQGDRNDYIPNGKNNH NYTASQPRKIQRSVSEQNSHENATIGLNPLYNSKKITPKTRTHVGEVIIPLDEYVPPDINGIGRVNGSE SLTAQDWPLPIDEAPNGVEFNDVMNNRNSDRDNNMANDIQSHYSDNFSTTATEAEQAIMSDSDIITS NPKDLAYTGLLDMLSNGSSVSNMSKMNFFGHGNNKNDRFSVNLNSRDPNNKQNNSIAVSRYSNDYI DDKKVTTDNASTRSVEVLSLTPSFMDIPDRMESNKTEIAADSTNSYNQTRSDSDRRAEFQHFQEQQ MLARQAQSQLYRKKNQTSTQDEPTQSFTSSSKFDGSDPDPILGIRKPSFEDNNMFSNASSHNALAN PKSNSISFDASRESSDYKGVLGKQPSNLMHVREVNNIDDSLLPTFADIDRYSGGDAYMDVDSSLAGV SSREEDSTRANNNQDTGEDFDNKGRFNLTHHTSPTLGRDDNSPISSLQVVSPPSQLLSDEGFAELD NFMTSL
saba_1_3_c00480	MVVINGVKYACETCIRGHRAAQCTHTDGPLQVIRRKGRPSTTCGHCKELRRTKNFNPSGGCMCASK RRLAVGSEEDESRCRCDEGEPCKCHTKRKTSRKQKGASCHSSASPEAAAVKNIGVLDLEASLGLSS VDLIDLSPSSYVDMTTTLSSLESPLQSDGAKTDDIDDLGLPLLDTLQQSRDPAANLPSANASADTDPI ADTHFNDVDIPFSINELNELYKQVSPHTSHSK
saba_2_521_ta00120	MVVINGVKYACETCIRGHRAAQCTHTDGPLQVIRRKGRPSTTCGHCKELRRTKNFNPSGGCMCASK RRLAVGSEEDESRCRCDEGEPCKCHTKRKTSRKQKGASCHSSASPEAAAVKNIGVLDLEASLGLSS VDLIDLSPSSYVDMTTTLSSLESPLQSDGAKTDDIDDLGLPLLDTLQQSRDPAANLPSANASADTDPI ADTHFNDVDIPFSINELNELYKQVSPHTSHSK
sauv_1_7.97	MVVINGVKYACETCIRGHRAAQCTHTDGPLQVIRRKGRPSTTCGHCKELRRTKNFNPSGGCMCASK RRLAVGSEEDESRCRCDEGEPCKCHTKRKTSRKQKGASCHSSASPEAAAVKNIGVLDLEASLGLSS VDLIDLSPSSYVDMTTTLSSLESPLQSDGAKTDDIDDLGLPLLDTLQQSRDPAANLPSANASADTDPI ADTHFNDVDIPFSINELNELYKQVSPHTSHSK

saar_1_3_c00950	MVVINGVKYSCETCIRGHRAAQCTHTDGPLQMIRRKGRPSTTCGHCKELRRTKNFNPSGGCMCAS<br>ARRLAVGGEEDASRCRCDEGEPCRCHTKRKTSRRQKVGSCHSTESTTDTGCANNLGTLDLEAFLG<br>LNGPSPYVDVTTPTLSSLGSPLQGSDIKSDSTDDLQLPPLSALQQDTGQALDPPSTNEVTNADAFIDT<br>HINDIDIPFSINELNELYKEVSPHTSHSK
saku_1_7.105	MVVINGIKYACETCIRGHRAAQCTHTDGPLQMIRRKGRPSTTCGHCKELRRTKNFNPSGGCMCASA<br>RRLATGSEDDESRCRCDEGEPCRCHTRRKTSRKQKAGSCHSRASPEAASASNLGALDLEAFLGLN<br>DPSSYGNAAVTLSSLKSPVQNDDTNADSIDDLNLSPLGTFEQDPDKLPDPSSANDNVTANATADSPL<br>NDIDIPFSINELNELYKQVSPHT
sami_1_7.105	MVVINGVKYACETCIRGHRAAQCTHTDGPLQIIRRKGRPSTTCGHCKDLRRTKNFNPSGGCMCAST<br>RRAATGSKEDESRCRCDEGEPCRCHTKRKTRRKQKGGTCHGRANHEPANSSGVGALDLEAFLGLT<br>GNTSYVDMTTTLPGLNPPLQGGDTKTDSIDDLELPSLNPLQETPNSSLNPSSANETGGANINVNTSL<br>NDIDIPFSLNELNELYKEVSPHTSHSK
sace_1_ygl166w	MVVINGVKYACETCIRGHRAAQCTHTDGPLQMIRRKGRPSTTCGHCKELRRTKNFNPSGGCMCAS<br>ARRPAVGSKEDETRCRCDEGEPCKCHTKRKSSRKSKGGSCHRRANDEAAHVNGLGIADLDVLLGL<br>NGRSSDVDMTTTLPSLKPPLQNGEIKADSIDNLDLASLDPLEQSPSISMEPVSINETGSAYTTTNTALN<br>DIDIPFSINELNELYKQVSSHNSHSQ
sace_3_3_c00990	MVVINGVKYACETCIRGHRAAQCTHTDGPLQMIRRKGRPSTTCGHCKELRRTKNFNPSGGCMCAS<br>ARRPAVGSKEDETRCRCDEGEPCKCHTKRKSSRKSKGGSCHRRANDEAAHVNGLGIADLDVLLGL<br>NGRSSDVDMTTTLPSLKPPLQNGEIKADSIDNLDLASLDPLEQSPSISMEPVSINETGSAYTTTNTALN<br>DIDIPFSINELNELYKQVSSHNSHSQ
sapa_1_2_b00960	MVVINGVKYACETCIRGHRAAQCTHTDGPLQMIRRKGRPSTTCGHCKELRRTKNFNPSGGCMCAS<br>ARRPAVGSKEDETRCRCDEGEPCKCHTKRKSSRKQKTGSCHSRTNHETANANGLGAADLEAFLGL<br>SGNSSYVDMTTTLPSLKSPPQSGDIKTDSINDLDLPSLDTLEQSPDLPLDPFNIDETEGANSTTGTPL<br>NDIDIPFSINELNELYKQVSPHNSHPK
tode_1_c01350	MVLINNVKYACERCIRGHRVTTCNHTDQPLMMIKPKGRPSTTCDHCKELRKNKNANPSGVCTCGRL<br>EKKRLAQKVKEEARAKAKEERKNQECRCDQNEPCKCHSTRRRSRKISINDTHHFNHSQAGSGHVT<br>SPISVETYNDNYLDSDGGGKISKEYHHIPSLASISSLHSTHSLDQKFNFPQSPTLGNFGGSGTNGQFD<br>NSSICSSARSDSRANLSDMVGSSFEPTKRAPGXRSRVGEVTVPLEEYIPSDINGIGRINDVNAFDDW<br>SPETSATNMNPSVSATYAQSSSNSINHNLHAQDQNRSDSNNSNRNNGLLDMFLDSSTIPAFHDKSN<br>TLPSGMAKRYSFHSPQDAYNHHKENNSFKSNNTTNNITNNKNWESTEGSVDNESVKSVEVLSLTPS<br>FMDIPERRPQHHQLTHRYSQDSQLSQQRSSSVSRNHRYGGSHVSQPAYNMRSPPMTVNPSNVSNI<br>DDNVSLNSLQSPSSSVVEHGLMTNSTDPSLMKNLPDTIRHPRTMNLPERSASTSHFQPSFQWSQPP<br>LPRQGSQTLSTTTDTNSEFDHLMAFDSSINRSDSAQLSTKNDTDKPAYQDGPAPFGNGLARRTLQD<br>TSPASSNQTSSPPSQLLTERGFADLDNFMSIL

zyro_1_f04862g	MVLINGVKYACERCIRGHRVTTCNHTDQPLMMIKPKGRPSTTCDHCKELRKNKNANPSGMCTCGRL<br>EKKRLAQKAKEEARAKAKEEKKLHECRCGSDEPCKCHSNRRSRTSHTRKLKNTNVGGSRPSSQHL<br>DSASIGGMGHVMSPVSMDSNSSIQNGSAGTSNINISGIGKAEPSSLFPSGFLDTGESNGKISKDYHQ<br>VPSLASISSLHSGQSPSFDQKLGLPQSPLLNGLSRNSGGNFFNWADDVGSLYPAKSDSGVNLLEND<br>KPNSNVSGNTNTSSMNNFPGDLGVGIYFGVNNGSTNGNENGNGDGNDNVNGSKSMNTNLKMKNP<br>NAKVPFDEYGVPPEINTNQSNDPSGNNMSAPLNDWNTDKSPDVGNMTNFAQNNGLLDIFMDSTTV<br>AALSKDSLMGQQDNFDFLNENNRTQGKINSNINPSYSHNGTNNHNDINKTNGNFINNGNANHNQYR<br>NRIWASPFGTDRSDTVSVDGESMRSVEGASLAPSFMDTPERASSLHSAHSVLYQQPHPHNQAQKQ<br>IKRTTSANRGYRSQPSGRPAVPITINPSMVSSIDDTISVTSLQSPASSLIDNNGFSTSLGNSGDFGTSN<br>PLFERSKSPQLGLEELTNTTIPTTPQFSKQRSNVIPSTNSKFELDRLLGLDTGNTSGLPDNSAISESNN<br>NDNLVFQNDTATLSSPADKGVFEEASLMNGNQTTSPPSQLFTEKGFADLDNFMSTL
zbist_2620	MVLINGVKYACERCIRGHRVTTCNHTDQPLMMIKPKGRPSTTCNHCKELRKNKNANPSGVCTCGRL<br>EKKRLAQKAKEEARAKAKEEKKLHECRCGYDEPCRCHSSRRRHQSRKVSVNGKGSSSRPVSQQF<br>DANNHVMSPVSMDSSSSMHNVSTSGNGKSTESSGMLASGFLDTEVGNGGKVSKEFHQVPSLASIS<br>SLHSGQSFDQKVNLPQSPLLGGGGSLGFLNGGGGNRNSGNFTNWGDEASLYSVRSDSKVNLTEH<br>SGVLTGMQPPSSSVPKRTSSTKAQLGNVKVPLEEYLSPSDNFNSKINETSSPMQDWYFERTPNEDP<br>NISDQLLGVDGSGGDAGASLQAAQNSGLLDMFMDSSTIPILSKSSLLMQDKLGPAVGNNTNNCTTSP<br>SNGPKGRTWSSPMAADKSDTLSVDGESVRSVEALSLIPSYMDIPDRAPSLHNVQSVLHSHQHQPGH<br>FNQQKQGRRSGSVSRSHRPPPPTSRSGVPVTINPSMVSSIDDTISVNSLQSPTGSVLDNHSLSTPLS<br>NGADYGSCRPERVRSPLLGLSENNPTSPQFYQQQPQVLQSATGPSDSELDQLLGFDTGNNSGIMD<br>NTNYSDKNYGNFNPSANINLNGNRNGNGNVSSNSNGSRNGSGSGNGNSNGNLLFQNDMNTLSSP<br>AMKDVLQDTSPMSSNQTVSPPSQLLTEKGFADLDDFMSTL
zyba_3_9_i00670	MVLINGVKYACERCIRGHRVTTCNHTDQPLMMIKPKGRPSTTCNHCKELRKNKNANPSGVCTCGRL<br>VKKRLAQKAKEEARAKAKEEKKLHECRCGYDEPCRCHSSRRRHQSRKVSVNGKGSSSRPVSQQF<br>DANNHVMSPVSMDSSSSMHNISTSGNGKSTESSGMLASGFLDTEVGNGGKVSKEFHQVPSLASISS<br>LHSGQSFDQKVNLPQSPLLGGGGSLGFLNGGGGNRNSGNFTNWGDEASLYSVRSDSKVNLTEHS<br>GVLTGMQPPSSSVPKRTSSTKAQLGNVKVPLEEYLSPSDNFNSKINETSSPMQDWYFERTPNEDPN<br>ISDQLLGVDGSGGDAGASLQAAQNSGLLDMFMDSSTIPILSKSSLLMQDKLGPAVGNNTNNCTTSPS<br>NGPKGRTWSSPMAADKSDTLSVDGESVRSVEALSLIPSYMDIPDRAPSLHNVQSVLHSHQHQPGHF<br>NQQKQGRRSGSVSRSHRPPPPTSRSGVPVTINPSMVSSIDDTISVNSLQSPTGSVLDNRSLSTPLSN<br>GADYGSCRPERVRSPLLGLSENNPTSPQFYQQQPQVLQSATGPSDSELDQLLGFDTGNNSGIMDN<br>TNYSDKNYGNFNPSANINLNGNRNGNGNVSSNSNGSRNGSGSGNGNSNGNLLFQNDMNTLSSPA<br>MKDVLQDTSPMSSNQTVSPPSQLLTEKGFADLDDFMSTL
naca_1_b05730	MVLINGVKYACERCIRGHRVTTCNHTDQPLMMIKPKGRPSTTCSYCKEIRKNKNAHPPGHCTCGRM<br>EKRRLAQKAKEEARAQAKLQNHDSMNADHSNNTNNNNNNNNNNNNNNNNNGQSALAAELNRIHP<br>SHSSLSDSLIMDLNNNNNNNHRIKSSRVSQRNRPAFDRVTSSTSLDSNYFANQGGSDTSSILNNTFL<br>DADMGNSSGKISKDYHHVPSLASISSLHSTQSLSLDQQHKNNNHHNFQLPQSPPVSNLTFNFVTGK<br>PNNNWDNASIKSSNSIKAHHQQQQSLKRDNSMVQSSASSRVGEVVVPLEEYVPSDINGIGKITDKSS<br>LLDDWAFDDSSNNMDSNPSNGNYKSNFSNNATAATTSTTVNFNEADESTLLAFNNNNNTNTNTNN<br>NNNNNYNDNGTGGEAGLLDAFSDPSTISTLSRANLLLQEKNSDHNPHQSNSFRTQRDSNITSQQQY<br>KQNTTRSRSFRLPNNNNNSSPQHQQQASSSIMNNNNNNIRSVEVLSITPSFMDIPTRQSNDNLNHNLS<br>SDSLSSKQRSFSIDRNHRYFTPSSFMNKASSPTTINPSIVSNIDDQISLNSIQSFPTSGINDVLEVSSNN<br>NDYSIGNQNQTYQNQSQNNQDQFNTNLMMNTNDVDNIAMPQMSQFVTTPSNEGIMNDEIVDQQLL<br>PQQQQPQQPQQQQQQQSQRINRRKANSNGTNFNPSLTLQGPHNNEVPNSSPLSSIQTTSPPSQLL<br>TDQGFADLDNFMSSL

naca_2_9_i00230	MVLINGVKYACERCIRGHRVTTCNHTDQPLMMIKPKGRPSTTCSYCKEIRKNKNAHPPGHCTCGRM<br>EKRRLAQKAKEEARAQAKLQNHDSMNADHSNNTNNNNNNNNNNNNNNNNNNNGQSALAAELNRIHP<br>SHSSLSDSLIMDLNNNNNNNHRIKSSRVSQRNRPAFDRVTSSTSLDSNYFANQGGSDTSSILNNTFL<br>DADMGNSSGKISKDYHHVPSLASISSLHSTQSLSLDQQHKNNNHHNFQLPQSPPVSNLTFNFVTGK<br>PNNNWDNASIKSSNSIKAHHQQQQSLKRDNSMVQSSASSRVGEVVVPLEEYVPSDINGIGKITDKSS<br>LLDDWAFDDSSNNMDSNPSNGNYKSNFSNNATAATTSTTVNFNEADESTLLAFNNNNNTNTNTNN<br>NNNNNYNDNGTGGEAGLLDAFSDPSTISTLSRANLLLQEKNSDHNPHQSNSFRTQRDSNITSQQQY<br>KQNTTRSRSFRLPNNNNNSSPQHQQQASSSIMNNNNNIRSVEVLSITPSFMDIPTRQSNDNLNHNLS<br>SDSLSSKQRSFSIDRNHRYFTPSSFMNKASSPTTINPSIVSNIDDQISLNSIQSFPTSGINDVLEVSSNN<br>NDYSIGNQNQTYQNQSQNNQDQFNTNLMMNTNDVDNIAMPQMSQFVTTPSNEGIMNDEIVDQQLL<br>PQQQQPQQPQQQQQQQSQRINRRKANSNGTNFNPSLTLQGPHNNEVPNSSPLSSIQTTSPPSQLL<br>TDQGFADLDNFMSSL
nada_1_b03070	MVLINGVKYACERCIRGHRVTTCNHTDQPLMMIKPKGRPSTTCAYCKELRKNKNGNIPGSCTCGRQ<br>EKKRLAQKAKEEARAKAKEQLLSGKCTCNTKPNEVCTYHHTLNATGNKYTRQQKKLMSSSSIDIRTL<br>DTNTPTMEIHQMKNNHSKANRSHARSNRPKFERAPSSASLDANFVLAHGHHYKKNHNGNNNTNNN<br>TGGPLSDNASVISSSYLDSEIGSGKISKDYHHVPSLASLSSLHSQQSLEHNFSLPQSPPLSNLNFNFIT<br>GNSTNNSNHHSRHTTTANNWDNASINSANSSNLIQRVANINTANNIQKFQSTNNNSNSRIINSIDENG<br>NNNNNSNTNNDNATQGRMRAGEVVVPLEEYVPSDINGVGKITDKNLLLEDWDPYSIQCSNLNLNLN<br>RNQYDNVTATTTKTNLNELDTSTLNGLDLNNGNMDTNSSQHGLLDMFPDSSTISTLSRANLLLQERN<br>DGLLSINNSTDPSSNNRDIRKQTRNNINMSREFSNSNYTNNNVLHGNINDNGNGNGIHHSSGTSRSR<br>SWRTQQKNVSPQQAKSNLNMNMNMNTNNNSSALRNVEVLSITPSFMDIPESGGPISPTPSISQMNM<br>ANTNNDENIRHILSSTSLSSSKQRSFSIDRNHRYTSPSALMGGKSTTSPTTINPSIVSTIDDQISLNSIQ<br>SFPNSTVNDMVDVSTNTDYSNTFQSQPSQPQQQSSQQGQYPLQIPLSGFQFDTIPKTDIISPGSNFI<br>QFNESPNNKNNNNNNPNFQNVNNTNLDESGNINFEDTFVFKKDSETAKRTNSNSPLKNSNNNSNN<br>NSDLNTNLNLNPDATNMLRNNEMMILNDINGGIREADLNINNNVDKNLVETNVNGGSNSSPISSIQTA<br>SPPSQLLTEHGFQELDNFMSTL
saba_1_11_k00230	MVLINGIKYACERCIRGHRVTTCNHTDQPLMMIKPKGRPSTTCDFCKQLRKNKNASPEGACTCGRLE<br>KKKLAQKAKEEARAKSKEKQRKQCTCGTDELCKYHAQKRHSRKSASNSQKKGRSISRSQPMFERV<br>LSSTSLDSNMLSGHGGLSDTSSIMTSTFLDSEPSVGKISKDYHHVPSLASISSLQSSQSLDQNFSVPQ<br>SPPLSTMSFNFLTGNVNDTNQDYNSHQHPNLNGSWQDSSASLPAKSDSRFNLMDKNHTNHSAGLD<br>LLSHSKRISPISNTRVGEVSVPLEEYIPSDIDGVGKVTDKSSSVYDWPFDESMERNFSTTATAATDSS<br>KLGINDNASSTNNIINANNNNYDNTTNNGRGIENINNNSNNNNNNNNNNNNNNNGNNCDNYNNNEN<br>NNNNNLIKQEHQSNGLFDMFTDSSSISTLSRANLLLQEKIGSQESPSKIEHFSKVPQFRNQLNSRSKS<br>FIHNPANEYLKTGFGNTSNNDIGKGVEVLSLTPSFMDIPEKEKERERSPSSNYITDIPFTRKPRSSSVD<br>VNHRYPPMGPVNIATSPSALNNTIASNLDDQLSLTSLNSQPSSIANMMIDPSNLAEQSSIHSIPQSINS<br>PRMSKTGSRQDKNLQMKKEERNQLNCIQDFPQLDSASGDMNQMFSPPLKSVNRPATMRENSSSS<br>NFIFQGNGIISTPPARNDLPDTSPMSSIQTASPPSQLLTDQGFADLDNFMSSL
saba_2_351_mm00100	MVLINGIKYACERCIRGHRVTTCNHTDQPLMMIKPKGRPSTTCDFCKQLRKNKNASPEGACTCGRLE<br>KKKLAQKAKEEARAKSKEKQRKQCTCGTDELCKYHAQKRHSRKSASNSQKKGRSISRSQPMFERV<br>LSSTSLDSNMLSGHGGLSDTSSIMTSTFLDSEPSVGKISKDYHHVPSLASISSLQSSQSLDQNFSVPQ<br>SPPLSTMSFNFLTGNVNDTNQDYNSHQHPNLNGSWQDSSASLPAKSDSRFNLMDKNHTNHSAGLD<br>LLSHSKRISPISNTRVGEVSVPLEEYIPSDIDGVGKVTDKSSSVYDWPFDESMERNFSTTATAATDSS<br>KLGINDNASSTNNIINANNNNYDNTTNNGRGIENINNNSNNNNNNNNNNNNNNNGNNCDNYNNNEN<br>NNNNNLIKQEHQSNGLFDMFTDSSSISTLSRANLLLQEKIGSQESPSKIEHFSKVPQFRNQLNSRSKS<br>FIHNPANEYLKTGFGNTSNNDIGKGVEVLSLTPSFMDIPEKEKERERSPSSNYITDIPFTRKPRSSSVD<br>VNHRYPPMGPVNIATSPSALNNTIASNLDDQLSLTSLNSQPSSIANMMIDPSNLAEQSSIHSIPQSINS<br>PRMSKTGSRQDKNLQMKKEERNQLNCIQDFPQLDSASGDMNQMFSPPLKSVNRPATMRENSSSS<br>NFIFQGNGIISTPPARNDLPDTSPMSSIQTASPPSQLLTDQGFADLDNFMSSL

sauv_1_16.328	MVLINGIKYACERCIRGHRVTTCNHTDQPLMMIKPKGRPSTTCDFCKQLRKNKNASPEGACTCGRLE<br>KKKLAQKAKEEARAKSKEKQRKQCTCGTDELCKYHAQKRHSRKSASNSQKKGRSISRSQPMFERV<br>LSSTSLDSNMLSGHGGLSDTSSIMTSTFLDSEPSVGKISKDYHHVPSLASISSLQSSQSLDQNFSVPQ<br>SPPLSTMSFNFLTGNVNDTNQDYNSHQHPNLNGSWQDSSASLPAKSDSRFNLMDKNHTNHSAGLD<br>LLSHSKRISPISNTRVGEVSVPLEEYIPSDIDGVGKVTDKSSSVYDWPFDESMERNFSTTATAATDSS<br>KLGINDNASSTNNIINANNNNYDNTTNNGRGIENINNNSNNNNNNNNNNNNNNNGNNCDNYNNNEN<br>NNNNNLIKQEHQSNGLFDMFTDSSSISTLSRANLLLQEKIGSQESPSKIEHFSKVPQFRNQLNSRSKS<br>FIHNPANEYLKTGFGNTSNNDIGKGVEVLSLTPSFMDIPEKEKERERSPSSNYITDIPFTRKPRSSSVD<br>VNHRYPPMGPVNIATSPSALNNTIASNLDDQLSLTSLNSQPSSIANMMIDPSNLAEQSSIHSIPQSINS<br>PRMSKTGSRQDKNLQMKKEERNQLNCIQDFPQLDSASGDMNQMFSPPLKSVNRPATMRENSSSS<br>NFIFQGNGIISTPPARNDLPDTSPMSSIQTASPPSQLLTDQGFADLDNFMSSL
saar_1_6_f02790	MVLINGIKYACERCIRGHRVTTCNHTDQPLMMIKPKGRPSTTCDYCKQLRKNKNANPEGVCTCGRL<br>EKKKLAQKAKEEARAKAKEKQRKQCTCGTDDVCIYHTQKRHLRKSPSNPQKKGRSISRSQPMFERV<br>LSSTSLDSNLLSGHGCLSDTSSILTSTFLDSEPGTGKISKDYHHVPSLASISSLQSSQSLDQNFSVPQ<br>SPPLSSMSFNFLTGNMTDTNQSHNDHQHSKPNHNWQDSTASLTAKSDSRFNMMDKNNSAGLDLL<br>GHCKRISPISNPRVGEVSVPLEEYIPSDIDGVGKVTDKSSLIYDWPFDENMERNSSTTATAAPVSSKL<br>VNTNKANRILNTNNNNYNNIVNIDNNDSGTNNNNNYNNNHNSNSNHNNNTSSNNNNRDFDNNINNI<br>NNINNINNNNNNNNNNNNEDCNIASSRQEHQGNGLFDMFTDSSSISTLSRANLLLQEKIGSQEGPSK<br>QEHYSKSPQFRHQLTARSRSFIHHPANDYLKNTFGNSNNNDIGKGVEVLSLTPSFMDIPEKERETER<br>SPSSNYIADRPFTRKPRSSSIDVNHRYPPMAPTNITASPGALNNAVASNLDDQLSLTSLNSQPSSIAN<br>MMDPSYLAEQSSIHSVPQSINSPRMSKTGCRQDKCVQAKREERTPLNNTQDFSQLDNIPGDMNQM<br>FSPPLKSVNRPNAMRENSSSSNFIFQGNGIISTPSTRNELPDTSPMSSIQTASPPSQLLTDQGFADLD<br>NFMSSL
saku_1_16.292	MVLINGIKYACERCIRGHRVTTCNHTDQPLMMIKPKGRPSTTCDYCKQLRKNKNANPEGVCSCGRL<br>EKKKIAQKAKEEARAKAKEKQRKQCTCGMEEICKYHAQKRHLRKSPSNSQKKGRSISRSQPMFERV<br>LSSTSLDSNILSGHGGLSDTSSILTSTFLDSEPGAGKISKDYHHVPSLASISSLQSSQSLDQNFSIPQS<br>PPLSSMSFNFLTGNVTDPNQGHNNHQHSKSNNNWQDSSVSLPAKSDSRFASMDKNNSVNLDLLG<br>HSKRISIIANPRVGEVSVPLEEYIPSDIDGVGKVTDKSSAIYDWPFDESIERNFSTTATAATDSSKLDIN<br>DNNNSNNIINANDNNYSNTNNSDNSINNNNNNNNNDSNNNSNNNNNNTNHSNSNNYNYDNNRNCDN<br>GTINNNPSRQEHQGNGLFDMFTDSSSISTLSRANLLLQEKIGSQENSSKQEHYSKNPQFRHQLTARS<br>RSFIHHPANEYLRNTFGTSNNNDIGKGVEVLSLTPSFMDIPEKERETERSPSSNYITDRPFTRKPRSS<br>SIDVNHRYPPMSSANIAASPSALNNTVASNLDDQLSLTSLNSQPSSIANMMMDSSNMAEQSSIHSVP<br>QSINSPRMSKTGNRQDKNVQAKKEERNALNSIQDFSQLENTPGDVNQMFSPPLKSVNRPDAMREN<br>SPSSNFIFQGNGIISTPSARNELPDTSPMSSIQTASPPSQLLTDQGFADLDNFMSSL
sami_1_16.247	MVLINGIKYACERCIRGHRVTTCNHTDQPLMMIKPKGRPSTTCDYCKQLRKNKNANPEGVCTCGRL<br>EKKKLAQKAKEEARAKAKEKQRKQCTCGTDEVCKYHAQKRHVRKSPSSSQKKGRSISRSQPMFER<br>VLSSTSLDSNMLSGHGALSDTSSILTSTFLDSEPGTGKISKDYHHVPSLASISSLQSSQSLDQNFSVP<br>QSPPLSSMSFNFLTGNVTETNHSHSNHQHSKSNSNWQDSSVSLPAKSDTRFTLMEKNNSMGLDLL<br>GHSKRISPISNSRVGEVSVPLEEYIPSDIDGVGRVTDKSSLVYDWPFDESIERNLSTTATAATDSSKF<br>DLNDNNNKTNCNNMNRSNFNNNGNNNNSSNTINNNSNKDSNINNNNCSRQEHQSNGLFDMFTDS<br>SSISTLSRANLLLQEKIGSQEGSIKQEPYSKNPQFRHQLTSRSRSFIHHPANEYLKNTFGNSNSNDIG<br>KGVEVLSLTPSFMDIPEKERETERSPSSNYIADRPFTRKPRSSSIDVNHRYPPMPSTNVATSPSALNN<br>TVASNLDDQLSLTSLNSQPSSIANMMMDPSNLAEQSSIHSVPPSINSPRMSKTGSRQDKNFQAKKEE<br>RNPLNSVQDLSLLENAPGELNQMFSLPLKNMNRPDAMRENSSSSNFIIQGNGTMSTPSARNELPDT<br>SPMSSIQTASPPSQLLTDQGFADLDNFMSSL

sace_1_ypr008w	MVLINGIKYACERCIRGHRVTTCNHTDQPLMMIKPKGRPSTTCDYCKQLRKNKNANPEGVCTCGRL<br>EKKKLAQKAKEEARAKAKEKQRKQCTCGTDEVCKYHAQKRHLRKSPSSSQKKGRSISRSQPMFER<br>VLSSTSLDSNMLSGHGALSDTSSILTSTFLDSEPGVGKISKDYHHVPSLASISSLQSSQSLDQNFSIPQ<br>SPPLSSMSFNFLTGNINETNQNHSNHQHSKSGNNWQDSSVSLPAKADSRLNMMDKNNSVGLDLLG<br>HSKRISPISNSRVGEVSVPLEEYIPSDIDGVGRVTDKSSLVYDWPFDESIERNFSTTATAATGESKFDI<br>NDNCNRINSKSYSKTNSMNGNGMNNSNNNNINSNGNDKNNNNSSRQEHQGNGLFDMFTDSSSIST<br>LSRANLLLQEKIGSQENSVKQENYSKNPQLRHQLTSRSRSFIHHPANEYLKNTFGNSHSNDIGKGVE<br>VLSLTPSFMDIPEKERETERSPSSNYITDRPFTRKPRSSSIDVNHRYPPMAPTTVATSPGALNNAVAS<br>NLDDQLSLTSLNSQPSSIANMMMDPSNLAEQSSIHSVPQSINSPRMPKTGSRQDKNIHTKKEERNPL<br>NNIHDLSQLENVPDEMNQMFSPPLKSMNRPDAIRENSSSSNFIIQGNSMISTPSGRNDLPDTSPMSSI<br>QTASPPSQLLTDQGFADLDNFMSSL
sace_3_5_e02840	MVLINGIKYACERCIRGHRVTTCNHTDQPLMMIKPKGRPSTTCDYCKQLRKNKNANPEGVCTCGRL<br>EKKKLAQKAKEEARAKAKEKQRKQCTCGTDEVCKYHAQKRHLRKSPSSSQKKGRSISRSQPMFER<br>VLSSTSLDSNMLSGHGALSDTSSILTSTFLDSEPGVGKISKDYHHVPSLASISSLQSSQSLDQNFSIPQ<br>SPPLSSMSFNFLTGNINETNQNHSNHQHSKSGNNWQDSSVSLPAKADSRLNMMDKNNSVGLDLLG<br>HSKRISPISNSRVGEVSVPLEEYIPSDIDGVGRVTDKSSLVYDWPFDESIERNFSTTATAATGESKFDI<br>NDNCNRINSKSYSKTNSMNGNGMNNSNNNNINSNGNDKNNNNSSRQEHQGNGLFDMFTDSSSIST<br>LSRANLLLQEKIGSQENSVKQENYSKNPQLRHQLTSRSRSFIHHPANEYLKNTFGNSHSNDIGKGVE<br>VLSLTPSFMDIPEKERETERSPSSNYITDRPFTRKPRSSSIDVNHRYPPMAPTTVATSPGALNNAVAS<br>NLDDQLSLTSLNSQPSSIANMMMDPSNLAEQSSIHSVPQSINSPRMPKTGSRQDKNIHTKKEERNPL<br>NNIHDLSQLENVPDEMNQMFSPPLKSMNRPDAIRENSSSSNFIIQGNSMISTPSGRNDLPDTSPMSSI<br>QTASPPSQLLTDQGFADLDNFMSSL
sapa_1_5_e03020	MVLINGIKYACERCIRGHRVTTCNHTDQPLMMIKPKGRPSTTCDYCKQLRKNKNANPEGICTCGRLE<br>KKKLAQKAKEEARAKAKEKQRKQCTCGTDEVCKYHAQKRHLRKSPSNSQKKGRSISRSQPMFERV<br>LSSTSLDSNMLSGHGALSDTSSILTSTFLDSEPGAGKISKDYHHVPSLASISSLQSSQSLDQNFSVPQ<br>SPPLSSMSFNFLTGNITETNQSHSNHQHSKSNNNWQDSSVSLPAKADSRFTMIEKNNSVGLDLLGH<br>SKRISPISNSRVGEVSVPLEEYIPSDIDGVGRVTDKSSLVYDWPFDESIERNFSTTATAATDSTKFEIN<br>DGSNRTKYNNNKGYSNTNNINGNVINNGNDNNSNSNIINSNNNDNNNNNSSRQEHQGNGLFDMFT<br>DSSSISTLSRANLLLQEKIGSQENSVKQEHYSKNPQFRHQLTSRSRSFIHHPANEYLKNTFGNSHGN<br>DIGKGVEVLSLTPSFMDIPEKERETERSPSSNYIADRPFTRKPRSSSIDVNHRYPPMASTTVATSPGA<br>LNNAVTSNLDDQLSLTSLNSQPSSIANMMMDPSNLAEQSSIHSVPQSINSPRMPKTGSRQDKNIHTK<br>KEERYPLNSIQDLSQLENAPDEMNQMFSPPLKGMNRPDAMRENSSSSNFIIQGNSMISTPSARNELP<br>DTSPMSSIQTASPPSQLLTDQGFADLDNFMSSL
kana_1_d02340	MVLINGIKYACERCIRGHRVTTCNHTDQPLMMIKPKGRPSTTCSYCKELRKNKCAKPPGTCTCGRQE<br>KKRQQQRAKEEARAKAKELANQSCTCGTEVPCAVHANKRLSSSTSSRKLSGQTSNPVNIQRKNKS<br>RQQKFERAASTASLDSHFFSNQSVASDSSGYFPSSFIDNDSVPGKISKDYHHVPSLASISSLHSSTSL<br>DQNLASPQSPTLVGPSFGFFSDANHAVSNSFTNSGSLVQRAKHHLNNWDTNSSAGPTRSDSHLNL<br>LENNLSVGLDSLNGARKGSAVLPKRSVGEVIVPLEEFIPSDINGVGNVNDADGWDLEDASIKNLSTTA<br>TTHTNTNTDYYRSAGNAPSRGSEAPAQMNDEHIKHMASNGLLDMFPDSSSISTLSRANLLLQEKNG<br>QTEESAQPTQSFHQWNNVQGDQHRLQQPAHQQQQPQQSVSEYPQSLNDNDSVQSVEVLSLMPS<br>FMDIPDYEPHHKRKQRPARATSSSEGYNNKRSSSIDRNHKYTKNSPGRTINPMVVSNLDDGSSLHS<br>FQSPESHGVGLENFNMSAIDENIAHDINGKNLTGALNHQAGNGGGIDTNGKLQSPDLKPPSSSPTDL<br>LSDQGFAELDSFLSTL
kaaf_1_b01950	MVLRNGIKYACERCIRGHRVTTCNHSDQPLMMIKPKGRPSTTCAYCKELRKNKSAKLQGTCTCGRQ<br>EKRRLAQKAKEDARAKAKELERSNCKCSADTTCTHHGSKPQNKKLRGKFLERATSSTSLDSAFLSS<br>HSIYSDSSNFSSTFLDSDINPGRISKDYHHVASLASISSLQSSQSLDQSLSSPHSPPSHPQFNFLSDF<br>DSLTQSTSQVNLLENVDFTPNSTNKLKEHISSGRSNVGEVLVPLEEYIPPDIDGIGNVNDKTNMLNGF<br>SPNDNSIKDNQNETPPNRTGLDSATIQTNPREAGQIRMHPSHGLFDMFSDVSSISTLSRANLLLQKH<br>NVKNVEPEEGISRDIRHSTDFNGVRSNFAGQMQSYKENSTNNVGEAQSTQSVEVLSITPSFMDIPSC<br>EHIGGRNYSAQCNSVAAKVRSSSIDKNHKYPKNINDANISSMIKEENNEFGGFEANVETENNSRRIVS<br>SSDLQITPGEITGPIVVKEEIPENSTVDTAQSWVLNSPLLSEQGFADLDNFMSTL

lakl_1_b03344g	MVLINGVKYACERCIRGHRVTTCNHTDQPLMMIKPKGRPSTTCNHCKELRKNKNANPSGMCTCGR<br>QEKKRLAQRAKEEARAKAKSETCKCLDKEKCLCHSKRRSRKPSIKGRPKSSGGITAGTNITPAGTPA<br>LAMLDSDFGKISKPHPLTSLTSLHSSQSLDQDFSLAGSPAMSQSLSNNNWDASSISSSLRSESRLNL<br>LERGRVNNNLVGLDPLNDTRPITPHTRARVGEVTVPLEEYIPSDINGIGNVNDNSLLSEEMTWSMNN<br>TGNGLLDLFTDTSSRSGINYTKLKEQLLQQKKAQDDSSGGLDAFSSPENECSNTLGSNSLLLDNRRT<br>NTATTSKLINRSDSIRSNSSHESNQSDANKPPLRSLSSFDSASQCLQNSHKVSHQQVALPHNGNYR<br>RRKSHFGTLQEKSSTDACGLDNESVKSVEVFSLTPSFMDIPENGKSLYMNSASTNPFYHHSNTTLPR<br>QRSASVHRNHRYEQHPTLKHSRTDSSFGGINPTMVSNFDDRISLNSLASPPNVEPNSFSNFPQTLSP<br>KESQFTKKLVGPTRFTSELNQALVIDGIDLEDNSMFSDPANDFGINNVKSNESSSVTNSSRPSPSQS<br>NQVFNENFSGLDNLMSGL
lath_1_g16984g	MVLVNGIKYACERCIRGHRVTTCNHTDQPLMMIKPKGRPSTTCAHCKELRKNKNANPTGGCTCGRQ<br>QKKRLAQKAKEEARAKAKSKDDSCRCIETDKCTCHARRKNRRSVSSRSRTTTTTTSSIASEDSPTMP<br>TCDGPDFLGRIAKSHAMGSLPSFHSSQSLDRDFGLATSPVLSPSLSNYHGNSSNWDANSITSLKSDS<br>RINLSDRSHQSIGLDSLHSTRAFPPQARARVGELPIPMEDYSSDLNSLSANSNFNPSGSSNRVAWSE<br>ADPNHGLLDLFADTSENVNTNYMAMKSSLKRNKSYASGLGVMTPQCAGKDPYAQITRSQSYQRDV<br>PKHLVSRPSVDSVISHSSKVSEGANNGRETLQSNHSLPFITARQLQPRMSYGGNNFPPGCLSLDDD<br>SAKSVEVLSLTPSFMDIPDNTNFYSATSNPFLHQQKVEPRKRSVSIHRNHRYDSFTRGASGESHTPV<br>IQQTQSPHSRQGGEVKAEATTRAESQDADSDSAFPLSQVTPSGPSMNISPGADSNSLTQTSHVSSP<br>RDTLTNQFMEPTSSFTSEIDHILGSNNDDVQSLFSDPTISLDATHSMNLEASPIDQAHRMPSLCYSET<br>TGASNLDDLDKLMAE
lawa_1_14.1543	MVLVNGIKYACERCIRGHRVTTCNHTDQPLMMIKPKGRPSTTCSHCKELRKNKNANPSGECTCGRQ<br>EKKRLAQKAKEEARAKAKSKDDCRCIETDKCTCHARRKNRRPAASKSGKITATHNTASSIKSADSPP<br>VANLDVGDFMGKVAKTHVMGSLPSFHSSQSLDRDFSIAASPTLSPALNSSMHSNAFNWDTNSITSSL<br>RSDSRVNLSDRGHHSFTPELSQSNKALSPSSRARVGEVSIPLEEYSSSDLQGMSGSMDNSGFSNR<br>MGWPETDANHGPLDLFADTSENLNTNYMSLKSNWQRNKGSNCGLNSTHSASELREHPVPLSRSFS<br>TQRDVPKPLVSRTSVDSIISDSSRLSHSVVNGTDLSTTNQSVPYISARQLQPRMSFGGYNLPPGCIDE<br>ENAKSVEVLSLTPSFMDIPDNTNLYSATSNPFLHKQGDIEPRKRSVSIHRNHRYDSFSKPSSWNHKH<br>MPPYHQRQSSHSHLEYEVKREHTPQSCCQDGSSDHNATSNLDSMSAPRMKLSPENESDSITHTSH<br>VSSPRDGLVNQFMEPTSSFTSDIDHILGSNHDDVQSLFSNPTLPLDDTNPLNFDDAPVGQTNTFPVM<br>PS
klae_1_13_m00540	MVLINGVKYACERCIRGHRVTTCNHTDQPLTMIKPKGRPSTTCSHCKELRKSRNANPSGRCTCGRQ<br>EKKRLAQKVKDEASCVCAKDPDNCACHKKRNSRRKKQSESSFTVDRDGKISKNNGHSYQSLHSLA<br>SSQSIDQDISNLLASPISMNTSFSTGWDSVSVSSSNRSPTGNGSKDNKNTIGLDPLSSMKPITPMTRT<br>KVGEVYIPLNEYVPTELTSMENDTEHINSLLMGENVSILEDVANSGSGLTFFADTSKPLSYNTMKEQR<br>RNFADLNLVDSSANVYHKLSRPDAVRSTSFASNMSNMSGHDSSVLPNGNEFLTSLNSSDSLSSFTT<br>AGNSHHYQDMIHHPSKGPNHFNPIQADASSNVYGLDNESVRSVEVLSITPSFMDIPESKASSINSQS<br>SSNSNFISWKSVNSRRERSVSIHKNHRYEDGKRNLHKQSSSSMARKPIRSMVLPEEPEVGTNSAAM<br>LQNHSPSDSVNTLLSGNTQNGDPINLNALTTSPQPFLDSSLAPDFHHALKQSNLLNQTYIDNNSLFSA<br>TSEGNNHSPPDALPVDFADIDDLMTNL
klwi_1_120_dp00120	MVLINGVKYACERCIRGHRVTTCNHTDQPLTMIKPKGRPSTTCSHCKELRKNKNANPSGQCTCGRQ<br>DKKRQAQKAKEEASCSCATDPDHCACHKKRSSRRKKPIDSNLSLDTDGKISKHNGFSYQSLHSLNS<br>TQSLEQDISNLLASPVSMNTSFSTGWDAMSATSSNRSPPANVVENQNTIGLDPLTSMKPITPMTRTK<br>VGEIYIPLNEYVPTDIISKDSNPNRINPLLMGEDLSVYDDQSTNIPNVTYFADTSKPLSYSQLKEQRKTL<br>TEHNIESAKNRTFQRNQPQQAVRSPSFVSNISSHDSVVSNSNDFLSSLNSSDSLSSMMNGGNAHHY<br>QDMLHHSNHGRSLYNPIQPDQSPNVYGLDNESVRSVEVLSITPSFMDIPESNPASIASNSSNPFISW<br>KSVNSRRERSVSMHKNHHYDLENKRKRHPKNLANSGKPPVRSMILPTEPKSEPLLAAFSNINSPTDS<br>INTLLSNTTQNGDPIIMNNSKPSTVPQPFLESSFTTDFNEALKQSNIVNQHLIDNNSLFSGTSEGNNPS<br>PSDQFPVDFADIDDLMTNL

klla_1_a03047g	MVLINGVKYACERCIRGHRVTTCNHTDQPLTMIKPKGRPSTTCAHCKELRKNRNANPSGQCTCGRQ<br>EKKRIAQKAREESGCVCKNDPDHCPCHKKRAPRRKKQSHQGNSAVGLDINGKISKHTNSGYSHQSL<br>HSLDSSHSVDQDLSNFLASPISMNTSFSTGWDNASLSSSNRSPPGNGPDSKNTIGLEPLSTMKPITP<br>KTRTKVGEVFIPLTEYVPTNIASSEDQADHINPLLMGEHVSIYNDVSADAPNVTYFADTSKSLSYNQLK<br>EQRKALSEQHLRNNVDNISKSGSTQRVPLSVAGRSSSFVSNISSHDSVASSNNDFISSLNSSDSLSS<br>MLHSGNAHHYQDMLHHSNNGRGYLNPVQVDQSQNVYGFDTDSVRSVEVLSITPSFMDIPESKPPSI<br>GSNSSTNPFISWKSVNSRRERSVSIHKNHRYDNENKRKRHPKNVPNTSTPQIKSMVSPAETNSTALP<br>ASGNHFNSPADSTNTVLSANTQYGDPSVFSIEKVGVAPSFVEPSFTTDFNQALKQSNMVNQNIIDNN<br>SLFSGTSEGNNPSPNDLFPVEFADIDDLMTHL
klma_1_3_c06150	MVLINGVKYACERCIRGHRVTTCNHTDQPLTMIKPKGRPSTTCAHCKELRKSKNANPSGQCTCGRQ<br>DKKRLAQKVKEEASCTCKTDPDHCACHKKRGAKRKTKGQNSGTSIGLDLDGKVSKSNAYSFQSLPS<br>INSSQSLDKDISNLLGSPISMNTSFSTGWDTGSISSSNRSPPGSGNTYSNISNGNTGTISNNNNNNNN<br>NNNHHHHHKHGQDTSKNTVGLEPLSIMKPITPNTRTKVGEVYIPLTEYVPTSITSSHDQTDSINPLLM<br>GDSLSIYDDGQSDTPNVTYFADTSKSMSYTQLKEQRKSVNNEHVPSQRVPLANTGRSSSFISNISSH<br>DSVISNNDFISSMNSSDSLSSMLQGGNAHHYQDMLHHPSNGRGSGFNPVHIEQSPNVYGFDTDSV<br>RSVEVLSITPSFMDIPESKQASAESNTSSSGYISWKGVNSRRERSVSIHKNHRYDSENKRKRHPLTS<br>ANSSKQQIKSMILPIEENNNHNSSSSNENTAATLPTTSNNFNSPADSTNTALSSNAAFGDQSVFSTER<br>RGLEPSFVDPQFSPDFNPSLKQSHMINQNIIDNNSLFSGTSEGNNPSPNDIFPVEFADIDDLMTHL
ercy_1_6116	MVLVNGVKYACERCIRGHRVTTCNHTDQPLMMIKPKGRPSTTCAHCKELKKNRNSNPSGVCTCGR<br>EEKRRLLKKAKEEARAKLKEEKCGCHNKAACTCHKLRQRKSSSKRKQIAVSEIEIGGNSLGIDKVPRT<br>VNNNVEGSLKSGQFAGNSKFSLMGSVLSVHSVQSGDTDPLVLVSPAHSGVSNNFHGSHAGNISWD<br>NNSVASSLQSETYLGGSNTPLGLDPLLNINPVTPLARMHMGEVSISLNEDIPSDPGSNIQSTNTLMNL<br>NDRISLQDDGGKEEKLDFFTDTSKAQLNYNELKQKRLRKPLDVTSQDNGVMETTGLVSSNSMRSAS<br>PTEIVSPTASLSTSNSRLRTLNSSDSILSLQTNRGNGYNNAHYYQNMIHYPLNQKSSFSFANSPSVHS<br>VDSVSRQAFPNNNSIAASVVPSAISTSYVARTSSIVPATSGNNQNQGNRVEEMSLNPNFIDLPVNAY<br>KNPYHISNLNNKQASEPQILRDNNQVLLRQQTRQRSVSIHKDHRYDQFSSQEHQFKKTSINTSINST<br>GINPNELSTLENSKSTKSFASSSTESSANTSANPKPCTHKPSMLESTFTAEFNQLLSETTNALDTECIF<br>SANCALWNSDISVSPTAATIRGTTLPQTNPENTTAITGTSQENANNILRFVDANYADLDSLITNL
asac_1_7_g04120	MVLLNGVKYACERCIRGHRVTTCNHTDQPLMMIKPKGRPSTTCAHCKELKKNRNAHPSGVCTCGR<br>EEKRRQLRAAKEEARARGRRCRCADREACVCHKTRRRREDGEAGSASEQSSVDGHVMGSPHEP<br>WDAASAASSLPPELSLESATPLGLEPLHAMKPITPLTRTHVGEVSIPLHEYVPAELDNSVQSANPLLN<br>LNDRICLQDGAGREERLNFFADTSKARPNYSELKQRRLARAADGTYAQSLRSASSTELASPAASGS<br>STSLLHTLNSSDSLSSYLTAGGSAYNSTHYQDMLHHSFSHLGSVPLAPSPQSSSTAATHLTGGDQLP<br>ANNFLDSPDTAYRVPASILHPPTGQAGGAAAHAQRQVAPAMVQPARQRSVSIHRNHRYEKFPTSNS<br>SQPKDSPLGPVANLAAHSFQDLSSVGQSTRQDAFAYSNHNVVANDRQPSFPRNPAPESTFTAEFN<br>QLLSESSNCLELDSIFSGNSVLWNGETLTSEARTNQESDLPSVSEDARDDGQANSAQNAPEILSLAD<br>TDYADLDSLITNL
ergo_1_ael295c	MVLLNGVKYACERCIRGHRVTTCNHTDQPLMMIKPKGRPSTTCAHCKELKKNRNAHPSGVCTCGR<br>EEKRRQLRAAKEEARVRGRRCRCADREACVCHKTRRRRDDGEAGSASEQSSVDGHVMGSPHEP<br>WDAASAASSLPPELSLESATPLGLEPLHAMKPITPLTRTHVGEVSIPLHEYVPAELDNSVQSANPLLN<br>LNDRICLQDGAGREERLSFFADTSKARPNYSELKQRRLARAVDGTYAQSMRSASSTELASPAASAS<br>SASLLHTLNSSDSLSSYLTAGGSAYYSTHYQDMLHHSFSHLGSMPLAPSPQSSATATHLTGGDQPP<br>TSQFLDSPDPAYRHPAYRHPASILHPPTGQAGDATAPAQCQVAPAMVQPVRQRSVSIHRNHRYEKF<br>PTSNPSQPKDSPLGPVAVLAAHSFQDLSSVGQSTRQDAFAYSNHSVVANDRQPSLPRNPAPDSTFT<br>AEFNQLLSESSNCLELDSIFSGNSVLWNGETLTSEARATLEGDVPSVSEDARDDSQANSAQNGPEV<br>LSLADTEYADLDSLITNL
