# Supplementary figures and images for: The Zygosaccharomyces bailii transcription factor Haa1 is required for acetic acid and copper stress responses suggesting subfunctionalization of the ancestral bifunctional protein Haa1/Cup2
Source: BMC Genomics. 2017 Jan 13;18:75. doi: 10.1186/s12864-016-3443-2 (PMC5234253; doi:10.1186/s12864-016-3443-2)

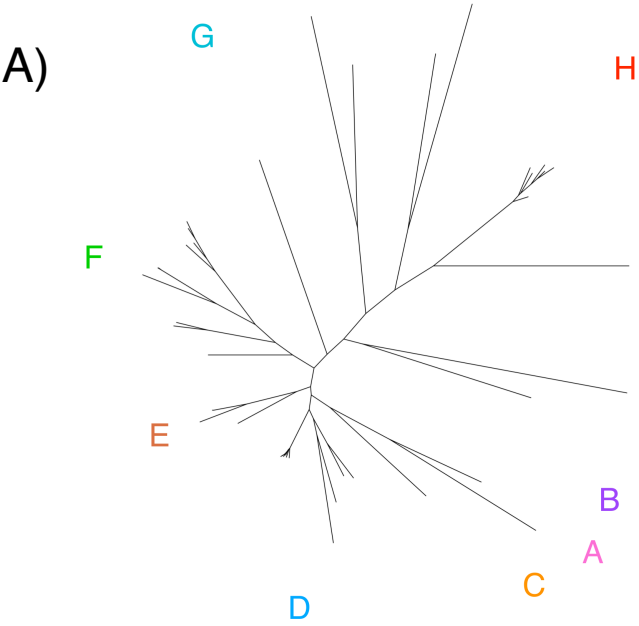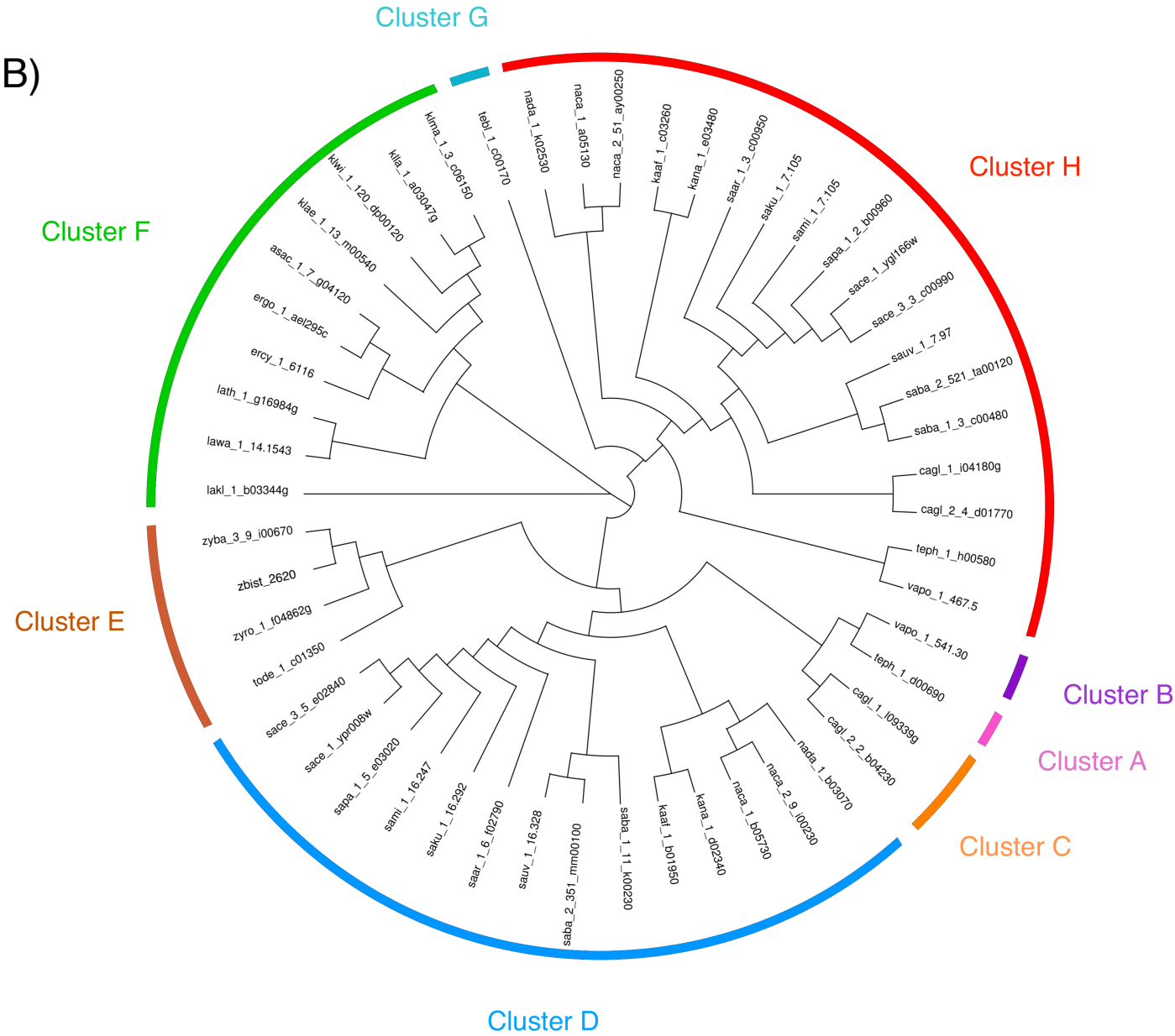

Supplement: Additional file 4: — Phylogenetic analysis of S. cerevisiae Haa1 and Cup2 transcription factors homologues encoded in the genomes of 33 strains of 28 yeast species belonging to the Saccharomycetaceae family. A) Radial phylogram showing the amino acid sequence similarity distances between these 51 full-size proteins. B) Circular cladogram showing the tree topology, with the name of the S. cerevisiae and Z. bailii members indicated. PhyML software suite was used in phylogenetic tree calculation. The gene and species annotation adopted in this study uses the four letters code described in Table 1. (PDF 1465 kb) [file 12864_2016_3443_MOESM4_ESM.pdf]
