# Supplementary material for: The Zygosaccharomyces bailii transcription factor Haa1 is required for acetic acid and copper stress responses suggesting subfunctionalization of the ancestral bifunctional protein Haa1/Cup2
Source: BMC Genomics. 2017 Jan 13;18:75. doi: 10.1186/s12864-016-3443-2 (PMC5234253; doi:10.1186/s12864-016-3443-2)
Supplement: Additional file 5: — Gene neighbourhood analysis of the S. cerevisiae HAA1 and CUP2 orthologues from species of the Saccharomycetaceae family. Central boxes represent S. cerevisiae HAA1 and CUP2 orthologues. Adjacent boxes represent their gene neighbours. Homologous gene neighbours are highlighted using the same colour and identified with the same number. A white box represents genes with no homologous neighbours in the represented chromosome region. (PDF 2076 kb) [file 12864_2016_3443_MOESM5_ESM.pdf]

14877	92846	14878	14879	5640	14881	15983	15	16831	13994	65659	91639	153	15140	92847	sace_1_ygl166w	559	92848	15143	91636	1208	14334	2911	91635	65652	91634	1619	92849	7651	15	13507
2590	153	14877	14878	14879	5640	14881	15983	15	16831	13994	65659	91639	153	15140	sace_3_3_c00990	559	15143	91636	1208	14334	2911	91635	65652	91634	1619	7651	15	13507	13506	2241
153	14877	14878	14879	5640	14881	15983	15	13661	16831	13994	65659	91639	153	15140	sapa_1_2_b00960	559	15143	91636	1208	14334	2911	91635	65652	91634	1619	7651	15	13507	13506	2241
14877	14878	14879	5640	14881	15983	15	129048	129047	16831	13994	65659	91639	153	15140	sami_1_7.105	559	15143	91636	1208	14334	2911	91635	65652	91634	1619	7651	15	13507	13506	2241
94783	153	14877	14878	14879	5640	14881	15983	15	16831	13994	65659	91639	153	15140	saku_1_7.105	559	15143	91636	1208	14334	2911	91635	65652	91634	1619	94807	7651	15	13507	13506
153	14877	14878	14879	5640	14881	15983	15	16831	13994	65659	91639	91638	153	15140	saar_1_3_c00950	559	15143	91636	1208	14334	2911	91635	65652	91634	1619	7651	15	13507	13506	2241
2590	153	14877	14878	14879	5640	14881	15983	15	16831	13994	65659	91639	153	15140	saba_1_3_c00480	559	15143	91636	1208	14334	2911	91635	65652	91634	1619	7651	15	13507	13506	2241
0	0	0	0	0	0	0	0	0	0	0	0	0	0	0	saba_2_521_ta00120	15140	153	0	0	0	0	0	0	0	0	0	0	0	0	0
153	14877	14878	14879	5640	14881	15983	15	16831	13994	65659	95603	91639	153	15140	sauv_1_7.97	559	15143	91636	1208	14334	2911	91635	65652	91634	1619	134805	7651	15	13507	13506
2458	14345	65655	65656	65657	768	65658	12370	12372	5048	12374	65659	65660	153	15140	kaaf_1_c03260	65662	12352	1541	65663	15143	2040	21	12375	7000	13352	112820	65664	65665	9882	13347
13345	13346	13347	9882	66717	66718	112909	13352	559	15143	65659	66719	66720	153	15140	kana_1_e03480	11810	14712	14374	66722	2328	14355	1801	14359	37112	112910	400	15020	11191	7180	66723
1465	266	12396	83501	83502	14706	14705	285	9862	14228	14702	83503	83503	153	15140	naca_1_a05130	181	13359	116329	13358	36829	5596	13914	2496	2991	12399	36832	65916	13360	16852	14712
1465	266	12396	83501	83502	14706	14705	285	9862	14228	14702	83503	83503	153	15140	naca_2_51_ay00250	181	13359	116521	13358	36829	5596	13914	2496	2991	12399	36832	65916	13360	16852	14712
12228	15	1214	85099	14905	14697	14698	37486	14699	83511	14701	85100	15	153	15140	nada_1_k02530	181	13359	65916	36832	12399	2991	2496	13914	5596	36829	13358	116647	85102	5186	14478
4478	12997	12998	2913	13154	12210	1208	13160	109373	559	15143	37041	37040	153	15140	cagl_1_i04180g	37038	37037	14077	14078	13300	2419	37036	14423	109324	3908	225	134	14416	134	37035
4478	12997	12998	2913	13154	12210	1208	13160	109502	559	15143	37041	37040	153	15140	cagl_2_4_d01770	37038	37583	14077	14078	13300	2419	37036	14423	3908	225	134	14416	134	37035	3208
13508	13509	10118	98687	1619	98688	98689	14333	2911	1074	135205	2040	65659	98690	15140	teph_1_h00580	98692	7142	98693	12352	98694	70676	1786	12860	12858	37489	12855	12854	12853	1610	2714
0	0	0	0	0	0	0	0	0	0	0	13995	13994	65659	15140	vapo_1_467.5	100191	7142	100192	12352	2714	1610	14667	14668	14669	14670	5571	14783	14672	14673	6124
12382	2390	12383	12384	375	9414	12385	5932	12388	119796	1610	2714	91850	7142	7142	sace_1_ypr008w	91851	2040	119797	1074	93236	91852	93237	91853	5657	93238	91854	14345	2458	91855	14331
12382	2390	12383	12384	375	9414	12385	5932	12388	124040	1610	2714	91850	7142	7142	sace_3_5_e02840	91851	2040	2040	124041	1074	91852	91853	5657	91854	14345	2458	91855	14331	12155	159
203	12382	2390	12383	12384	375	9414	12385	5932	12388	1610	2714	91850	7142	7142	sapa_1_5_e03020	91851	2040	131382	1074	91852	91853	5657	91854	14345	2458	91855	14331	12155	159	174
2390	12383	12384	375	9414	12385	5932	12388	128674	128675	1610	2714	91850	7142	7142	sami_1_16.247	91851	2040	128676	1074	91852	128677	91853	5657	128678	91854	14345	2458	91855	14331	12155
203	12382	2390	12383	12384	375	9414	12385	5932	12388	1610	2714	91850	7142	7142	saku_1_16.292	91851	2040	127972	1074	91852	94690	91853	5657	91854	14345	2458	91855	14331	12155	159
203	12382	2390	12383	12384	375	9414	12385	5932	12388	1610	2714	91850	7142	7142	saar_1_6_f02790	91851	2040	117469	1074	91852	91853	5657	91854	14345	2458	91855	14331	12155	159	174
0	0	2390	12383	12384	375	9414	12385	5932	12388	1610	2714	91850	7142	7142	saba_1_11_k00230	91851	2040	117518	1074	91852	91853	5657	91854	14345	2458	91855	14331	12155	159	174
0	0	0	0	0	0	0	0	0	0	0	0	0	0	0	saba_2_351_mm00100	91851	2040	0	0	0	0	0	0	0	0	0	0	0	0	0
203	12382	2390	12383	12384	375	9414	12385	5932	12388	1610	2714	91850	7142	7142	sauv_1_16.328	91851	2040	134619	1074	91852	134620	134621	91853	5657	134623	91854	14345	2458	91855	14331
9511	9209	10785	65502	12389	12388	5932	12386	12385	9414	2278	2714	65503	7142	7142	kaaf_1_b01950	559	112806	1074	65504	65505	5657	65506	1208	14331	12155	159	265	14327	65507	2989
10785	66616	12389	12388	5932	12386	12385	66615	9414	2278	1610	2714	66614	7142	7142	kana_1_d02340	66613	2040	112903	1074	66612	66611	5657	66610	1208	2692	14331	12155	159	265	14327
13118	1993	13117	13117	83746	13114	11138	13112	13110	83745	37350	83744	83743	83742	83741	naca_1_b05730	5917	8268	14034	14035	11735	13922	83740	83564	4802	2458	6428	13923	13924	13925	83739
0	0	13117	13117	83746	13114	11138	13112	13110	83745	37350	83743	83742	83742	83741	naca_2_9_i00230	5917	84395	14034	14035	11735	13922	83740	83740	83564	37203	4802	2458	6428	13923	13924
13118	84562	88	15	3432	13117	13117	84561	13114	11138	13112	13110	84560	37350	84559	nada_1_b03070	134	13922	11735	14035	14034	84558	5917	37203	4802	2458	6428	13923	13924	13925	84557
1208	2278	9414	12385	5932	12388	12389	10785	9209	9511	1610	2714	37366	7142	7142	cagl_1_l09339g	37368	37369	37370	11191	15020	694	7710	14463	14459	12535	14113	14114	14115	399	10462
1208	2278	9414	12385	5932	12388	12389	10785	9209	9511	1610	2714	37366	7142	7142	cagl_2_2_b04230	37368	37369	37370	11191	15020	694	7710	14463	14459	12535	14113	14114	14115	399	10462
13693	1056	13695	13696	13697	97495	13701	13702	15983	15983	1834	12211	15	559	12210	tebl_1_c00170	15140	97493	65659	55	15	0	0	0	0	0	0	0	0	0	0
1541	12377	12378	5368	98441	98440	375	1208	2278	12385	12389	98439	98438	10785	98437	teph_1_d00690	153	36908	98436	15143	559	7000	12376	12375	12374	12372	98435	98434	768	13352	135184
12217	16247	525	13390	9735	1522	344	8754	13736	13735	13733	100274	83453	14717	100246	vapo_1_541.30	153	100275	100276	100277	15143	559	2040	135399	1074	100278	2911	14333	65652	100279	1619
5932	12388	12389	104089	10785	9209	9511	99185	7142	7142	104090	12352	2714	1610	104091	zbist_2620	15140	153	104698	104699	104093	11068	37013	669	13444	10790	2458	14615	12254	18528	1033
5932	12388	12389	104089	10785	9209	9511	99185	7142	7142	104090	12352	2714	1610	104091	zyba_3_9_i00670	15140	153	104740	104699	104093	11068	37013	669	13444	10790	2458	14615	12254	18528	1033
104089	10785	9209	9511	135956	12562	99185	7142	7142	104919	12352	2714	1610	104091	15938	zyro_1_f04862g	15140	153	104699	104093	11068	37013	669	13444	10790	2458	1033	12217	1204	15983	13443
12386	5932	12388	12389	99183	10785	9209	9511	1610	2714	12352	99184	7142	7142	99185	tode_1_c01350	15140	153	99186	65659	11068	37013	669	13444	10790	99187	12597	3170	99188	1660	669
72002	618	13889	55	2147	71760	72001	13888	72000	71999	71998	9407	113804	13887	71997	lawa_1_14.1543	15140	71996	153	153	113803	71995	71994	113802	71715	71993	71992	71991	71990	71716	113801
12850	9574	12849	12848	71712	12846	174	1610	2714	12352	71713	7142	70559	12457	71714	lath_1_g16984g	15140	153	71715	71716	17557	15143	559	2040	13709	36841	14716	5245	14181	2539	37228
5932	12388	12389	70557	10785	9209	9511	1610	2714	12352	70558	7142	7142	70559	12457	lakl_1_b03344g	15140	153	70560	70561	70562	15143	559	2040	13709	36841	14716	5245	14717	70563	14719
14181	67275	12667	67274	3694	13612	67273	67272	5478	15135	67271	15137	7142	67270	12457	klae_1_13_m00540	153	67269	67268	15143	559	36841	13709	134	10836	12502	14835	14836	14837	13671	7790
0	0	0	68767	3694	13612	67273	67272	5478	15135	67271	15137	7142	68766	12457	klwi_1_120_dp00120	153	68765	0	0	0	0	0	0	0	0	0	0	0	0	0
14181	67944	12667	67274	3694	13612	67273	67272	5478	15135	67271	15137	7142	67945	12457	klma_1_3_c06150	153	67269	67268	15143	559	36841	13709	134	10836	12502	19101	67673	11038	12589	13842
14181	67944	12667	67274	3694	13612	67273	67272	5478	15135	67271	15137	7142	67945	12457	klla_1_a03047g	153	67269	67268	15143	559	36841	13709	134	10836	12502	19101	67673	11038	13842	14551
12671	12670	12669	12668	12667	12666	3694	13612	15133	15135	15136	15137	7142	15138	12457	ergo_1_ael295c	15140	153	15141	15142	15143	559	55245	55246	15145	7781	285	15146	15147	15148	15149
1337	3442	15150	15149	15147	15146	285	7781	15145	55064	13709	55065	559	15143	12457	ercy_1_6116	15140	153	55066	55067	55068	15141	15142	15138	7142	15137	15136	15135	55069	15133	13612
15128	15129	15130	348	15131	15132	13612	15133	15134	15135	15136	15137	7142	15138	12457	asac_1_7_g04120	15140	153	15141	15142	15143	559	15144	15145	7781	285	15146	15147	15148	15149	15150

Additional file 5.
